# Deep-learning-based pyramid-transformer for localized porosity analysis of hot-press sintered ceramic paste

**DOI:** 10.1371/journal.pone.0306385

**Published:** 2024-09-04

**Authors:** Zhongyi Xia, Boqi Wu, C. Y. Chan, Tianzhao Wu, Man Zhou, Ling Bing Kong

**Affiliations:** 1 College of Applied Technology, Shenzhen University, Shenzhen, Guangdong, China; 2 College of New Materials and New Energies, Shenzhen Technology University, Shenzhen, Guangdong, China; 3 Key Laboratory for Comprehemsive Energy Saving of Cold Regions Architecture of Ministry of Education, Jilin Jianzhu University, Changchun, Jilin, China; Semnan University, ISLAMIC REPUBLIC OF IRAN

## Abstract

Scanning Electron Microscope (SEM) is a crucial tool for studying microstructures of ceramic materials. However, the current practice heavily relies on manual efforts to extract porosity from SEM images. To address this issue, we propose PSTNet (Pyramid Segmentation Transformer Net) for grain and pore segmentation in SEM images, which merges multi-scale feature maps through operations like recombination and upsampling to predict and generate segmentation maps. These maps are used to predict the corresponding porosity at ceramic grain boundaries. To increase segmentation accuracy and minimize loss, we employ several strategies. (1) We train the micro-pore detection and segmentation model using publicly available Al_2_O_3_ and custom Y_2_O_3_ ceramic SEM images. We calculate the pixel percentage of segmented pores in SEM images to determine the surface porosity at the corresponding locations. (2) Utilizing high-temperature hot pressing sintering, we prepared and captured scanning electron microscope images of Y_2_O_3_ ceramics, with which a Y_2_O_3_ ceramic dataset was constructed through preprocessing and annotation. (3) We employed segmentation penalty cross-entropy loss, smooth L1 loss, and structural similarity (SSIM) loss as the constituent terms of a joint loss function. The segmentation penalty cross-entropy loss helps suppress segmentation loss bias, smooth L1 loss is utilized to reduce noise in images, and incorporating structural similarity into the loss function computation guides the model to better learn structural features of images, significantly improving the accuracy and robustness of semantic segmentation. (4) In the decoder stage, we utilized an improved version of the multi-head attention mechanism (MHA) for feature fusion, leading to a significant enhancement in model performance. Our model training is based on publicly available laser-sintered Al_2_O_3_ ceramic datasets and self-made high-temperature hot-pressed sintered Y_2_O_3_ ceramic datasets, and validation has been completed. Our Pix Acc score improves over the baseline by 12.2%, 86.52 vs. 76.01, and the mIoU score improves from by 25.5%, 69.10 vs. 51.49. The average relative errors on datasets Y_2_O_3_ and Al_2_O_3_ were 6.9% and 6.36%, respectively.

## Introduction

Due to their outstanding mechanical, thermal and dielectric properties, advanced ceramics have shown enormous potential in various applications, including aerospace facilities, biomedical implants, optoelectronic devices and so on. Ceramics are polycrystalline materials characterized by high melting point, hardness and wear resistance. To characterize the microstructure ceramic materials, scanning electron microscopy SEM is usually used to observe and analyze the morphology and size distribution of grains [1].

SEM images of ceramic materials consist of grains, e pores and grain boundaries. For insulation applications, ceramics are prone to electrical breakdown at high voltages [2]. One of the ways to increase breakdown voltage is to reduce the grain size as small as possible, thus having more grain boundaries [3–6]. Also, mechanical properties are important parameters for structural applications, such as strength, hardness, ductility and compressibility, which are directly dependent on grain size, size distribution, morphology and porosity [7]. Therefore, it is essential to accurately characterize the microstructure of ceramic materials, especially according to SEM images [8, 9].

Currently, the analysis of SEM images relies mainly on manual methods, which have obvious limitations. On one hand, in an individual SEM image, since there are a large number of ceramic grains, manual counting is time-consuming, inefficient and highly labor-intensive. On the other hand, the irregular shapes and low contrast of the grains in SEM images make manual counting susceptible to subjective bias, leading to skewed results [10]. Therefore, there is a strong need for automated methods to study the microstructure of ceramic materials. Given the characteristics of SEM images, including rich edge information, a lack of texture information, non-uniform grain sizes, irregular shapes and low image contrast, image segmentation could be an effective technology for grain segmentation followed by grain size distribution statistics.

With the rapid development of artificial intelligence, deep learning-based methods have gained widespread attention in image segmentation. Unlike traditional image segmentation methods, deep learning approaches essentially classify pixels in images to obtain pixel-level dense classifications. Initially, edge-detection algorithms [11] were used to segment grains, but they were not ideal for extracting grains with unclear contours. To more effectively extract grains with closed contours, Vincent et al. [12] enhanced edge information and then used the watershed algorithm for grain segmentation. However, this method suffered from the problem of "over-segmentation." Therefore, Heilbronner et al. [13] proposed an automatic grain boundary detection method based on gradient filtering. However, the quality and quantity of input images had a significant impact on the results. These methods employed conventional image processing techniques for grain segmentation, but the non-uniform grayscale inside ceramic grain SEM images, irregular grain sizes and shapes and the presence of pores, also affect segmentation results, making segmentation accuracy unpredictable.

Inspired by the success of Convolutional Neural Networks (CNNs) [14] in tasks such as medical image segmentation [15] and remote sensing image segmentation [16], Jiang et al. [17] proposed a sandstone grain segmentation method based on neural networks and fuzzy clustering algorithms, significantly improving the segmentation accuracy on datasets of this nature. Boyuan et al. [18] introduced an enhanced U-Net neural network architecture and weighted loss function, which improved grain boundary detection accuracy and was suitable for polymeric material segmentation. For ceramic grain segmentation, due to the distinct nature of feature extraction and spatial localization of grains, it is impossible to directly transfer CNN methods used for image classification to image segmentation. In CNNs based on the encoder-decoder neural network architecture, the specifics of image segmentation were considered. For example, U-Net with a symmetrical encoder-decoder structure fuses deep semantic features with fine-grained shallow information, producing more accurate segmentation images. The use of multi-layer transpose convolution networks in the decoder of Deconvolution Network (DeconvNet) [19] improves the determination of target’s coarse and detailed information. A Deep Convolutional Image Segmentation Network (SegNet) [20] retains spatial position information, known as pooling indices, during encoding phase, while pooling indices are utilized to recover more accurate target positions during the decoding phase. Inspired by skip connections in Residual Network-based (ResNet) [21], multi-branch structures were introduced to increase network connectivity diversity and fuse features of different scales. While CNN-based deep learning methods have excellent feature representation capabilities, they have inherent limitations in modeling long-range dependencies, due to the local nature of convolution operations. In cases involving weak textures, significant changes in object shapes and substantial differences in object sizes, pure CNN-based methods typically yield suboptimal results. The development of similar tasks has been driven by Transformer’s self-attention mechanism and its competitive modeling capabilities. The first pure Transformer design applied directly to a series of image patches for classification tasks was proposed by Dosovitskiy and colleagues as Vision Transformer (ViT) [22]. Several upgraded versions of Transformers have been reported, including Swin Transformer [23] and Dense Prediction Transformer (DPT) [24], which have shown outstanding performance in computer vision tasks with high computational costs similar to ViT. Wang et al. [25] designed a Pyramid Vision Transformer (PVT) suitable for dense prediction tasks. It overcame challenges associated with transplanting Transformers into various dense prediction tasks, making it a unified backbone for various visual tasks. Wang et al. [26] introduced attention mechanisms into their pore analysis method for Al_2_O_3_ ceramics, addressing the issue of CNN’s lack of a global receptive field and significantly improving segmentation accuracy. Recently, Zhou et al. [27] proposed a simple and efficient one-stage solution to directly extend CLIP’s zero-shot prediction capability to the pixel level, thereby addressing pixel-level zero-shot semantic segmentation tasks.

Through analysis of the latest SEM image segmentation algorithms, we have identified several key issues with current technology: (1) Manual marking of ceramic porosity in SEM images is inefficient due to the irregular particle shapes, and manual counting is susceptible to subjective bias. (2) If deep learning methods are used instead of manual labeling, challenges arise from sparse data and uneven label quality. Therefore, acquiring and preparing high-precision SEM image data has become one of the key issues in applying deep learning to the ceramic field. (3) SEM images of ceramic materials primarily exhibit particle outlines, lacking fine texture details, making direct application of traditional image segmentation algorithms for particle segmentation difficult. (4) CNN-based methods excel at capturing edge information and texture details, but when dealing with SEM images with extremely high resolution, they inevitably lose details during the pooling process, reducing their ability to perceive small-scale features and spatial local structures.

To address the issues of sparse datasets and uneven label quality, we captured Y_2_O_3_ ceramic SEM images, preprocessed and labeled them to create a high-quality Y_2_O_3_ ceramic dataset, which was then combined with the publicly available Al_2_O_3_ ceramic dataset for PSTNet training.

In response to the challenges of applying deep learning to the ceramic field, we propose PSTNet, an encoder-decoder architecture that integrates Transformers into a Feature Pyramid Network (FPN), using PVT as the encoder. The self-attention mechanism endows the model with a global receptive field during training. This method obtains multiscale feature maps through step-by-step downsampling during encoding, combining shallow and deep semantic information to capture image outlines and texture details. In the decoding phase, the improved MHA accomplishes multiscale feature fusion and outputs the segmentation image.

## Related works

### Pyramid vision transformer

Due to the limitations of convolution mechanisms in CNNs, whether using 3x3 convolutions or larger kernels, it’s impossible to capture the complete global information. Therefore, when using FPN, partial loss of global receptive field is inevitable. In each FPN block, the height and width of the feature maps are halved and the number of channels is doubled. Additionally, a convolution operation with a stride of 3 is used for pooling to increase the receptive field. In contrast, ViT tokenizes the feature maps and continuously stacks encoder blocks to achieve a global receptive field. This approach is feasible for image classification. However, when applied to dense prediction tasks, the following issues arise. (1) Semantic segmentation and depth estimation typically require higher-resolution inputs. As the input images become larger, the computational complexity of ViT increases significantly. (2) ViT uses relatively large patches for tokenization, such as using patches of size 16, which results in coarser features that lead to a loss of valuable information for dense tasks.

Wang et al. [25] proposed PVT to address these challenges. PVT employs a Transformer hybrid pyramid architecture, dividing the network into multiple stages. In each stage, the feature maps have their height and width halved as compared with the previous stage, which reduces the number of tokens by a factor of four. Furthermore, to further reduce computational complexity, PVT replaces the conventional MHA with spatial-reduction attention (SRA). The core of SRA is to reduce the number of key-value pairs in the attention layer. While conventional MHA has a number of key-value pairs equal to the sequence length during attention layer computation, SRA reduces it to 1/R^2^. Although PVT does not significantly outperform ViT in terms of accuracy, it substantially reduces computational complexity. Moreover, it can output multi-scale feature maps, which is crucial for segmentation and object detection tasks. Given that most current segmentation and detection models use the FPN structure, PVT’s feature allows it to seamlessly serve as an alternative backbone network to connect with segmentation and detection heads, replacing traditional CNNs.

### Porosity

The proportion of intergranular voids, often referred to as porosity, directly impacts the mechanical properties of ceramic materials, such as strength, hardness, toughness and compressive resistance. It also indirectly affects key characteristics like thermal conductivity, insulation and corrosion resistance. Therefore, quantitatively plotting variation curves to evaluate the surface porosity of ceramics stand as a crucial method for assessing the quality of ceramics.

Nonetheless, predicting high-resolution local porosity still poses challenges. This is primarily attributed to the limitations of existing porosity prediction methods, which often provide only average values [28]. Software applications, such as Image Pro Plus, ImageJ and Ipwin32, have been attempted to analyze porosity of materials by processing microscopic images [29]. While these methods can partially segment SEM images, they fall short due to their inability to effectively distinguish individual ceramic components in the images [30]. Additionally, SEM images of ceramics exhibit a pseudo-3D nature. Therefore, relying solely on image processing software for segmentation yields suboptimal results.

Furthermore, real-time adjustments and optimization of chosen thresholds within the software, coupled with the calculation of the percentage of segmented pores in microscopic images to assess porosity at specific locations, have been achieved. However, the accuracy of image processing-based methods is limited. This is attributed to the dependency of pore segmentation heights on threshold selection, leading to significant threshold errors owing to the human judgment. Moreover, the segmentation process primarily relies on pixel values rather than feature-driven pore morphology, resulting in the unnecessary detection of features, such as grain boundaries and defects. Furthermore, the image segmentation process lacks standardized performance evaluation criteria, making it challenging to optimize porosity segmentation results.

Therefore, when dealing with the segmentation of complex-shaped grains and pores, the use of deep learning methods becomes particularly crucial. Recently, deep learning techniques have made significant advancements in the segmentation of SEM images, especially for ceramics. Zhang et al. [31] introduced a deep learning-based porosity monitoring method for laser additive manufacturing processes. They employed a CNN model to learn the relationship between laser-induced melt pool images and the distribution of porosity within the melt pool. Zhou et al. [32] used a DeepLabV3+ network to evaluate the correlation between original images and annotated pore images, aiming to analyze the porosity distribution in concrete. Compared with traditional imaging methods, these DL-based models exhibit higher feasibility and efficiency in porosity analysis. Bangaru et al. [33] applied SEM images and semantic segmentation for the analysis of microstructures of concrete. They validated feasibility of the methods and achieved remarkably high accuracy across seven concrete samples’ SEM images.

Taking into account the aforementioned improvements in segmentation frameworks, we have implemented a series of enhancements to the model itself, the training strategy and the dataset. (1) We utilized Wang et al.’s Al_2_O_3_ dataset to train and validate our model, enabling the estimation of mechanical properties, such as strength, hardness, and toughness of the Al_2_O_3_ ceramics. (2) We captured and established our Y_2_O_3_ dataset for predicting grain sizes and their distribution in Y_2_O_3_ ceramics, evaluating physical properties such as thermal conductivity and insulation. (3) We developed a novel encoder-decoder architecture that combines the advantages of Transformers and CNNs. We used PVT as the backbone for dense prediction tasks, extracting multiscale feature maps. Through embedding, upsampling, resampling and a series of convolutional operations, we restored multiscale feature maps to the same resolution and integrated multilayered information for image segmentation tasks.

## Method

**Fig 1:**
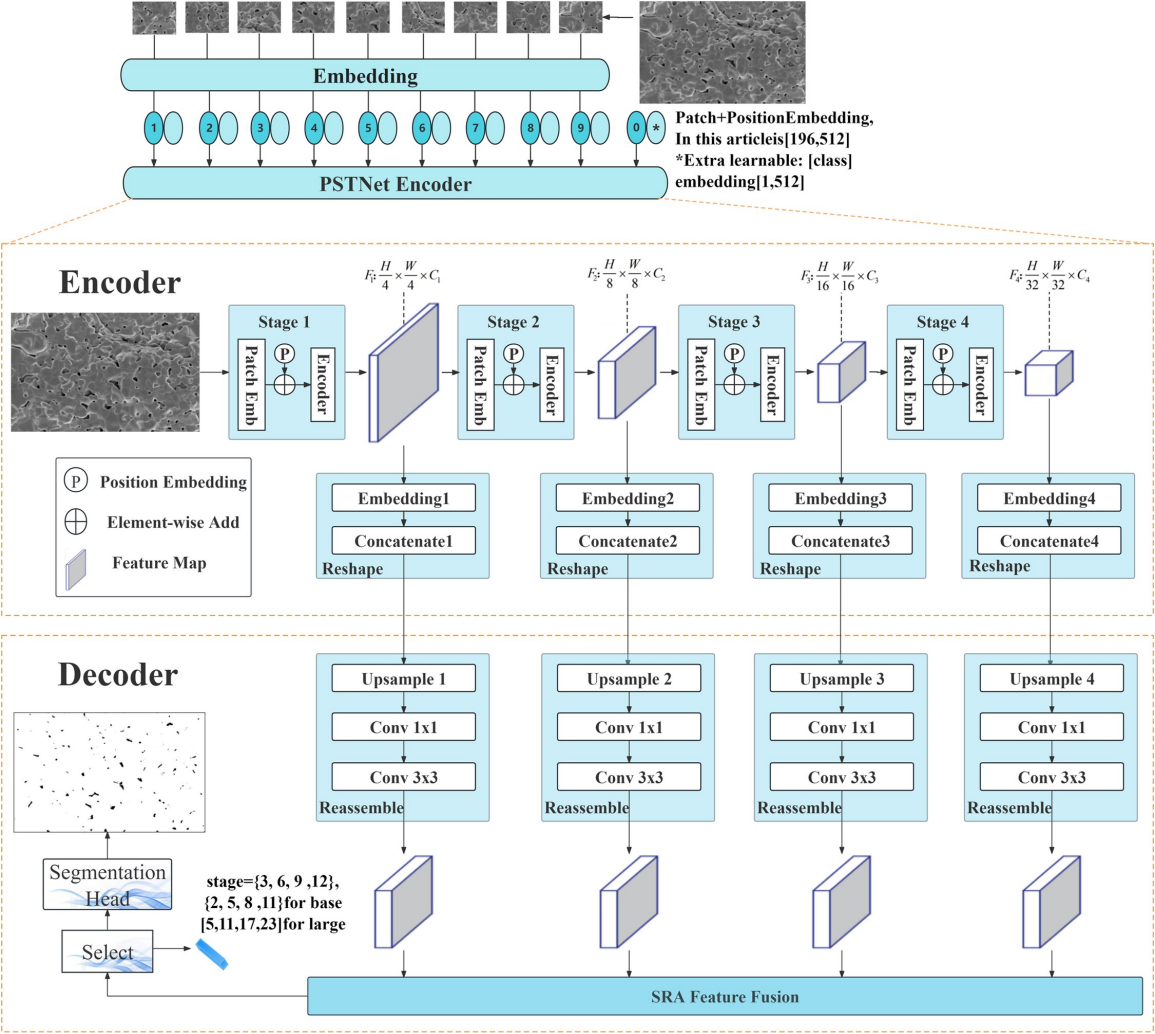
Architecture overview. During the encoding stage, the input image is first subjected to Embedding processing to meet the requirements of the encoder. Subsequently, through the progressive downsampling connection of four encoders, the input image is transformed into feature maps with multi-scale feature information. These feature maps are reshaped into 2D tokens to meet the requirements of attention mechanisms. Then, during the decoding stage, the tokens undergo a reassembling process, followed by upsampling using transpose convolution, channel transformation through 1x1 convolutions, and size adjustment using 3x3 convolutions. We employ an improved version of attention mechanisms to effectively fuse shallow and deep semantic information. Finally, the fusion block is subjected to another round of transpose convolution to restore it to the initial resolution, and after processing by the Segmentation Head, the final segmentation map is produced.

This section introduces PSTNet (Fig 1). we maintain the overall encoder-decoder architecture that has been successful in dense prediction in the past, utilizing the PVT as a backbone, and show how representations generated by this encoder can be efficiently transformed into dense predictions.

Feature extractor consists of an encoder and an Embedding layer. After the Embedding layer, the image undergoes multiple Encoder layers for feature extraction and obtains 3D feature maps. Since subsequent Encoder layers are built upon the previous ones, these feature maps possess different semantic information. These feature maps with shallow and deep semantic information will be transformed into 2D tokens under the action of Embedding, preparing for subsequent deconvolution upsampling and feature fusion.

The decoder comprises upsampling blocks, convolutional blocks, and feature fusion blocks. Semantic information from different dimensions will be integrated during the decoder stage. Changes through upsampling and convolution enable these tokens to be restored to the same resolution for feature fusion. In feature fusion, we provide two approaches: One is based on ResNet’s residual network fusion method, which has fewer parameters, while the other is based on MHA fusion method, which has higher accuracy.

The advantages of incorporating Transformers into the feature pyramid’s step-down sampling process are evident. The feature maps obtained after gradual downsampling contain both shallow and deep semantic information, which is crucial for dense prediction tasks. The self-attention mechanism of Transformers equips the training process with a global receptive field, facilitating a better understanding of objects and relationships within the context. Unlike the approach of PVT, we choose to introduce the class token before encoding, which is vital for maintaining the overall network consistency. Although it is possible to obtain segmentation and classification information directly by connecting the last-level feature map F4 to MLP heads or segmentation heads after step-down sampling, this approach essentially resembles CNN, differing mainly in terms of computational complexity and time efficiency. In contrast, the fusion of multiscale feature maps and subsequent upsampling to achieve the final dense prediction substantially reduces computational load while maintaining a remarkably high level of accuracy.

### Encoder

#### Multi-head attention

**Fig 2:**
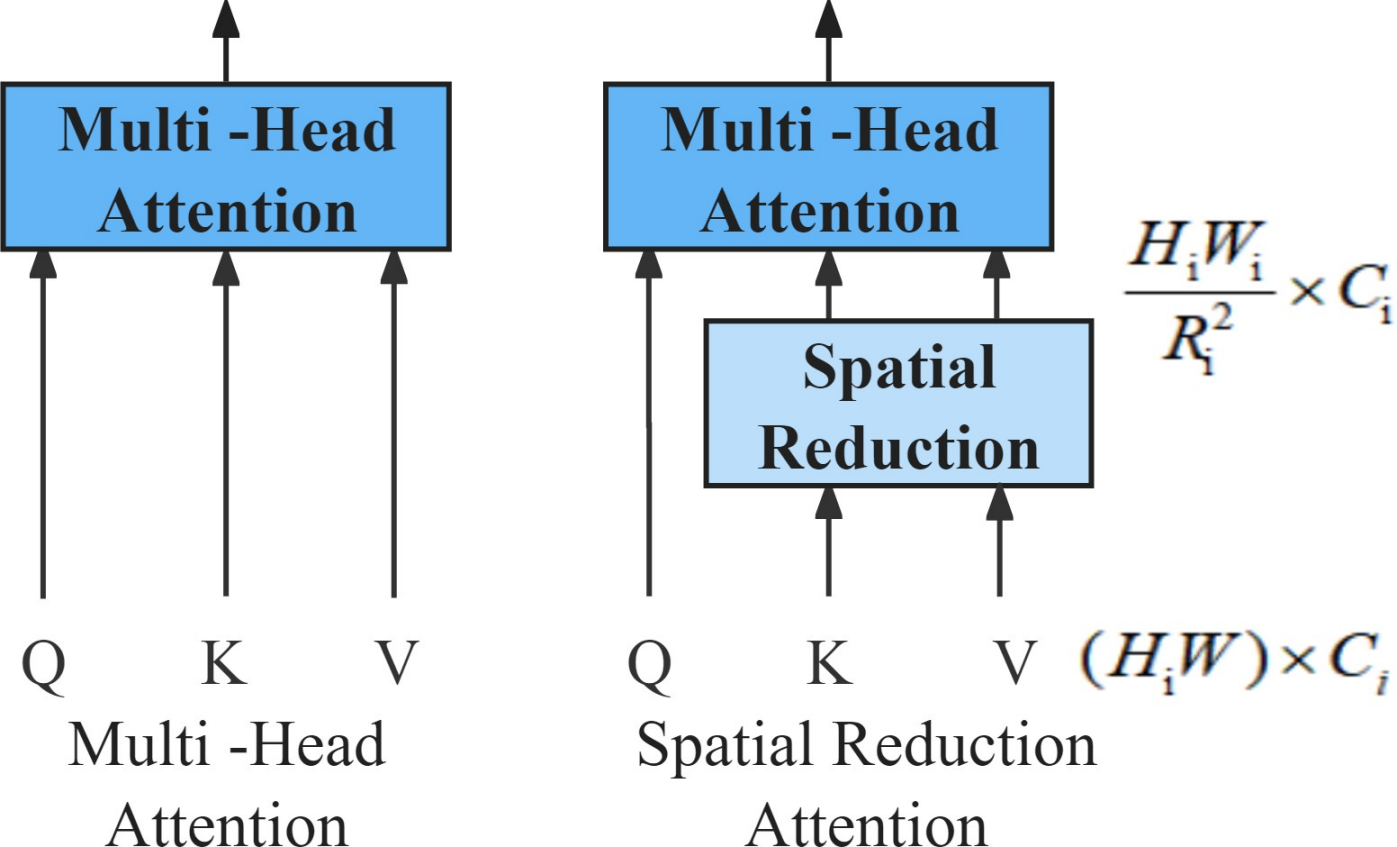
*Left*: MHA Block. *Right*: Linear SRA Block.

SRA [25] is built upon the MHA [34], aiming to further reduce computational complexity by decreasing the number of key-value pairs in the attention layer (Fig 2). In conventional MHA, the number of key-value pairs is equivalent to the length of the sequence, with a time complexity of $\Omega (MHA)=4LC^{2}+2L^{2}C$; however, SRA divides the feature map into patches, linearly transforming patches into $\frac{HW}{R^{2}}\times C$, thereby reducing the number of key-value pairs to $\frac{1}{R^{2}}$ of the original count. Linear SRA [35] is an improved version of SRA, achieving resolution reduction by replacing convolutional operations with a combination of pooling and convolution operations. Its time complexity is $\Omega (Linear\;SRA)=2hwP^{2}c$ [34].

The lower time complexity can accelerate model training, which is one of the reasons why we choose Linear SRA. More importantly, Linear SRA can be applied to Feature Fusion in the decoding stage. Compared with traditional ResNet residual connections, MHA can retain richer feature details. With the assistance of deconvolution or bilinear interpolation, these fused features will be restored to their initial shape and dimensions, thereby outputting the segmentation map.

The coding phase can be summarized by the following five formulas [22, 24]:

The purpose of Embedding is to convert the three-dimensional feature maps into two-dimensional tokens to meet the shape requirements of attention [22, 34].

$$E\text{mbedding}:\;{\mathbb{R}}^{\frac{H}{P}\times \frac{W}{P}\times D}\to {\mathbb{R}}^{N_{P}\times D}$$ (1)

where P represents the patch size, controlling the number of divisions in the original image, H represents height, W represents width, *N*_*P*_ represents the number of tokens after embedding and D represents the dimensional space. After Embedding, the image is transformed into a two-dimensional *N*_*P*_×*D* format. When the input image format is *H*×*W*×3, we use a fixed Patch value to split the image into *N*_*P*_ blocks for the encoder.

$$z_{l}=MLP\left(LN\left(z_{l}^{\prime }\right)\right)+z_{l}^{\prime },l=1\ldots L$$ (2)

where *z*_*l*_ is the output of the current encoder block and ${z^{\prime }}_{l}$ is the output of the spatial-reduction attention mechanism. The MLP layer is not essential for semantic segmentation. However, in our experiments, we set the Dropout within it to 0.1 to enhance robustness.

$$z_{l}^{\prime }=Linear\;SRA\left(LN\left(z_{l-1}\right)\right)+z_{l-1},1\ldots L$$ (3)

where *z*_*l*-1_ is the output of the previous Transformer encoder block and Linear SRA is a variant of MHA.

$$z_{0}=\left\{X_{class};X_{P}^{1}E,X_{P}^{2}E;\ldots X_{P}^{N}E\right\}+X_{pos},E\in {\mathbb{R}}^{\left(P^{2}\cdot C\right)\times D},E_{pos}\in {\mathbb{R}}^{\left(N+1\right)\times D}$$ (4)

where *X*_*class*_ is the trainable label, $X_{P}^{N}E$ is N 2D Patches with resolution *P*×*P* for either 196 or 576, E is the trainable projection, and *E*_*pos*_ is the positional embedding.

$$y=LN\left(z_{L}^{0}\right)$$ (5)

where $z_{L}^{0}$ is the learnable classification embedding, LN is Layer Normalize, which normalizes the input feature map, and y is the classification result.

### Decoder

According to the position of the initial patch, it is put into the corresponding position respectively to get the corresponding feature expression [24].

$$Concatenate:{\mathbb{R}}^{N_{P}\times D}\to {\mathbb{R}}^{\frac{H}{P}\times \frac{W}{P}\times D}$$ (6)

where P represents the patch size, controlling the number of divisions in the original image, H represents height, W represents width, *N*_*P*_ represents the number of tokens after embedding and D represents the dimensional space.

A 1x1 convolution is used to change the channel, followed by a 3x3 convolution to resize [24].

Resamples:ℝHP×WP×D→ℝHS×WS×D^ (7)

where *D* represents the dimensions of the image before upsampling, $\hat{D}$ represents the dimensions after upsampling, and S denotes the token that is assembled into a feature map with the spatial resolution of the $\frac{1}{s}$ input image.

In the reorganization and fusion phase of the feature map, we draw on the approach in, while the formula [24] can be expressed as:

ReassemblesD^t=Resamples∘Concatenatet (8)

#### Feature fusion

**Fig 3:**
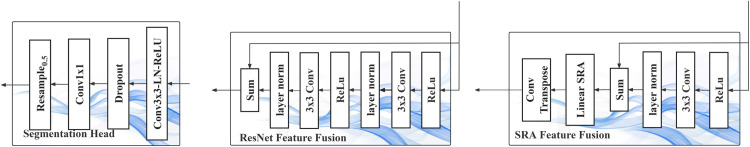
*Left*: Segmentation head. The feature maps go through a 3x3 convolutional alteration channel with dropout set to 0.1, and after a 1x1 convolutional alteration channel we upsample the fused feature maps using resample to obtain a final resolution of 1/S relative to the original resolution. Depth estimation and semantic segmentation used different values of the S multiplier, S = 2 in segmentation. ***Center*: ResNet Feature Fusion.** After 3x3 convolution kernel, ReLu activation function and layernorm processing, multi-scale feature maps of the same resolution are fused. ***Right*: *Linear SRA* Feature Fusion.** After convolution and activation function processing, the feature maps are concatenated, and the output of the fusion module is fed into Linear SRA, which restores the features to the original resolution size through deconvolution or bilinear interpolation.

In Feature Fusion stage, we employed two different methods to generate segmentation maps (Fig 3) and connected them to the Segmentation Head to produce segmentation maps. One method is based on the classical ResNet residual convolution module, which has the advantage of a smaller parameter size. The other optional solution is the MHA scheme based on Linear SRA, which offers higher accuracy [19].

## Experiment and analysis

### Images acquisition

Laser sintering technology is commonly employed in the fabrication of high-temperature ceramic materials [36], such as Al_2_O_3_, Si_3_N_4_, ZrO_2_, Y_2_O_3_ and so on. These ceramic materials exhibit excellent heat resistance, corrosion resistance, and hardness, making them invaluable in applications across aerospace, chemical, electronics, medical devices, and other fields, especially in high-temperature and extreme environments.

The training and evaluation were based on publicly available datasets of Al_2_O_3_ ceramics and our in-house Y_2_O_3_ dataset (S1 File). The raw materials, sourced from (Jingdezhen, Jiangxi, China), consist of 99.8% Y_2_O_3_, 0.1% ZrO_2_ and 0.1% MgO. The samples were prepared by using a typical ceramic process [37]. Initially, the raw materials, including 9.9521 g Y_2_O_3_, 0.0103 g ZrO_2_, and 0.0091 g of MgO, were mixed by using ball milling process (Changsha, China). Subsequently, the mixture was calcined at 930°C for 5 h [38].

The calcined samples were further milled for 12 h. Subsequently, the milled powder was compacted at a pressure of 25 MPa for 1 min with a stainless-steel mold. The compacted samples were transferred into a graphite mold and then hot-pressed and sintered at 1100°C, during which the pressure was gradually increased to 30 MPa. The samples were sintered at 1080–1125°C for 4 h. The sintered samples were annealed in air at 925°C for 24 h, followed by grinding and mirror polishing (to 1.0 mm) on both surfaces. The finely polished sintered samples were observed by using SEM.

#### Data preprocessing

**Fig 4:**
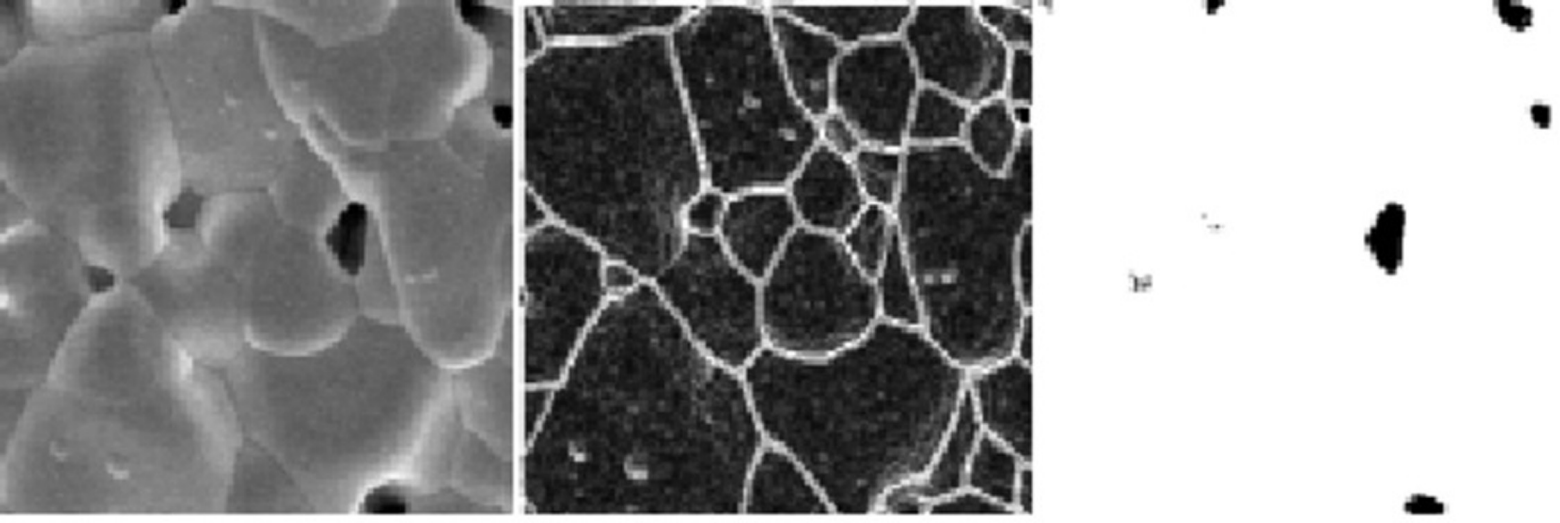
*Left*: Original image. Prior to training, manual labeling was performed on the training dataset for the target object (i.e., surface grains). To ensure detection and segmentation accuracy, we defined labeling rules based on the features of surface pores and associated pixel values in the SEM images. After laser sintering, surface defects usually appear on the grains and these regions are pixel less than 10 parts in the grayscale SEM image. In order to better utilize the pixel values to identify surface grains, the minimum number of consecutive pixels required to mark surface defects is therefore set to 10 to prevent defects from leading to reduced accuracy. ***Center*: A preprocessed image after contrast adjustment. *Right*: Segmentation image.** The model correctly distinguishes between grains and pores, with pixel values in the 245–255 part at the pores and uncertain regions predicted according to pixel values.

We captured 10 SEM images of Y_2_O_3_ at different positions, maintaining a magnification factor of 2.0k. Each image had a resolution of 1280 × 960 pixels. To increase the size of the SEM dataset, we segmented the SEM images into smaller images of 256 × 256 pixels (Fig 4). This segmentation served the dual purpose of reducing the number of target objects in each training image and augmenting the dataset. Such measures are crucial for enhancing training efficiency and model accuracy. The chosen segmentation size was carefully selected. If it were too large, we would have an insufficient number of samples for training. If it were too small, there would be an insufficient number of particles in each small image, rendering the dataset inadequate in representing microstructural features, such as particle size, porosity and relative density. After segmentation, we obtained a total of 180 images.

To further expand the dataset and prevent overfitting, we rotated each image by 90º, 180º and 270º and performed random cropping, resulting in a total of 500 samples. The dataset was divided into 60% for training, 20% for validation and 20% for testing to evaluate the model performance.

### Loss function

Semantic segmentation [39] tasks belong to dense prediction classification tasks. Due to their discrete nature, for segmentation objectives, we choose cross-entropy as the loss function for quantifying model loss.

$$H_{p}\left(q\right)=\sum_{x}q\left(x\right)\log_{2}\left(\frac{1}{p\left(x\right)}\right)\;=-\sum_{x}q\left(x\right)\log_{2}\left(x\right)$$ (9)

where *p*(*x*) is the output of the neural network, *q*(*x*) is the correct solution label, and only the index of the correct solution label in *q*(*x*) is 1 (may be other values), the rest is 0, so the equation x only calculates the natural logarithm of the output of the correct solution label.

If the predicted value is $\hat{y}$ and the true value is *y*, then the Smooth L1 loss [40] is given by:

$$L_{smooth}=\left\{\begin{array}{l} 0.5\times (\hat{y}-y{)}^{2}\;\;\;if|\hat{y}-y|<1\\ |\hat{y}-y|-0.5\;\;\;\;\;otherwise \end{array}\right.$$ (10)

When $\left|\hat{y}-y\right|$ is less than 1, the Smooth L2 loss is equivalent to the standard L2 loss. When $\left|\hat{y}-y\right|$ is greater than or equal to 1, it becomes closer to the L1 loss. Therefore, compared to L2 loss, Smooth L1 loss is more robust to outliers.

SSIM [41] is a metric used to measure the similarity between two images, considering differences in luminance, contrast, and structure. The formula for SSIM is as follows:

$$L_{SSIM}=\left(x,y\right)=\frac{2\mu_{x}2\mu_{y}+C_{1}}{\mu_{x}^{2}+\mu_{y}^{2}+C_{1}}\times \frac{2\delta_{xy}+C_{2}}{\delta_{x}^{2}+\delta_{y}^{2}+C_{2}}$$ (11)

where *x* and *y* are the two images being compared, *μ*_*x*_ and *μ*_*y*_ are the mean values of images *x* and *y* respectively, σ_*x*_ and σ_*y*_ are the variances of images *x* and *y* respectively, σ_*xy*_ is the covariance between images *x* and *y*, *C*_1_ and *C*_2_ are constants added for stability.

### Model training

Our testing was conducted on an Ubuntu 22.04 system equipped with an Intel(R) Xeon(R) Silver 4210R CPU running at 2.40 GHz, 8 x 32 GB DDR4 RAM, and a TITAN XP with 8 x 12 GB of memory. The model training employed an NVIDIA TESLA V100 GPU. The code was implemented using Python 3.10 and PyTorch 2.0.0.

Compared with the AdamW [42] optimizer, the Stochastic Gradient Descent (SGD) [43] optimizer is more likely to cause PSTNet to fall into a local optimum, hindering further reduction of loss. Therefore, Adaptive Moment Estimation (Adam) [42] was chosen as the optimizer for the experiment. During pre-training, the learning rate was set to 0.01, and a learning rate scheduler was used to decrease it to 0.0001 after 10 epochs. This is because a sufficiently large learning rate is needed in the early stages of model training to allow the model to learn more semantic information. As epochs increase, this process should exhibit a slowing trend to enhance model stability and explore the lowest loss value. Dropout and Dropath (attentional random dropout) were set to 0.1, based on considerations of enhancing model robustness. The batch size was set to either 1 or 8. A lower batch size helps the model achieve better performance and stability. However, considering training costs, a batch size that is a multiple of 8 is more suitable (training a multiple of 8 images per epoch), which significantly improves model training speed.

**Fig 5:**
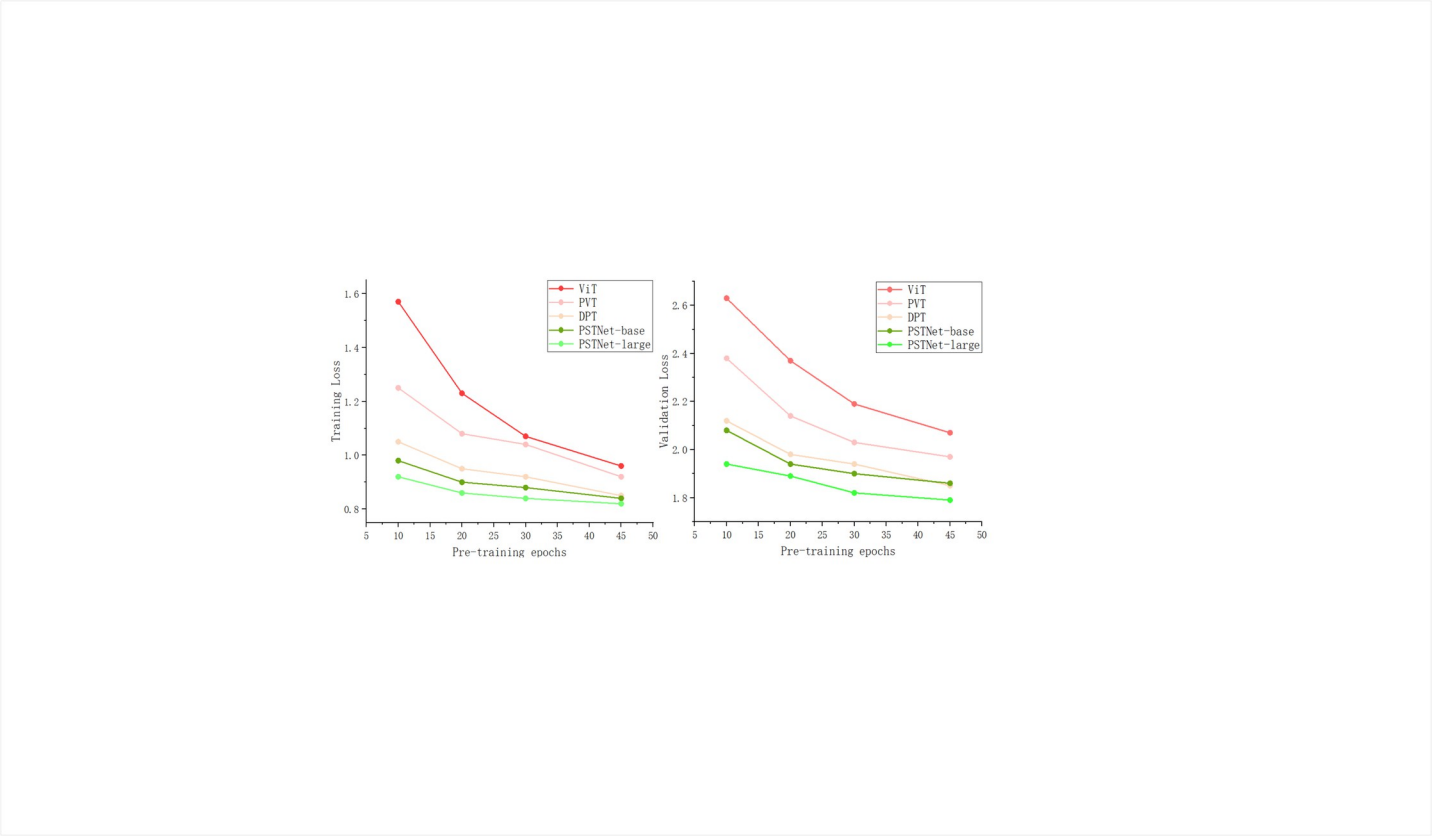
Training loss and validation loss for different methods.

We compared the training and validation losses of PSTNet-base, PSTNet-large, ViT [22], DPT [24], and PVT [25] models under various parameter settings to achieve the highest model performance. The patch size was set to 16, and input images were resized to tensors with a resolution of 384x384 to meet the requirements of the encoder. The learning rate for the backbone network was set to 1e-5, while the learning rate for the remaining parameters was 3e-4. Different methods in the experiment were trained for 10, 20, 30, and 45 epochs, respectively, to observe convergence behavior (Table 1 and Fig 5).

**Table 1:** Training loss and validation loss for different models.

Method	Epoch	Training Loss	Validation Loss
ViT	10	1.57	2.63
	20	1.23	2.37
	30	1.07	2.19
	45	**0.96**	**2.07**
PVT	10	1.25	2.38
	20	1.08	2.14
	30	1.04	2.03
	45	**0.92**	**1.97**
DPT	10	1.05	2.12
	20	0.95	1.98
	30	0.92	1.94
	45	**0.85**	**1.85**
PSTNet-base (This work)	10	0.98	2.08
	20	0.90	1.94
	30	0.88	1.90
	45	**0.84**	**1.86**
PSTNet-large (This work)	10	0.92	1.94
	20	0.86	1.89
	30	0.84	1.82
	45	**0.82**	**1.79**

Our approach exhibits faster convergence, higher stability (with reduced oscillation and peaks) and lower training and validation losses, as compared with other methods (Table 1 and Fig 5). During training, the following aspects can be summarized.

1. The experiment introduced attention in the decoder stage and trained partial parameters, resulting in lower model loss and validation loss compared to similar competing models.
2. Adjustments were made to some hyperparameters based on the two-stage training, enabling our model to have lower training and validation losses early in training (starting from epoch = 10).
3. SSIM loss, Smooth loss and Segmentation Penalty Cross-Entropy (SPCE) loss functions containing segmentation penalty terms are the main factors affecting model convergence and significantly enhancing model robustness.

**Fig 6:**
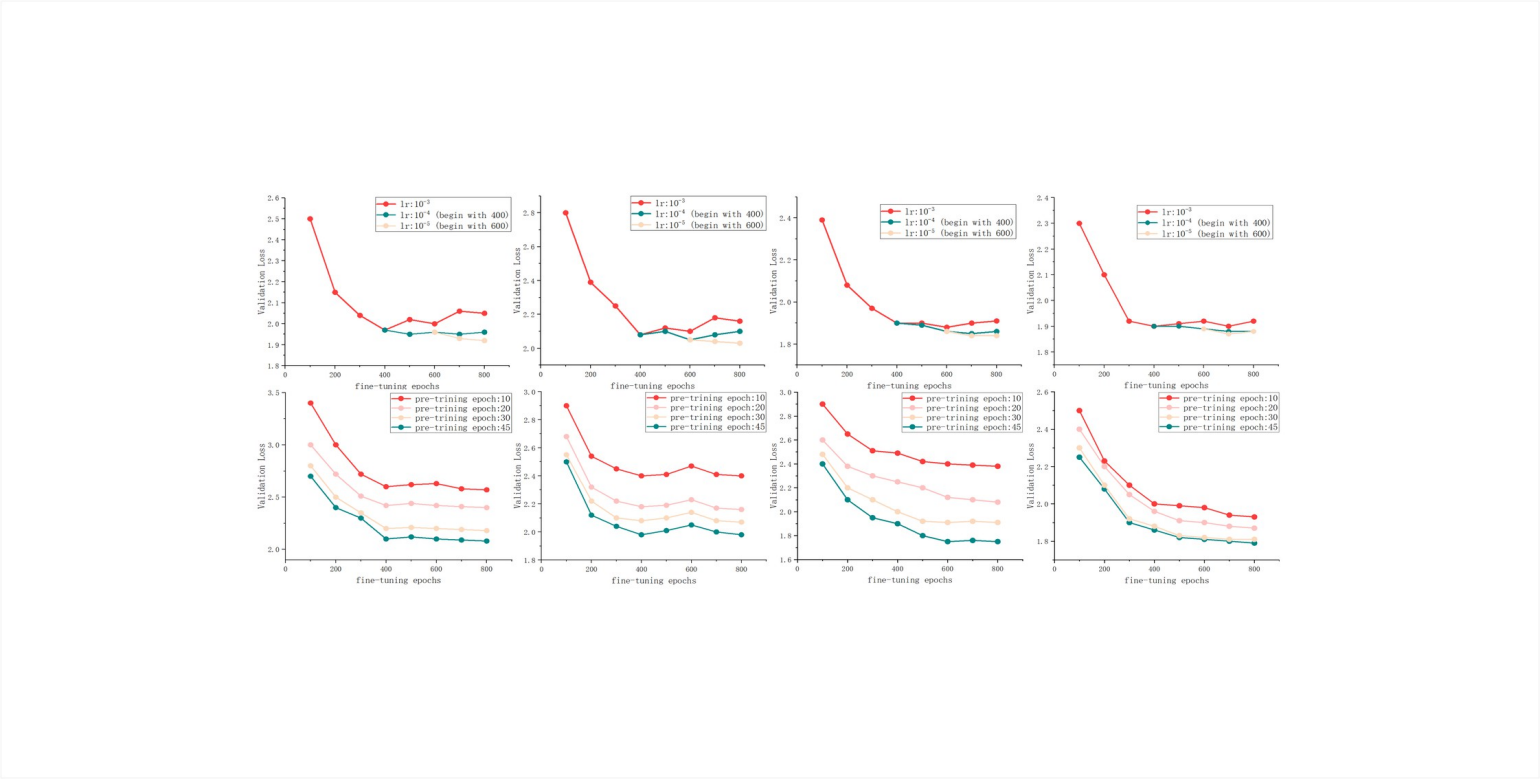
Fine-tuning process of different methods with the same training strategy: (a) ViT, (b) PVT, (c) DPT and (d) PSTNet.

We initially plotted all frameworks with different fine-tuning processes (epochs and learning rates) and trained models to analyze the relationship between the final results and fine-tuning processes. From the graphs (Fig 6), we can draw the following conclusions.

- Due to the high cost of SEM image acquisition, it is challenging to create large-scale datasets as in conventional images. Training models on small datasets cannot entirely eliminate overfitting, as shown in the first row. Therefore, it is not advisable to use pre-trained models with too many epochs for fine-tuning, as shown in the second row, where we utilized four pre-trained models with varying epochs for fine-tuning.
- Under higher learning rates, PSTNet demonstrates superior stability and better overfitting prevention capabilities. This can be attributed to its more rational network design, utilizing layer normalization based on consistency principles and setting dropout to 0.1.
- With the increase in epochs during the fine-tuning process, PSTNet maintains excellent stability. Despite the use of a larger learning rate multiplier in the experiments, the initial validation loss remains at a relatively low level.

### Evaluation metrics

The experiments were conducted on Al_2_O_3_ ceramic slurry dataset and custom Y_2_O_3_ ceramic dataset. We strictly followed the protocol established by Zhang et al. [44] and employed the cross-entropy loss function, while reporting pixel accuracy (pixAcc) and mean intersection over union (mIoU).

PSTNet achieves highly accurate results on the Y_2_O_3_ dataset (Table 2). Specifically, the present work’s Pix Acc (76.01 vs. 86.52) improves the mIoU by 12.2% (51.49 vs. 69.10) compared to the baseline (ViT). Compared to the suboptimal DPTNet, the present work improved mIoU by 12.2% (60.64 vs. 69.10).

**Table 2:** Segmentation accuracy of different methods on Y_2_O_3_ ceramic dataset.

Method		Higher is better		Lower is better
	model	Pix Acc[%]	mIoU[%]	Params
Ronneberger et al. 2015 [15]	U-Net	71.12	47.71	1.0G
Dosovitskiy et al. 2020 [22]	ViT	76.01	51.49	1.1G
Ranftl et al. 2021 [24]	DPTNet	81.83	60.64	614M
Wang et al. 2021 [25]	PVTNet	80.41	60.61	841M
Chen et al. 2017 [45]	OCNet	-	56.23	1.1G
Liu et al. 2019 [46]	ACNet	72.50	58.37	951M
Yuan et al. 2019 [47]	Deeplab V3	71.07	58.92	620M
Zhou et al. 2023 [27]	ZegCLIP	80.19	53.33	720M
The present work	PSTNet	86.52	69.10	201M

**Fig 7:**
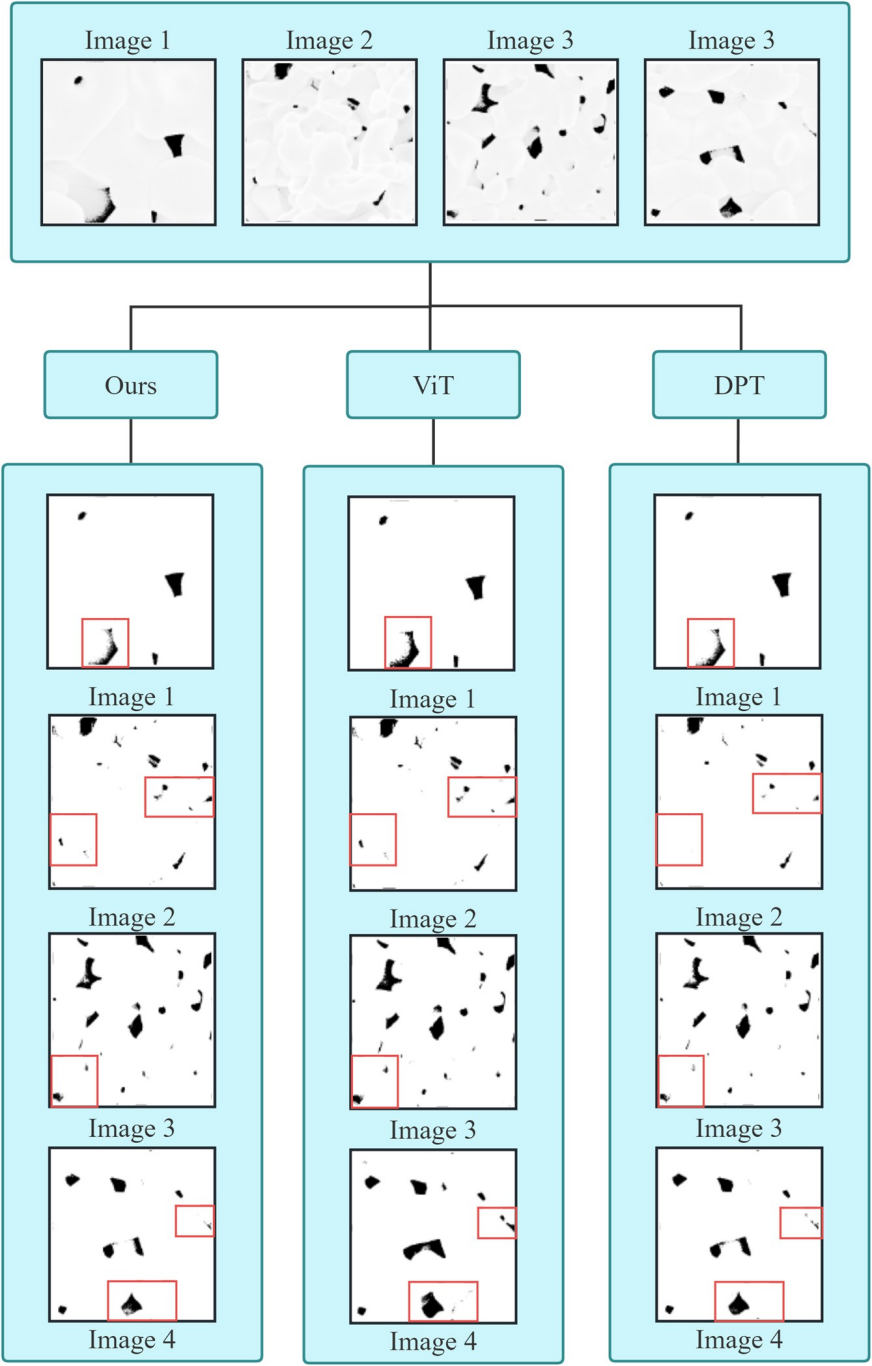
Segmentation of Al_2_O_3_ ceramic dataset with different models.

**Fig 8:**
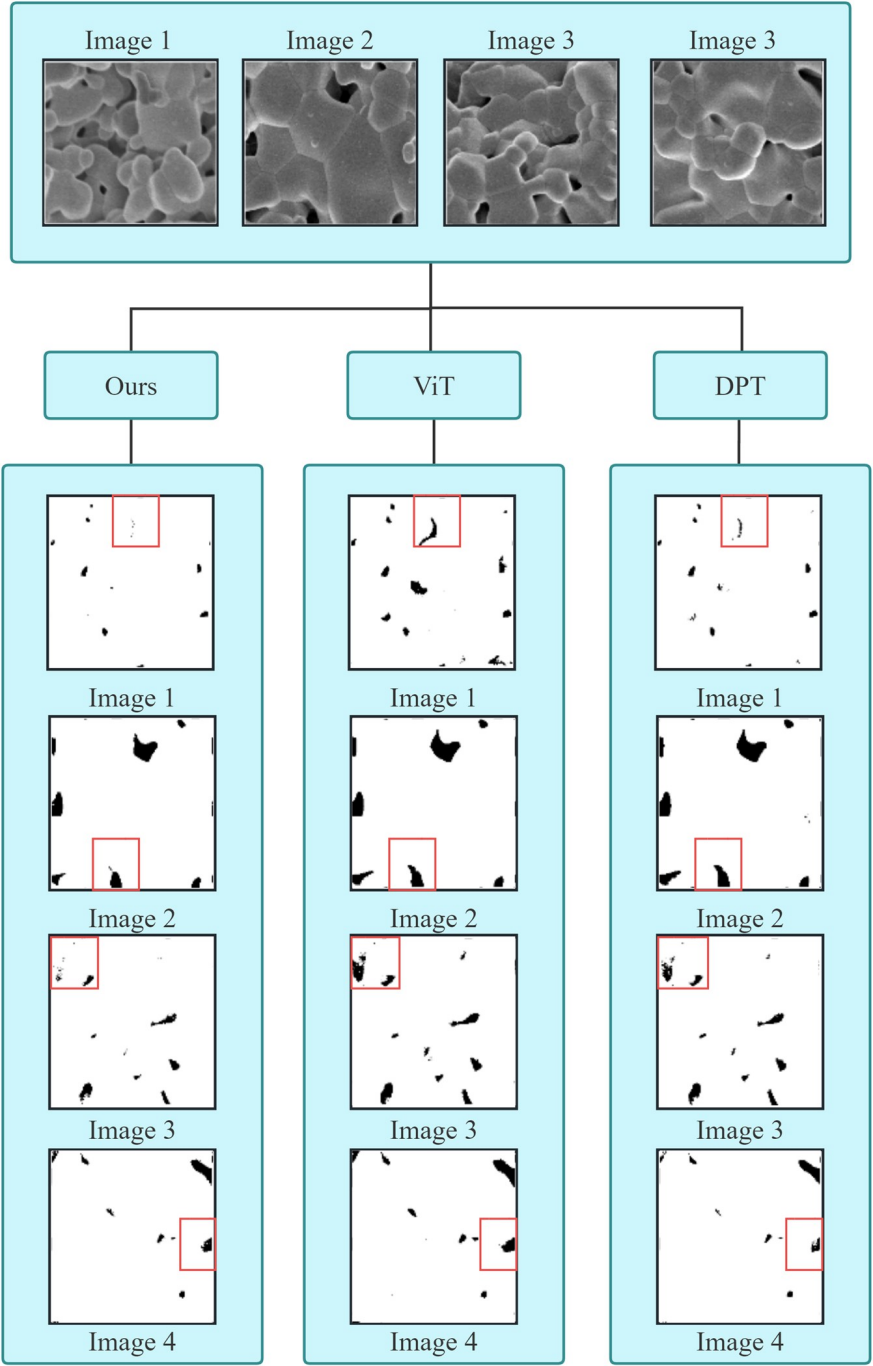
Segmentation maps of the Y_2_O_3_ ceramic dataset with different models.

We selected four images from Al_2_O_3_ and Y_2_O_3_ datasets (Figs 7 and 8), each containing varying numbers, shapes and sizes of pores, to test the performance of our best segmentation model [48]. In the Al_2_O_3_ ceramic dataset (S2 File), all three models correctly identified the pores in the images. However, there were slight differences in edge details. ViT exhibited some issues with over-segmentation in Image 1, while DPT missed some details in the lower-left corner of Image 2. PSTNet, however, correctly identified the pores within the red bounding box in Image 4, demonstrating significantly higher recognition accuracy as compared with other models. In the Y_2_O_3_ ceramic dataset (S3 File), ViT again faced over-segmentation issues in Image 1. In Image 2, PSTNet’s segmentation details within the red bounding box were notably superior to the other two methods. In the upper-right red bounding box of Image 3, due to the interference of false pores, all three models exhibited more or less recognition errors. However, PSTNet had the lowest error rate among them. The pixel percentage of undetected pores in the images is approximately 0.08%, which is negligible. These results underscore PSTNet’s effectiveness in accurately identifying and segmenting pores across different materials and scenarios.

**Fig 9:**
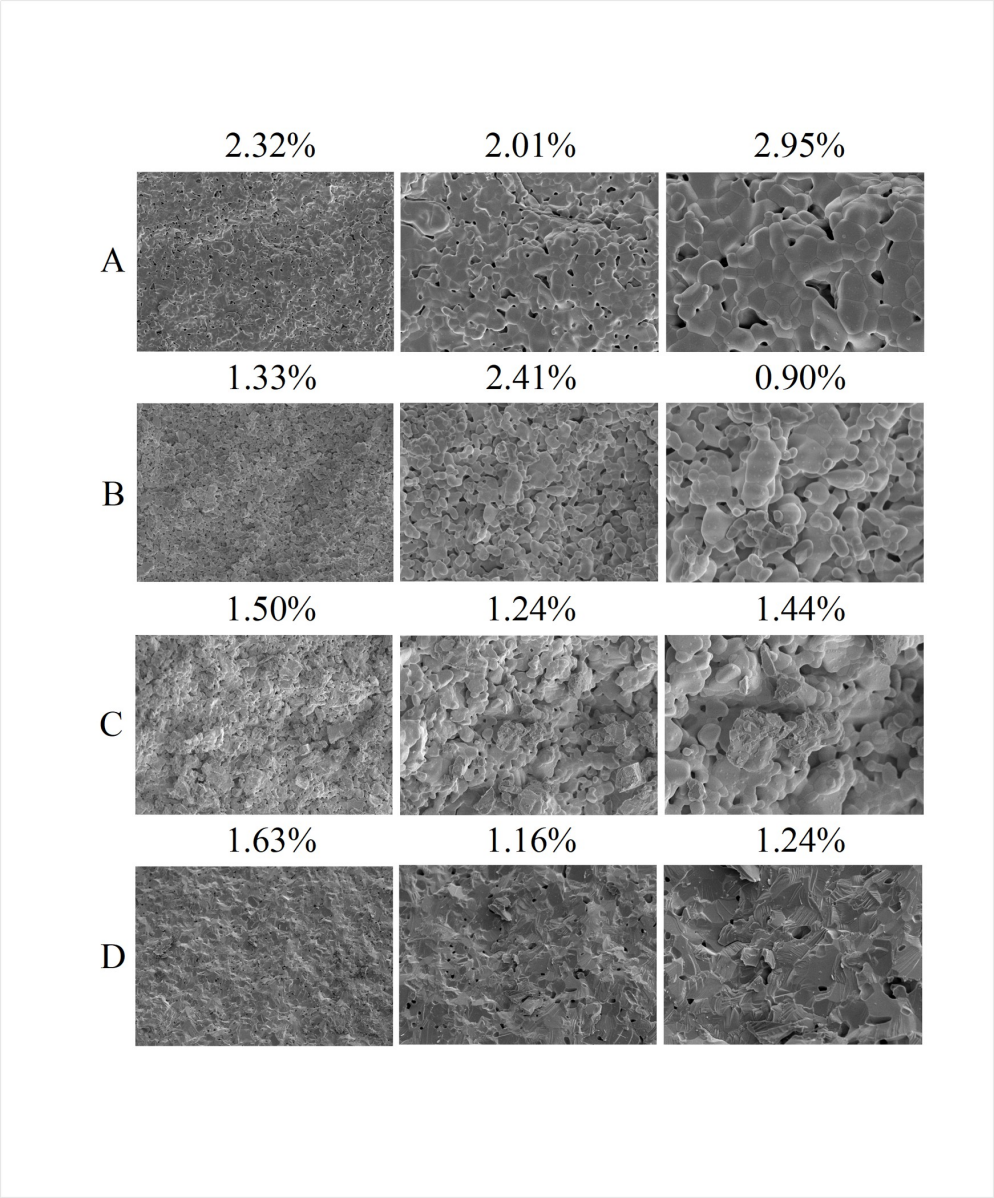
SEM images of Y_2_O_3_ ceramic.

In order to assess the discrepancy between the actual porosity and the calculated results from our developed model, we consider the measurements obtained using ImageJ software as the ground truth porosity. The relative error is employed as the evaluation metric (Fig 9 and Table 3).

**Table 3:** Porosity error of Y2O3.

Method	Model	Relative Error(%)		
		2.00k	5.00k	10.00k
Ronneberger et al. 2015 [15]	U-Net	9.61	9.82	9.75
Dosovitskiy et al. 2020 [22]	ViT	8.67	8.72	8.99
Ranftl et al. 2021 [24]	DPTNet	7.61	7.65	7.79
Wang et al. 2021 [25]	PVTNet	7.92	7.59	7.74
Chen et al. 2017 [45]	OCNet	8.63	8.87	8.61
Liu et al. 2019 [46]	ACNet	8.40	8.31	8.45
Yuan et al. 2019 [47]	Deeplab V3	8.33	8.25	8.10
The present work	PSTNet	6.84	7.12	6.83

The experiment presents SEM images of four groups of Y_2_O_3_ ceramics (labeled A-D) at different magnification levels, from left to right, 2k, 5k, and 10k. At different magnification levels, the average porosity percentage in SEM images of Y_2_O_3_ ceramics is 1.67%, whereas laser-sintered Al_2_O_3_ ceramics exhibit porosity ranging from 3.2% to 9.6% depending on the sintering conditions, highlighting Y_2_O_3_ ceramics’ clear advantages in ceramic preparation (Fig 9). In Chapter 3, Group B’s images exhibited a notable presence of pseudo-porosity, specifically in the form of pixels with intermediate values. These pixels are not actual pores but bear a striking resemblance to pores, which has an impact on the model’s assessment. Consequently, the relative error has increased.

PSTNet achieved highly accurate results on the Y_2_O_3_ dataset (Table 3). Specifically, at a magnification of 2k, this study reduced the average relative error compared to the baseline model (ViT) by 21.1% (8.67% vs. 6.84%). Compared to the suboptimal DPTNet, the present study reduced the average relative error by 10.1% (7.61% vs. 6.84%). Additionally, when predicting SEM images at different magnification levels, the predicted values exhibited minimal fluctuation, demonstrating high robustness.

**Fig 10:**
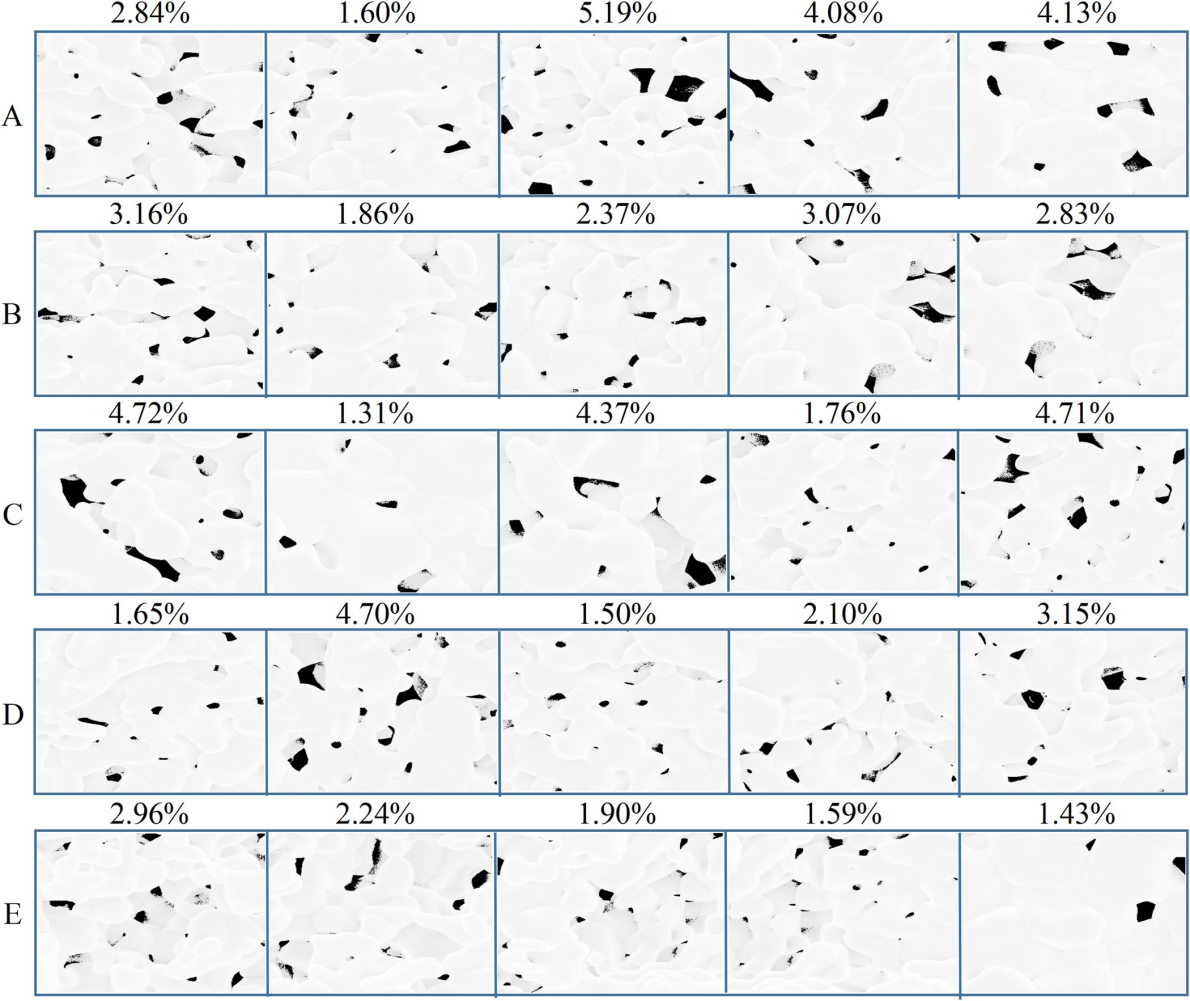
SEM images of Al_2_O_3_ ceramics.

Wang et al. [26] obtained SEM images of Al_2_O_3_ ceramics using laser-sintered aluminum strips (S2 File). We employed their processed SEM images of Al_2_O_3_ to analyze porosity (Fig 10 and Table 4).

**Table 4:** Porosity error of Al_2_O_3_.

Method	model	Average Relative Error(%)
Ronneberger et al. 2015 [15]	U-Net	9.12
Dosovitskiy et al. 2020 [22]	ViT	7.59
Ranftl et al. 2021 [24]	DPTNet	7.10
Wang et al. 2021 [25]	PVTNet	7.11
Chen et al. 2017 [45]	OCNet	8.11
Liu et al. 2019 [46]	ACNet	7.52
Yuan et al. 2019 [47]	Deeplab V3	7.44
Wang et al. 2023 [26]	Mask R–CNN	11.75
The present work	PSTNet	6.36

The experiment showcases SEM images of five groups of Al_2_O_3_ ceramics (labeled A-E) at the same magnification level (Fig 10). Since the pores in the Al_2_O_3_ SEM images have already been annotated, there was no significant error observed during prediction, resulting in an overall satisfactory performance. The average porosity rate in these images is 2.76%.

PSTNet demonstrated highly accurate results on the Al_2_O_3_ dataset. Specifically, our study achieved a significant reduction in average relative error by 16.21% compared to the baseline model (ViT) (7.59% vs. 6.36%). Additionally, our approach outperformed the next best model, DPTNet, with a 10.42% decrease in average relative error (7.10% vs. 6.36%). In contrast, Wang et al. [26] categorized images into multiple groups based on varying laser sintering powers (7.2w-10.8w) and reported relative errors ranging from 15.9% to 8.1% for the test set, with an average relative error of 11.75%. Clearly, in terms of accuracy, our study demonstrates superior performance.

The mechanical properties of ceramic materials are closely correlated with their microstructure. For instance, flexural strength typically increases with a decrease in porosity. Moreover, under a constant porosity level, strength tends to increase with a reduction in grain size. Additionally, the chemical composition of ceramic materials plays a critical role in their insulation and high-temperature resistance properties. Materials such as Al_2_O_3_, Y_2_O_3_, and Si3N4 exhibit high insulation and high-temperature resistance, maintaining stability at elevated temperatures and resisting chemical reactions.

Experimental results demonstrate that the custom-made Y_2_O_3_ ceramic exhibits a lower average porosity rate of only 1.67% as compared with Al_2_O_3_ ceramics. Consequently, this results in higher materials with flexural strength. Furthermore, the microstructure of Y_2_O_3_ ceramics may exhibit smaller grain sizes, contributing to increased strength.

Therefore, optimizing the performance of ceramic materials can be achieved by adjusting fabrication processes and controlling microstructural features. Measures such as reducing porosity and regulating grain size can enhance the strength and toughness of ceramic materials. Additionally, incorporating Y_2_O_3_ ceramics as the main component with residual doping elements such as ZrO_2_, MgO, or CeO_2_ can significantly enhance high-temperature resistance, meeting the requirements of various application domains.

### Ablation experiment

We conducted ablation studies on the Y_2_O_3_ dataset, providing quantitative results for the PVT backbone (Table 5), ResNet Fusion, Linear SRA Fusion, and the loss function. Initially, we used the ViT model as the baseline and applied ResNet Fusion on top of ViT. Subsequently, we replaced the ViT backbone with the PVT backbone while retaining ResNet Fusion for the second set of experiments. Building upon the second set of experiments, the third set replaced ResNet Fusion with Linear SRA Fusion. Finally, based on the third set of experiments, the fourth set incorporated SSIM Loss and Smooth Loss on top of the SPCE loss.

**Table 5:** Ablation study on Y_2_O_3_ dataset.

Components				Y_2_O_3_	
PVT backbone	ResNet Fusion	Linear SRA Fusion	SPCE loss, SSIM loss and Smooth loss	Pix Acc[%]	mIoU[%]
	√			76.01	51.49
√	√			80.46	58.34
√		√		84.97	66.15
√		√	√	86.52	69.10

Results are presented in bold to highlight the best-performing configurations. When replacing the ViT backbone with the PVT backbone, Pix Acc and mIoU metrics increased by 4.45% and 6.85%, respectively. This indicates that the PVT backbone yields higher semantic segmentation accuracy compared to the ViT backbone while maintaining faster computational speed. Subsequently, replacing the ResNet Fusion module with Linear SRA Fusion led to a noticeable improvement in Pix Acc and mIoU metrics, demonstrating the significant enhancement in predictive accuracy with the integration of attention mechanisms, albeit with an increase in parameter size. Finally, with the introduction of SPCE loss, SSIM Loss and Smooth Loss, Pix Acc and mIoU metrics reached 86.52% and 69.10%, respectively. This signifies that incorporating SSIM Loss and Smooth Loss indeed contributes to enhancing model performance, pushing the model towards its optimal state.

## Conclusions

In this work, we propose a novel semantic segmentation method, PSTNet, which combines Transformer and FPN architectures. To analyze pore distribution in ceramic SEM images, we prepared Y_2_O_3_ ceramic samples using high-temperature hot pressing sintering and constructed a Y_2_O_3_ ceramic dataset through preprocessing. PSTNet was trained and tested on both public Al_2_O_3_ and custom Y_2_O_3_ ceramic datasets to demonstrate its effectiveness.

In the encoding phase, PSTNet integrates a PVT backbone network to extract robust feature representations, employing progressive upsampling and multi-scale feature fusion to effectively retain both shallow and deep semantic information, thus enhancing modeling capabilities for complex scenes and long sequences. In the decoding phase, an enhanced MHA module replaces traditional residual fusion modules, significantly improving feature fusion efficiency and enabling precise segmentation of ceramic particles and pores in SEM images to calculate pore percentages.

PSTNet utilizes SPCE loss, Smooth L1 loss, and SSIM loss as a joint loss function to optimize model performance, resulting in superior training and validation losses compared to peer models. These enhancements significantly improve the model’s accuracy in terms of Pix ACC and mIoU.

However, the applicability of PSTNet is constrained by limited data volume, posing challenges to improving model generalization performance. Single semantic segmentation networks also demonstrate limited effectiveness in tackling more complex semantic segmentation scenarios. To address these issues, future research will introduce cue words and cascade multiple networks, integrating the concept of multimodal learning into the field of material semantic segmentation. Additionally, to significantly enhance model generalization, we plan to diversify data types and expand dataset volume by collecting and preprocessing various data types such as low-carbon steel and diamonds, aimed at tackling increasingly complex semantic segmentation scenarios in materials science.

## Supporting information

S1 File (ZIP)

S2 File (ZIP)

S3 File (ZIP)
