# Supplementary figures and images for: Deep-learning-based pyramid-transformer for localized porosity analysis of hot-press sintered ceramic paste (part 1 of 3)
Source: PLoS One. 2024 Sep 4;19(9):e0306385. doi: 10.1371/journal.pone.0306385 (PMC11373816; doi:10.1371/journal.pone.0306385)

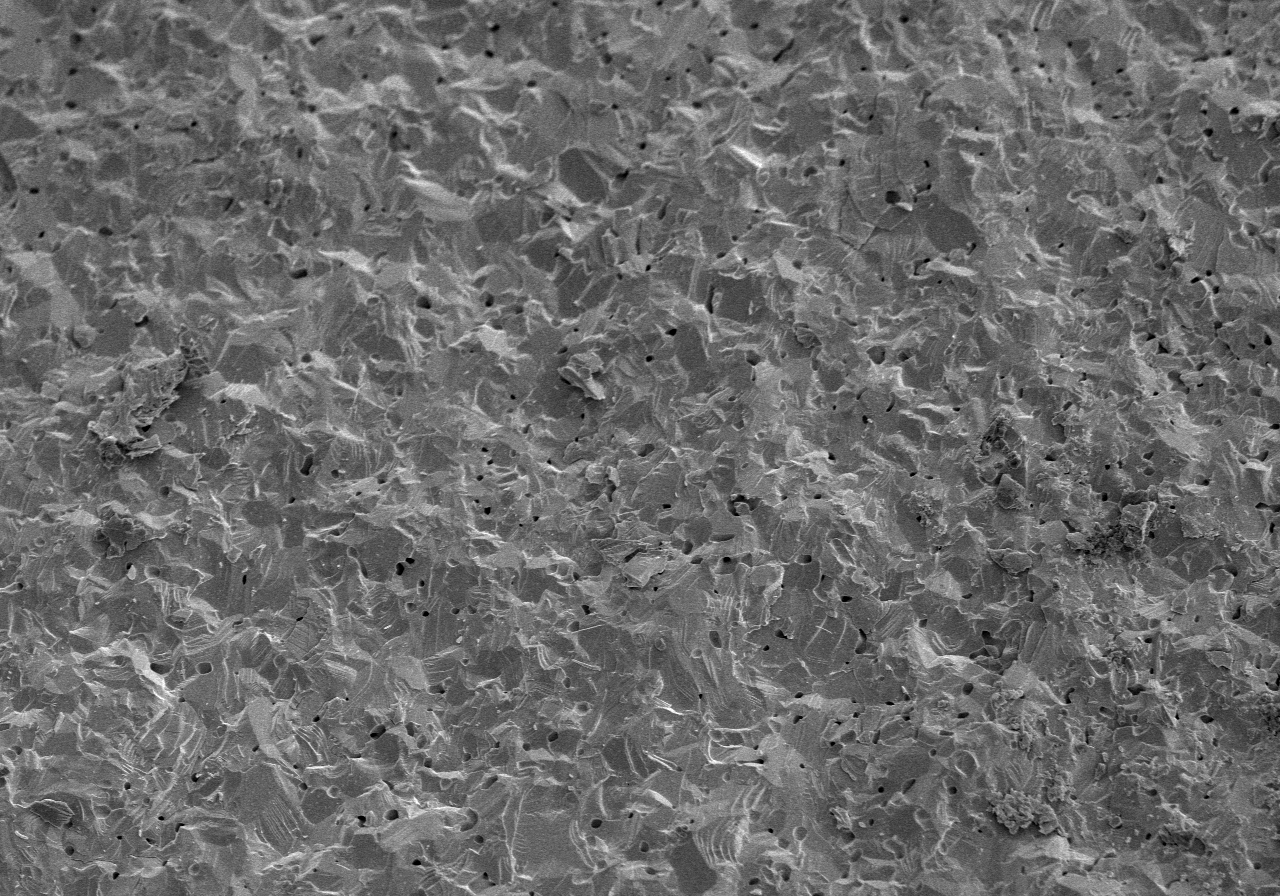

Supplement: S1 File — (ZIP) [file pone.0306385.s001.zip › S1/20-1.tif]

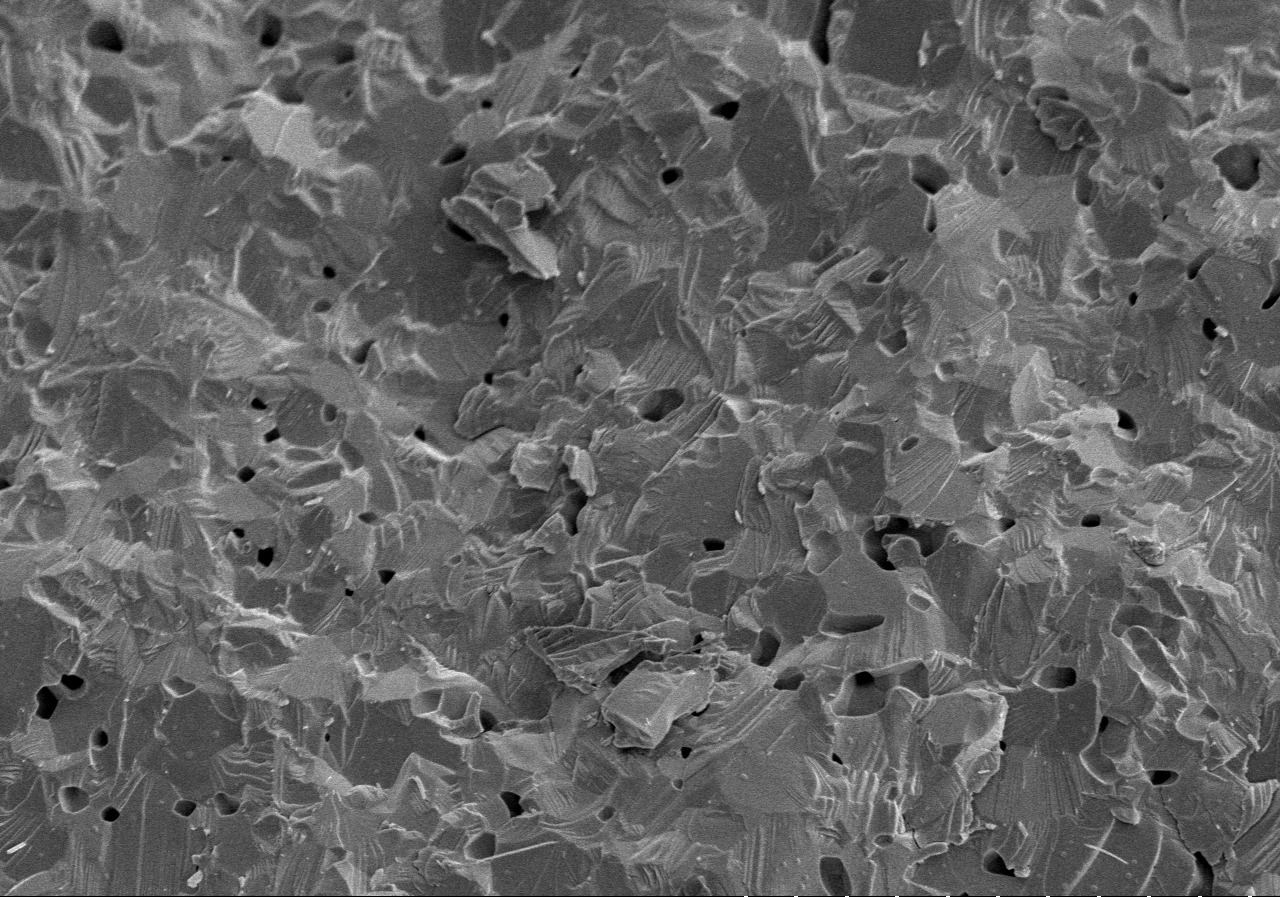

Supplement: S1 File — (ZIP) [file pone.0306385.s001.zip › S1/20-2.tif]

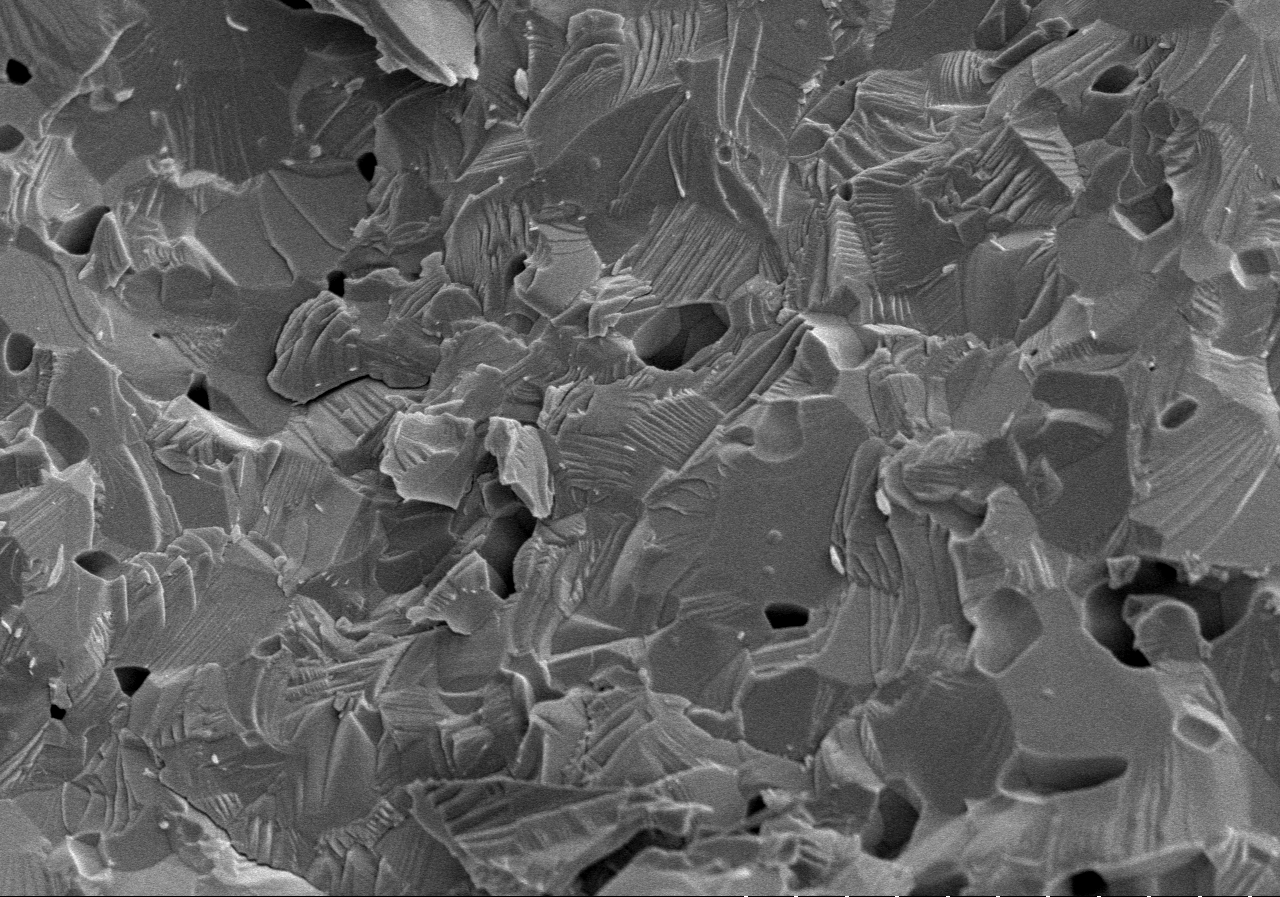

Supplement: S1 File — (ZIP) [file pone.0306385.s001.zip › S1/20-3.tif]

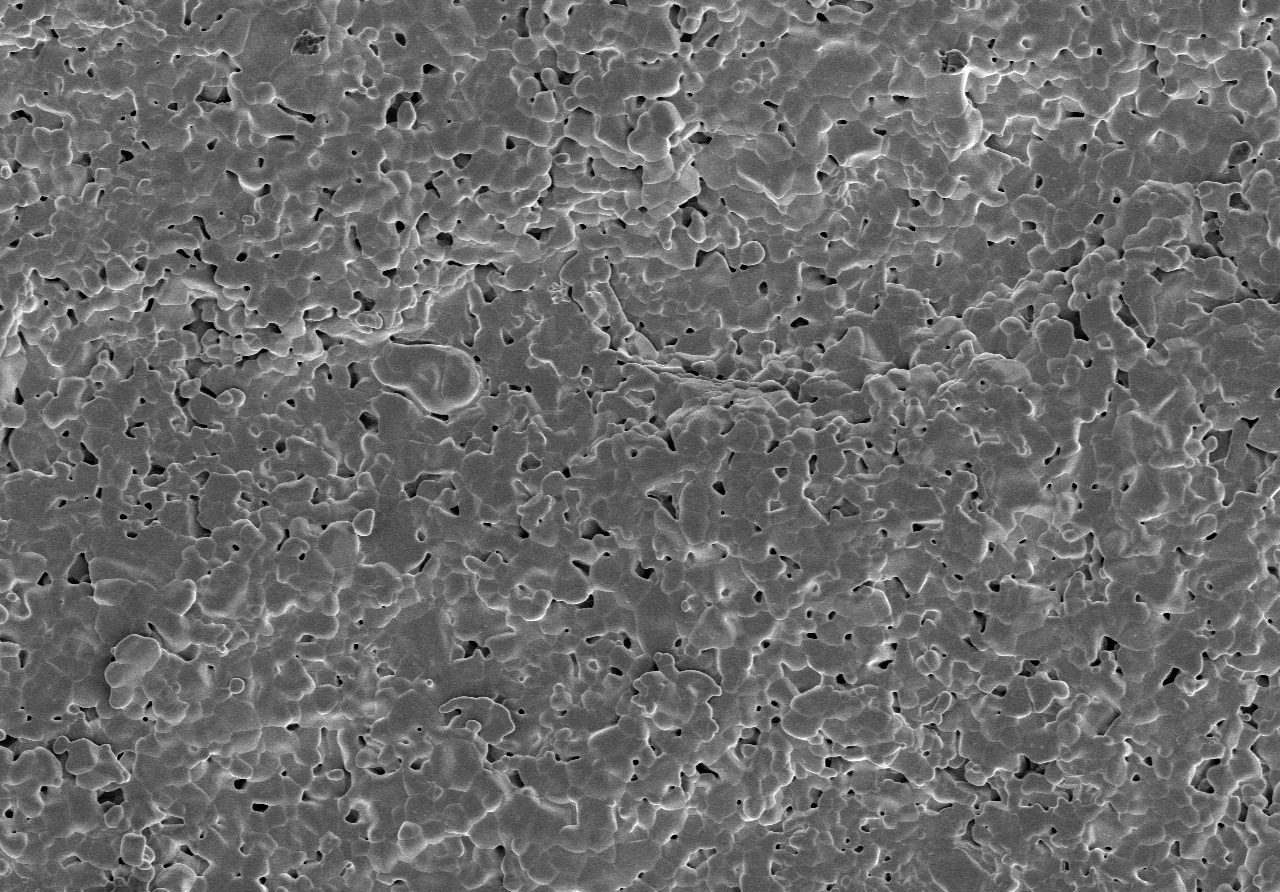

Supplement: S1 File — (ZIP) [file pone.0306385.s001.zip › S1/3-1.tif]

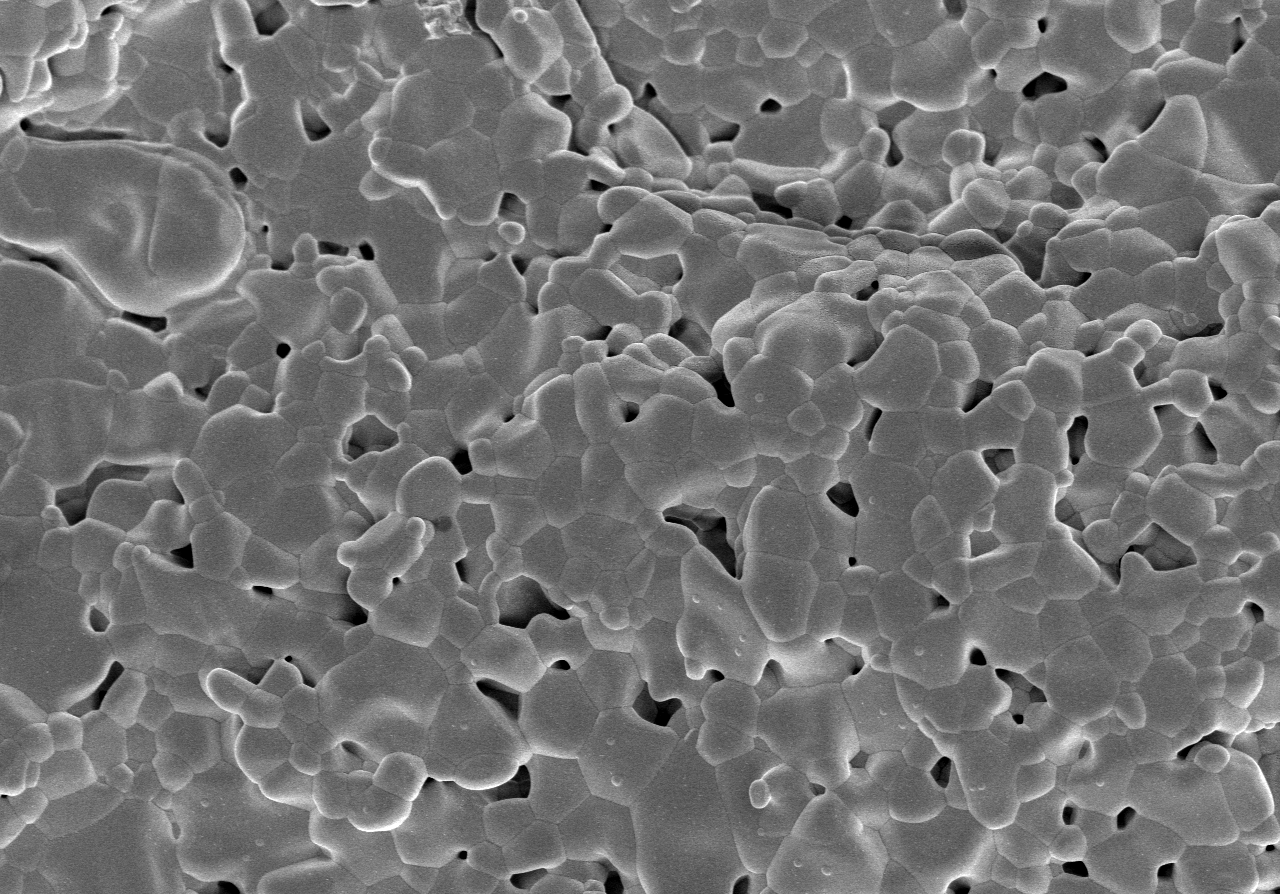

Supplement: S1 File — (ZIP) [file pone.0306385.s001.zip › S1/3-2.tif]

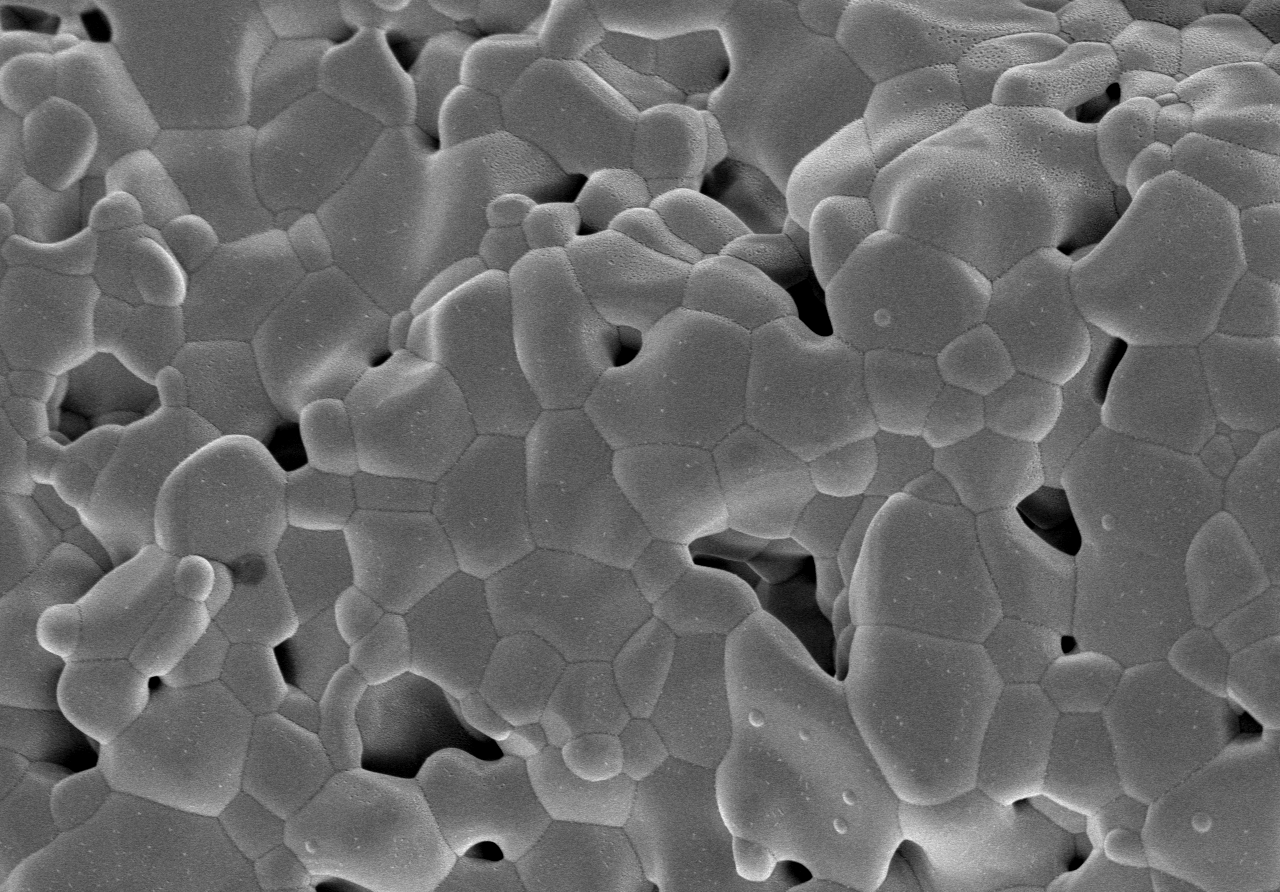

Supplement: S1 File — (ZIP) [file pone.0306385.s001.zip › S1/3-3.tif]

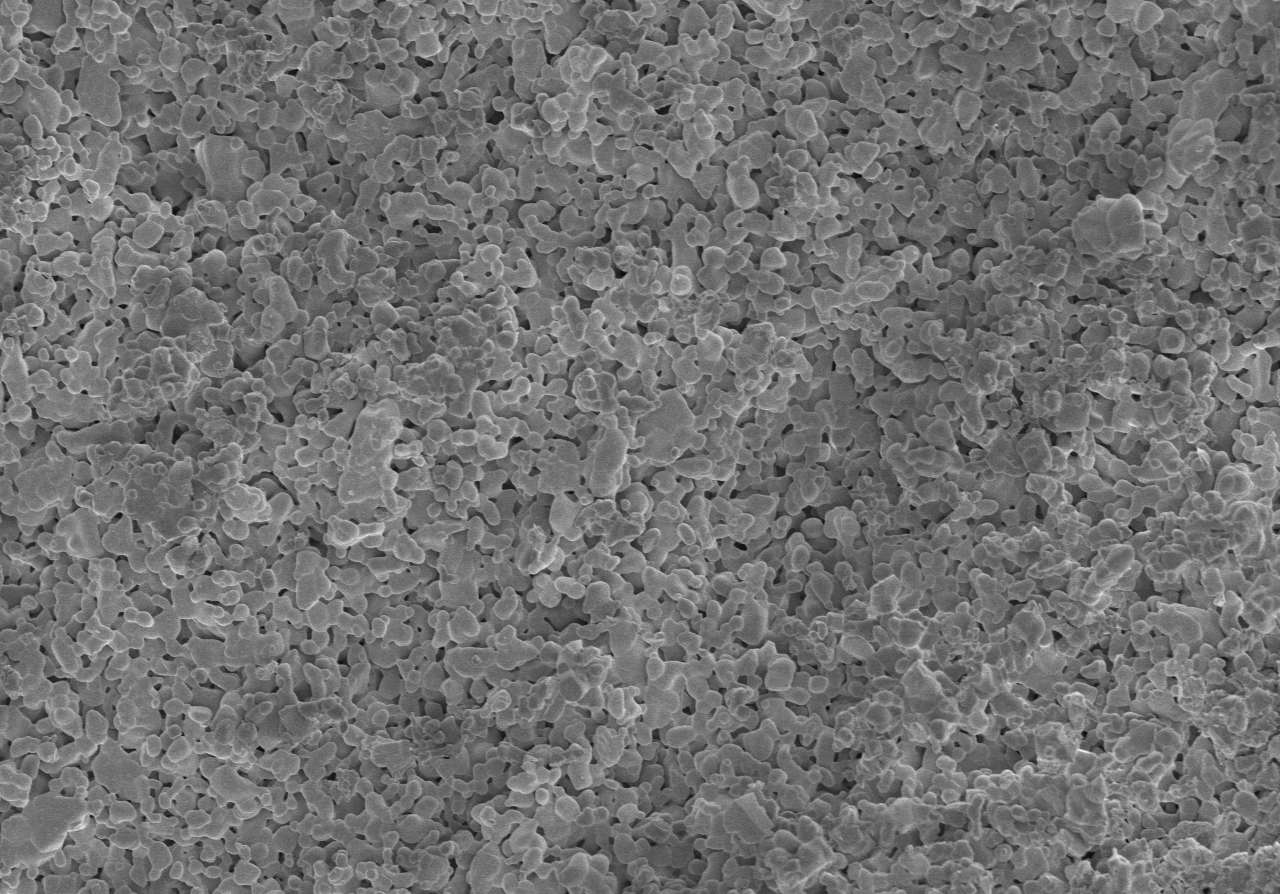

Supplement: S1 File — (ZIP) [file pone.0306385.s001.zip › S1/30-1.tif]

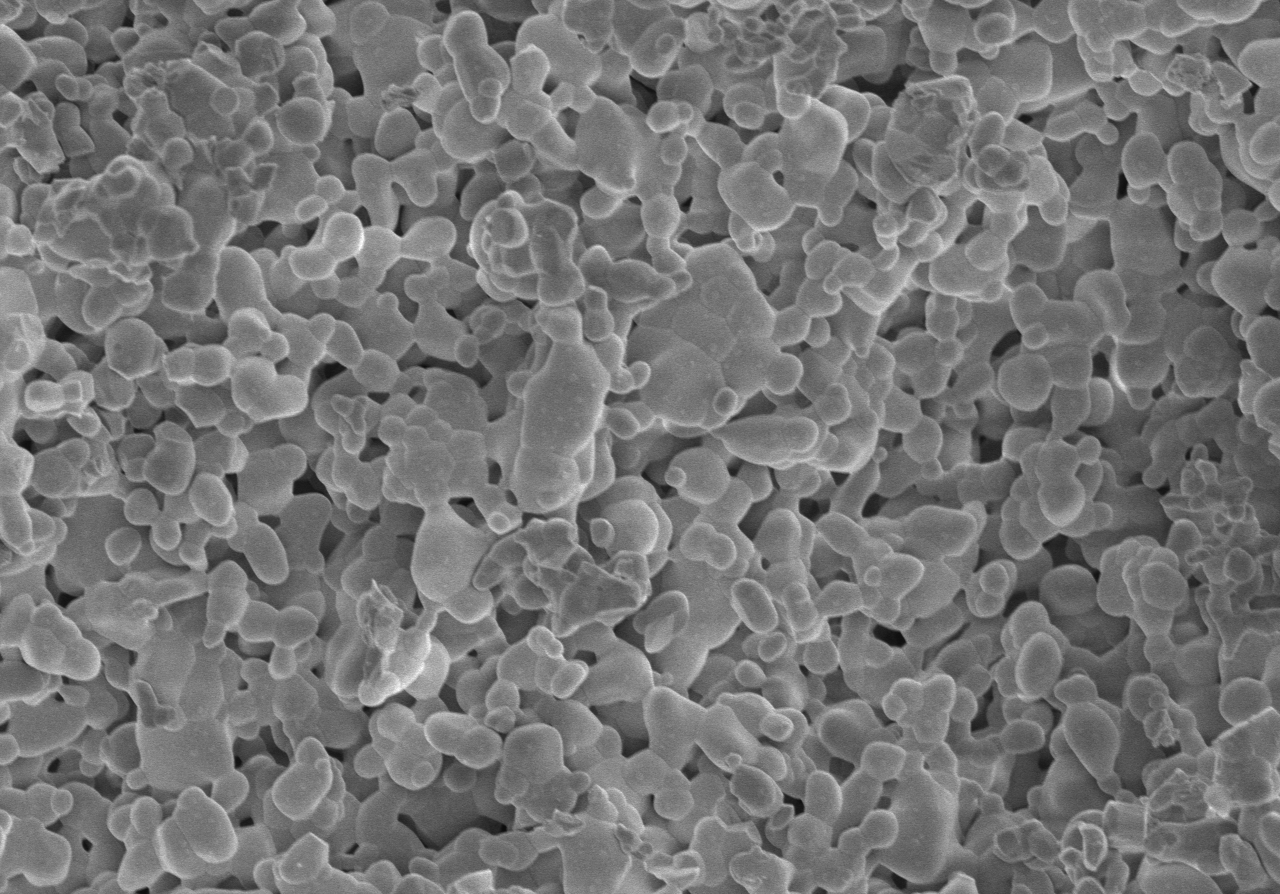

Supplement: S1 File — (ZIP) [file pone.0306385.s001.zip › S1/30-2.tif]

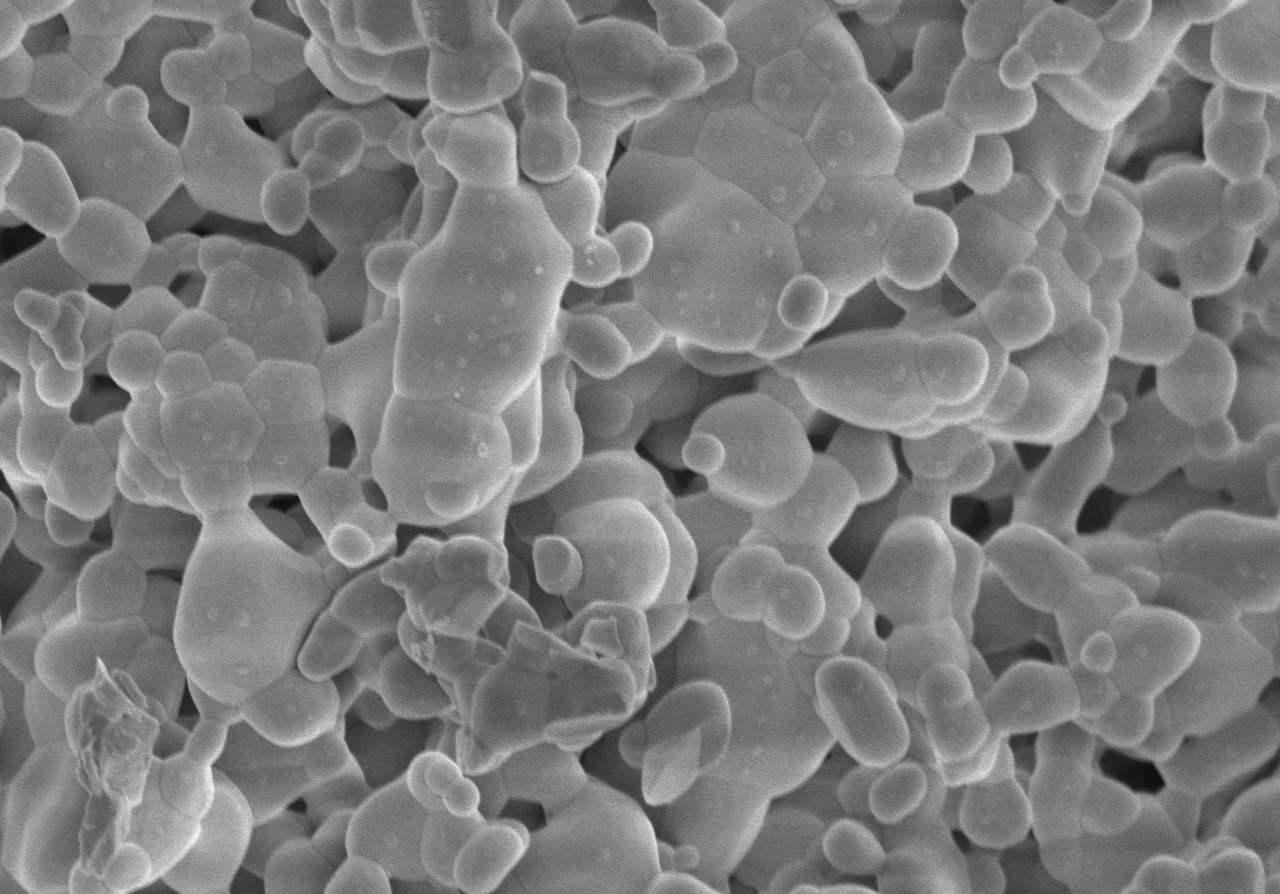

Supplement: S1 File — (ZIP) [file pone.0306385.s001.zip › S1/30-3.tif]

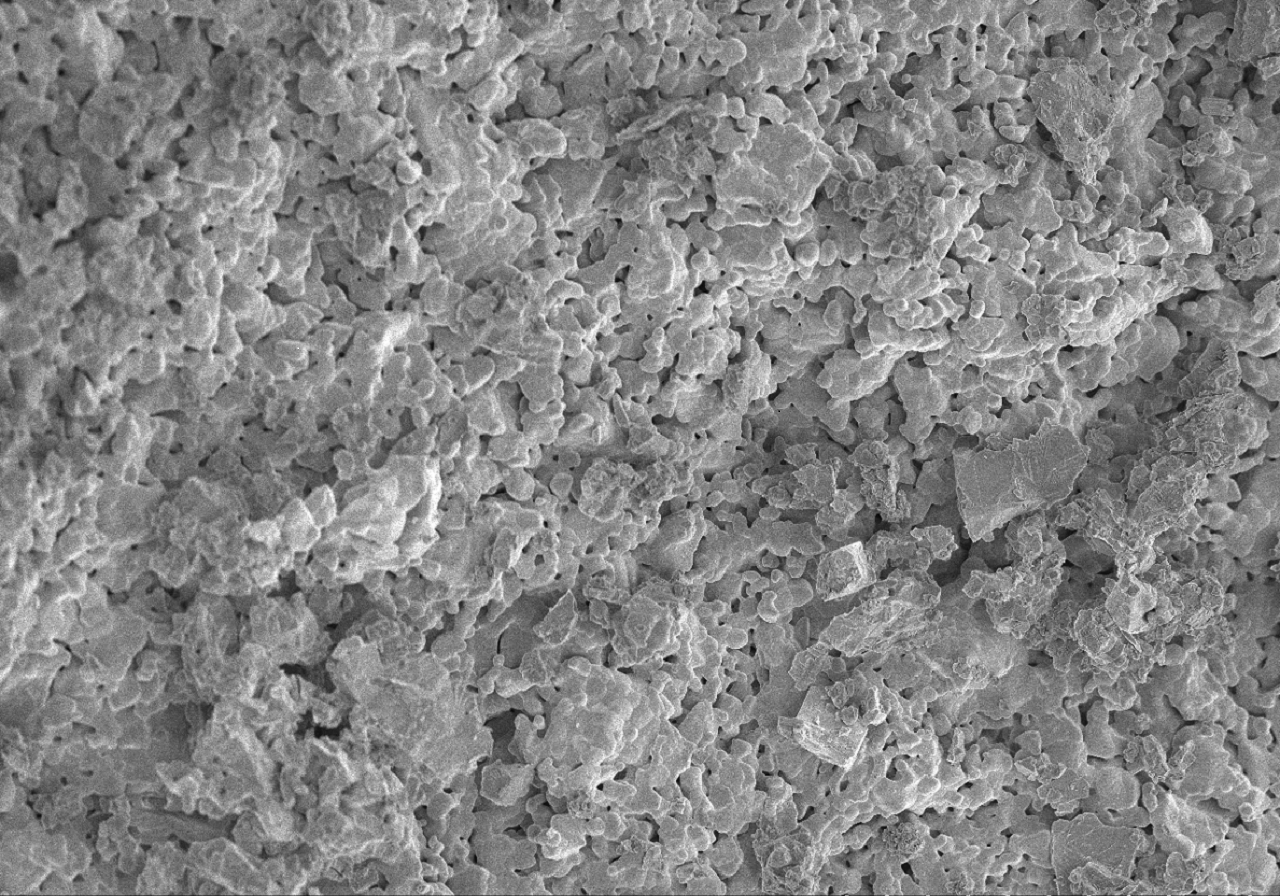

Supplement: S1 File — (ZIP) [file pone.0306385.s001.zip › S1/7-1.tif]

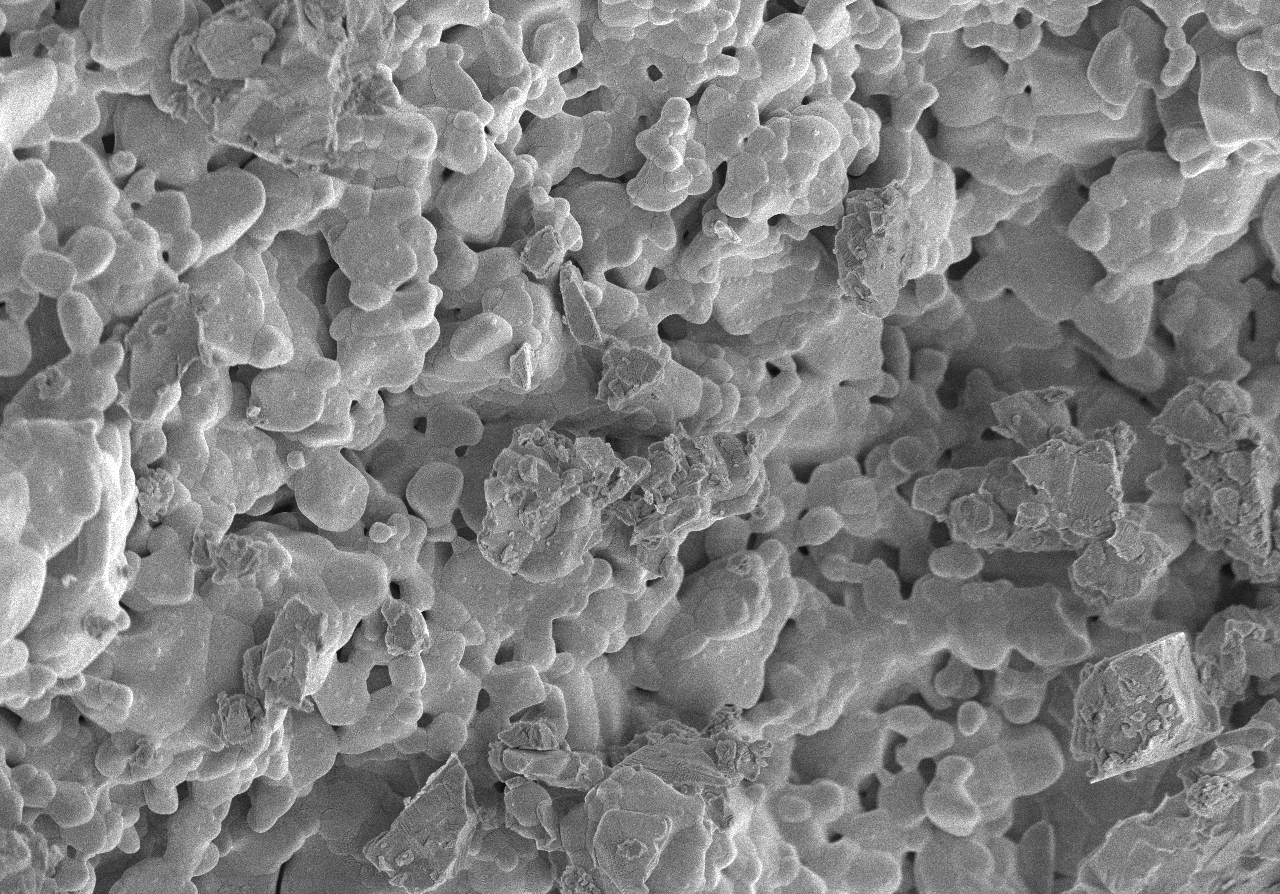

Supplement: S1 File — (ZIP) [file pone.0306385.s001.zip › S1/7-2.tif]

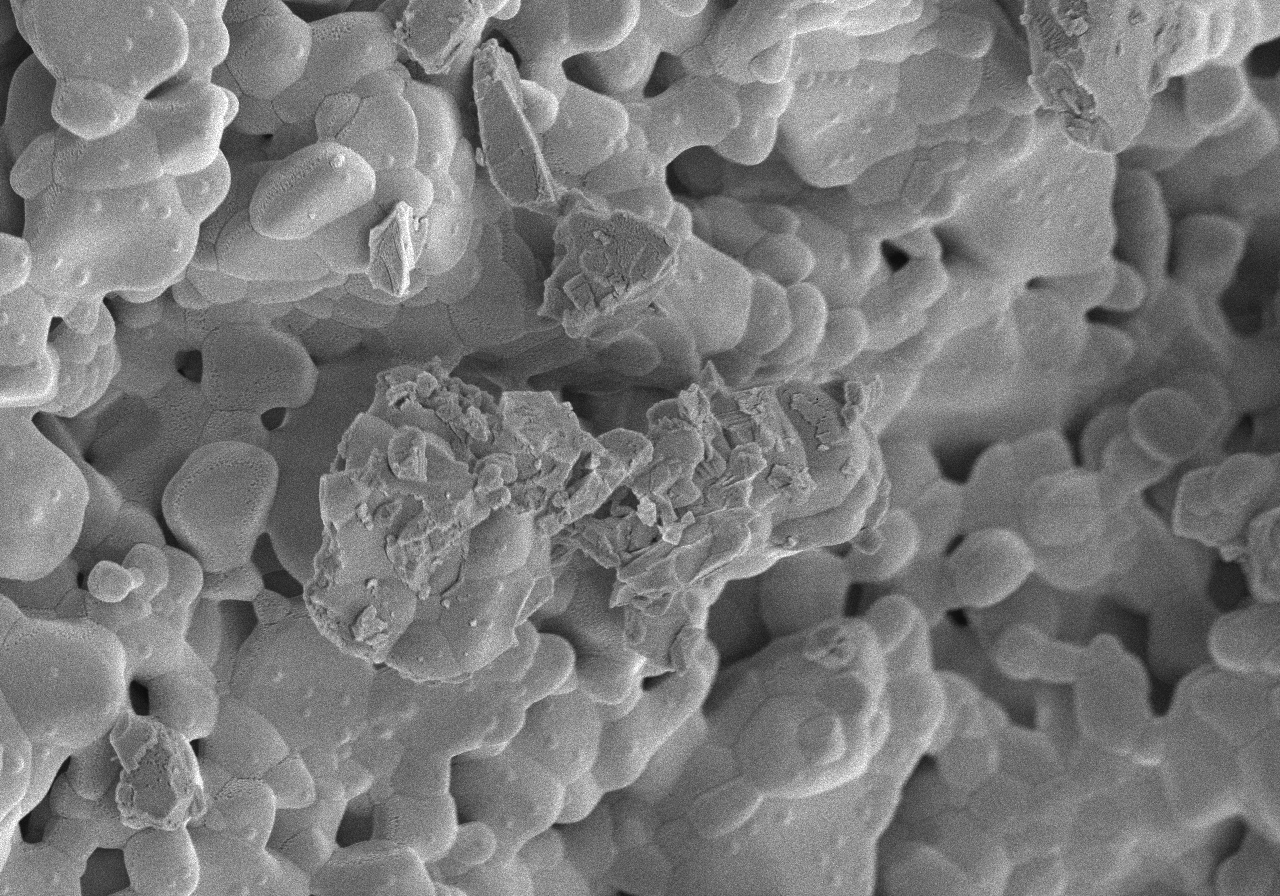

Supplement: S1 File — (ZIP) [file pone.0306385.s001.zip › S1/7-3.tif]

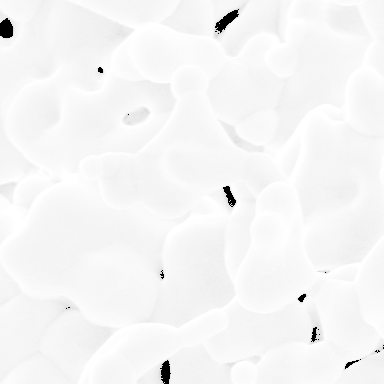

Supplement: S2 File — (ZIP) [file pone.0306385.s002.zip › S2/train/15_1-03.tif_10.png]

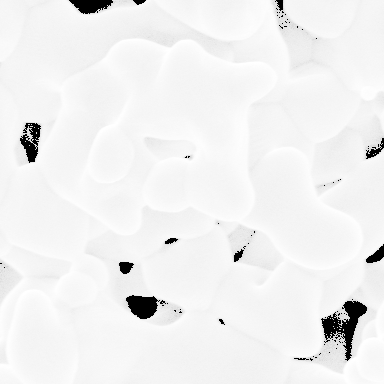

Supplement: S2 File — (ZIP) [file pone.0306385.s002.zip › S2/train/15_1-03.tif_2.png]

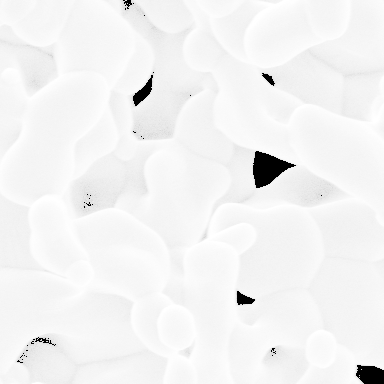

Supplement: S2 File — (ZIP) [file pone.0306385.s002.zip › S2/train/15_1-03.tif_4.png]

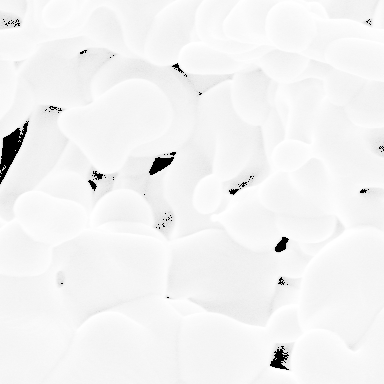

Supplement: S2 File — (ZIP) [file pone.0306385.s002.zip › S2/train/15_1-03.tif_5.png]

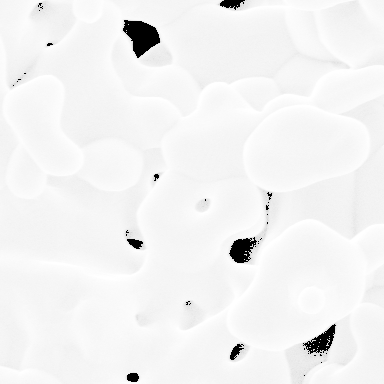

Supplement: S2 File — (ZIP) [file pone.0306385.s002.zip › S2/train/15_1-03.tif_6.png]

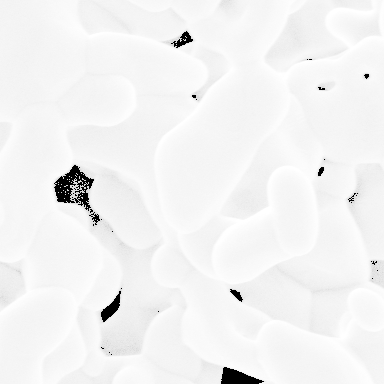

Supplement: S2 File — (ZIP) [file pone.0306385.s002.zip › S2/train/15_1-03.tif_7.png]

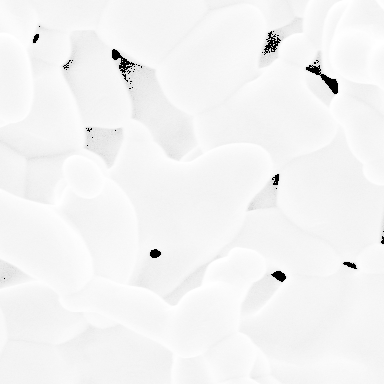

Supplement: S2 File — (ZIP) [file pone.0306385.s002.zip › S2/train/15_1-03.tif_8.png]

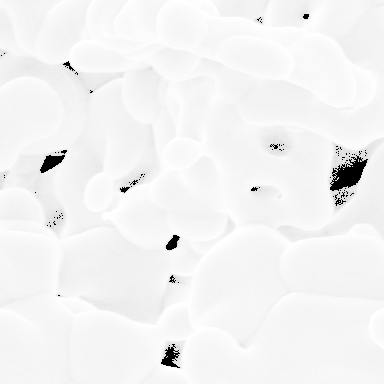

Supplement: S2 File — (ZIP) [file pone.0306385.s002.zip › S2/train/15_1-03.tif_9.png]

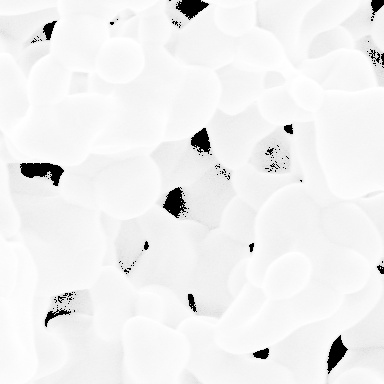

Supplement: S2 File — (ZIP) [file pone.0306385.s002.zip › S2/train/15_1-06.tif_10.png]

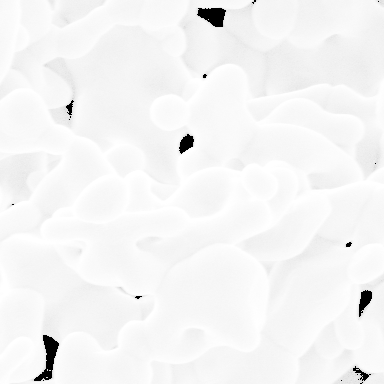

Supplement: S2 File — (ZIP) [file pone.0306385.s002.zip › S2/train/15_1-06.tif_2.png]

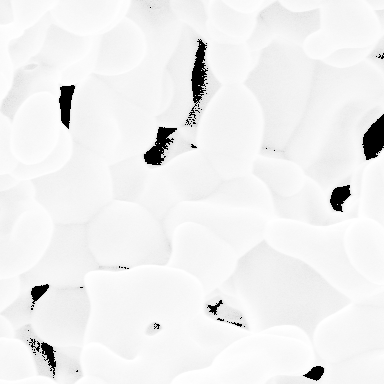

Supplement: S2 File — (ZIP) [file pone.0306385.s002.zip › S2/train/15_1-06.tif_3.png]

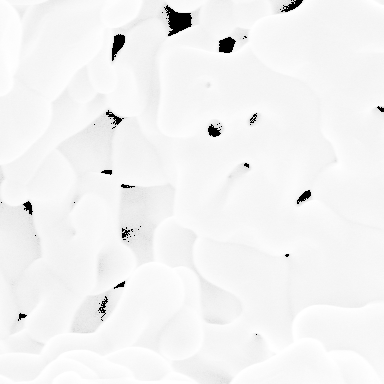

Supplement: S2 File — (ZIP) [file pone.0306385.s002.zip › S2/train/15_1-06.tif_4.png]

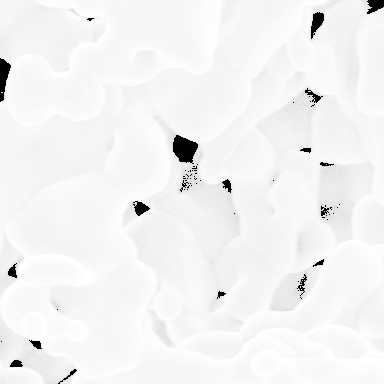

Supplement: S2 File — (ZIP) [file pone.0306385.s002.zip › S2/train/15_1-06.tif_7.png]

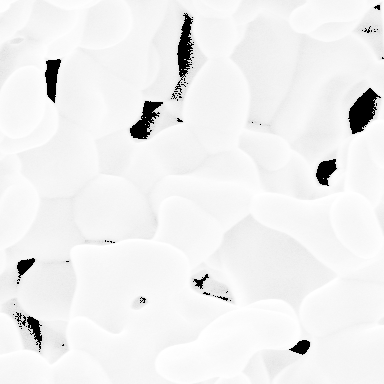

Supplement: S2 File — (ZIP) [file pone.0306385.s002.zip › S2/train/15_1-06.tif_9.png]

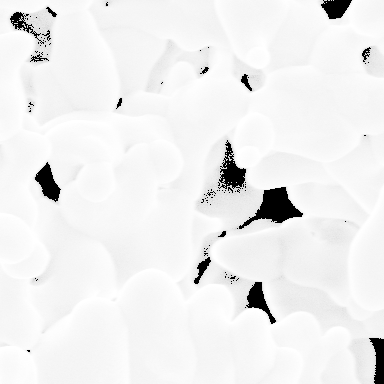

Supplement: S2 File — (ZIP) [file pone.0306385.s002.zip › S2/train/15_1-07.tif_1.png]

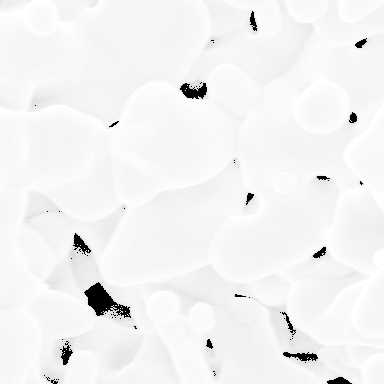

Supplement: S2 File — (ZIP) [file pone.0306385.s002.zip › S2/train/15_1-07.tif_10.png]

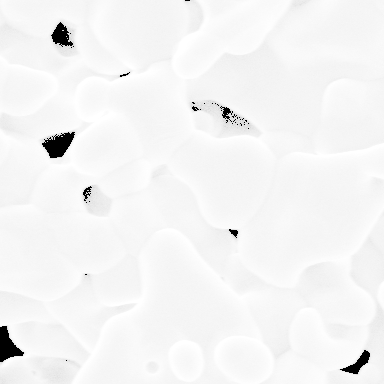

Supplement: S2 File — (ZIP) [file pone.0306385.s002.zip › S2/train/15_1-07.tif_2.png]

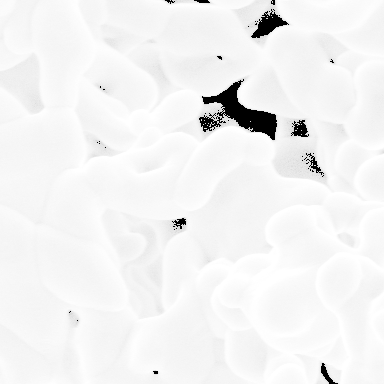

Supplement: S2 File — (ZIP) [file pone.0306385.s002.zip › S2/train/15_1-07.tif_6.png]

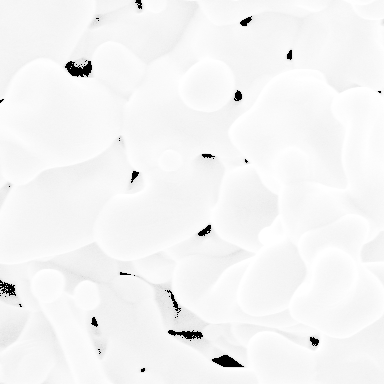

Supplement: S2 File — (ZIP) [file pone.0306385.s002.zip › S2/train/15_1-07.tif_7.png]

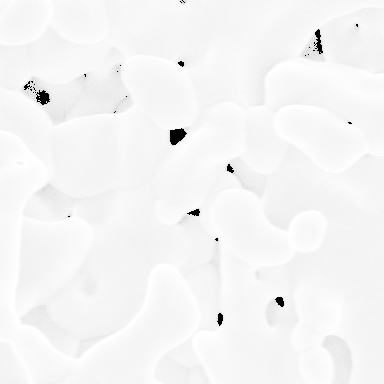

Supplement: S2 File — (ZIP) [file pone.0306385.s002.zip › S2/train/15_1-07.tif_8.png]

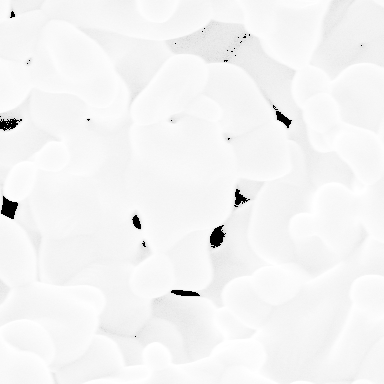

Supplement: S2 File — (ZIP) [file pone.0306385.s002.zip › S2/train/15_1-07.tif_9.png]

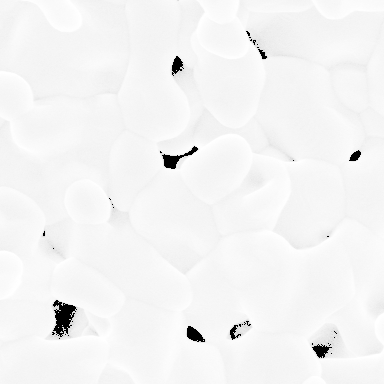

Supplement: S2 File — (ZIP) [file pone.0306385.s002.zip › S2/train/15_1-08.tif_1.png]

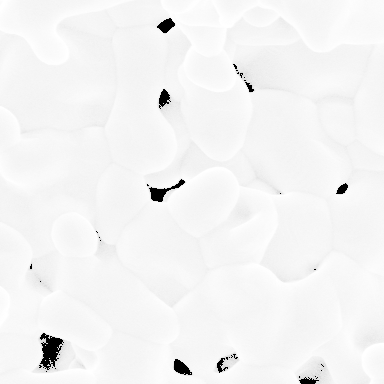

Supplement: S2 File — (ZIP) [file pone.0306385.s002.zip › S2/train/15_1-08.tif_10.png]

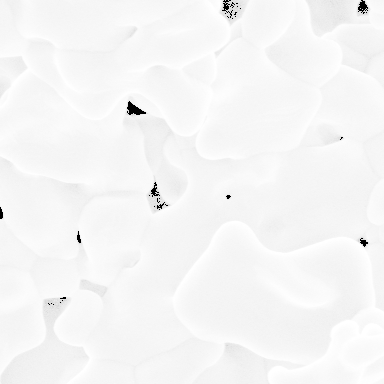

Supplement: S2 File — (ZIP) [file pone.0306385.s002.zip › S2/train/15_1-08.tif_2.png]

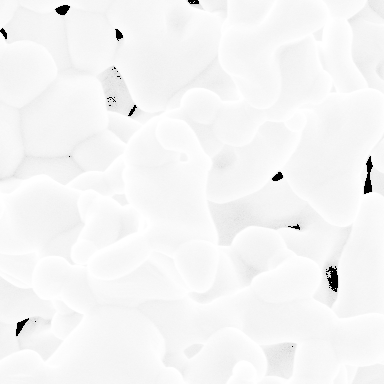

Supplement: S2 File — (ZIP) [file pone.0306385.s002.zip › S2/train/15_1-08.tif_3.png]

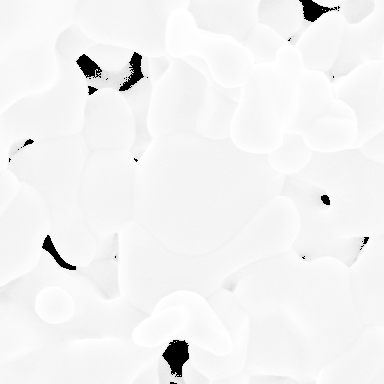

Supplement: S2 File — (ZIP) [file pone.0306385.s002.zip › S2/train/15_1-08.tif_4.png]

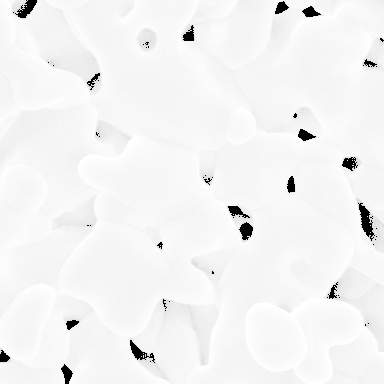

Supplement: S2 File — (ZIP) [file pone.0306385.s002.zip › S2/train/15_1-08.tif_8.png]

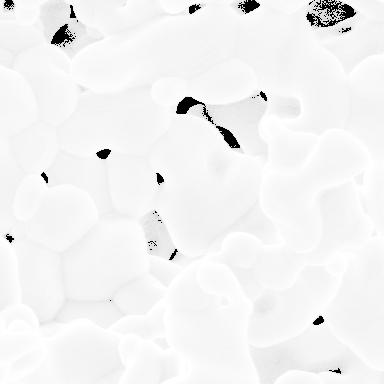

Supplement: S2 File — (ZIP) [file pone.0306385.s002.zip › S2/train/15_1-08.tif_9.png]

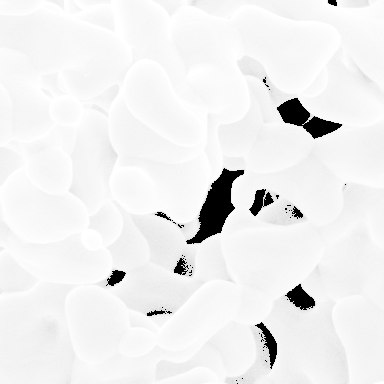

Supplement: S2 File — (ZIP) [file pone.0306385.s002.zip › S2/train/15_1-09.tif_1.png]

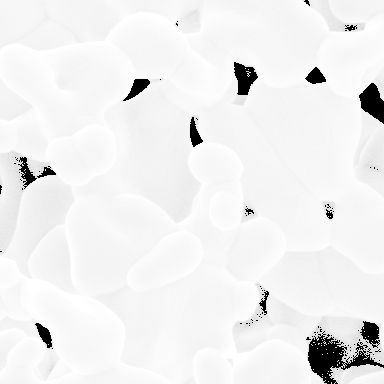

Supplement: S2 File — (ZIP) [file pone.0306385.s002.zip › S2/train/15_1-09.tif_10.png]

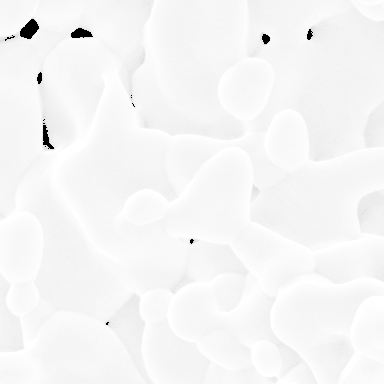

Supplement: S2 File — (ZIP) [file pone.0306385.s002.zip › S2/train/15_1-09.tif_2.png]

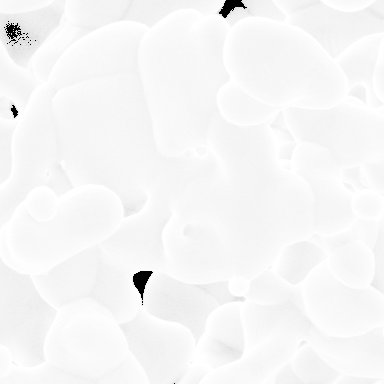

Supplement: S2 File — (ZIP) [file pone.0306385.s002.zip › S2/train/15_1-09.tif_3.png]

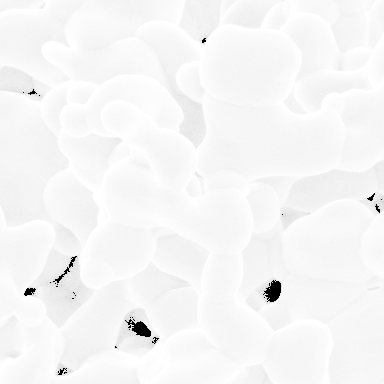

Supplement: S2 File — (ZIP) [file pone.0306385.s002.zip › S2/train/15_1-09.tif_4.png]

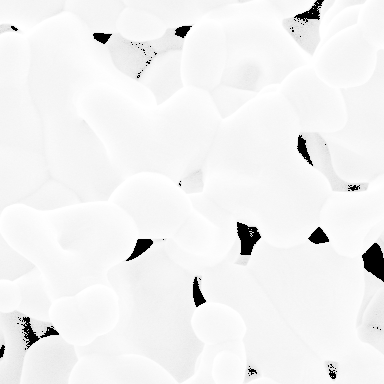

Supplement: S2 File — (ZIP) [file pone.0306385.s002.zip › S2/train/15_1-09.tif_5.png]

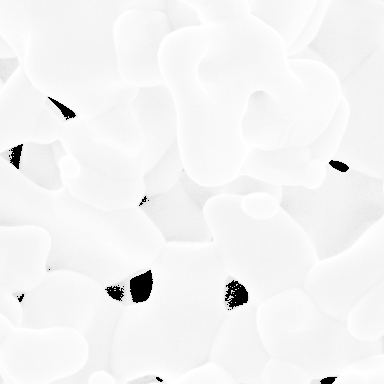

Supplement: S2 File — (ZIP) [file pone.0306385.s002.zip › S2/train/15_1-09.tif_6.png]

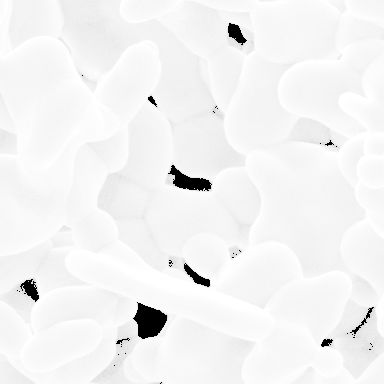

Supplement: S2 File — (ZIP) [file pone.0306385.s002.zip › S2/train/15_1-09.tif_7.png]

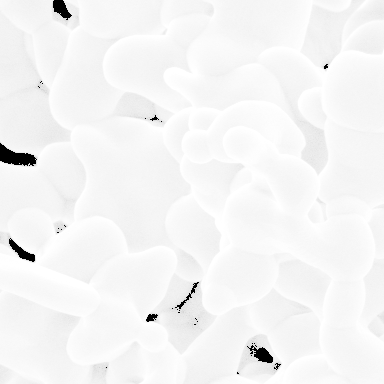

Supplement: S2 File — (ZIP) [file pone.0306385.s002.zip › S2/train/15_1-09.tif_9.png]

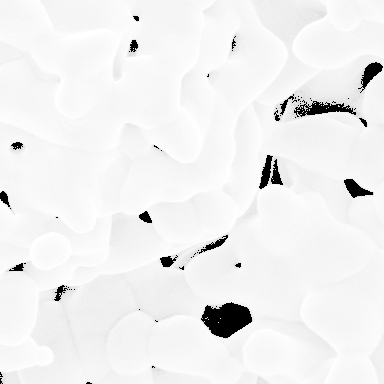

Supplement: S2 File — (ZIP) [file pone.0306385.s002.zip › S2/train/15_1-10.tif_1.png]

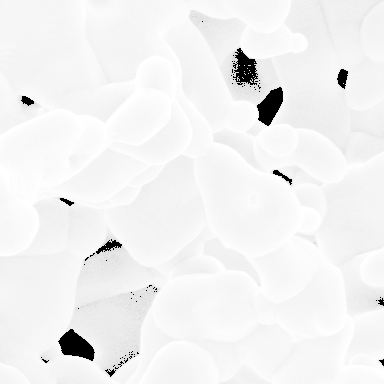

Supplement: S2 File — (ZIP) [file pone.0306385.s002.zip › S2/train/15_1-10.tif_10.png]

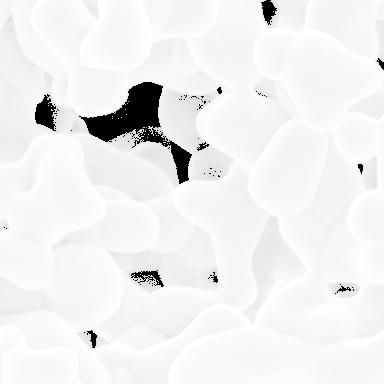

Supplement: S2 File — (ZIP) [file pone.0306385.s002.zip › S2/train/15_1-10.tif_3.png]

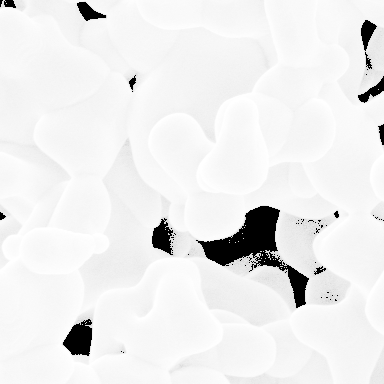

Supplement: S2 File — (ZIP) [file pone.0306385.s002.zip › S2/train/15_1-10.tif_4.png]

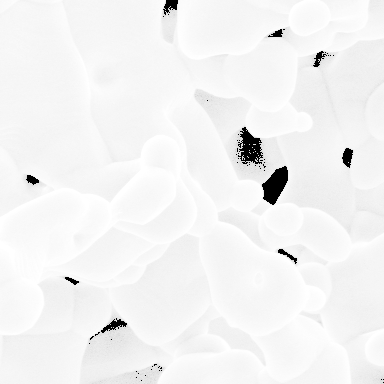

Supplement: S2 File — (ZIP) [file pone.0306385.s002.zip › S2/train/15_1-10.tif_7.png]

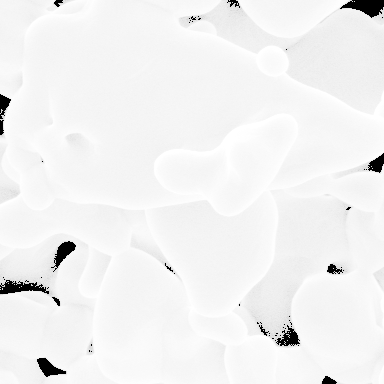

Supplement: S2 File — (ZIP) [file pone.0306385.s002.zip › S2/train/15_1-10.tif_8.png]

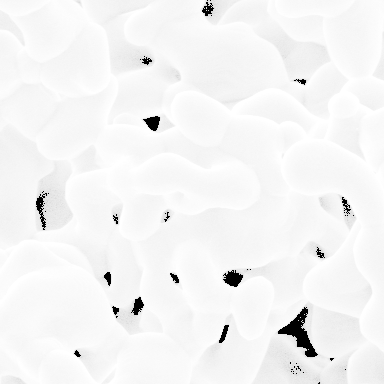

Supplement: S2 File — (ZIP) [file pone.0306385.s002.zip › S2/train/15_1-10.tif_9.png]

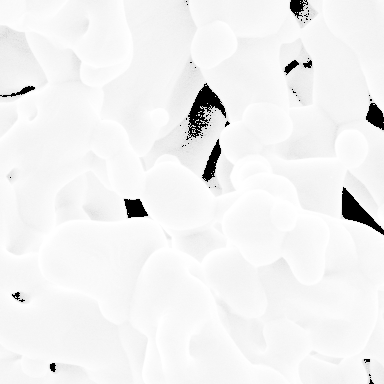

Supplement: S2 File — (ZIP) [file pone.0306385.s002.zip › S2/train/15_1-11.tif_1.png]

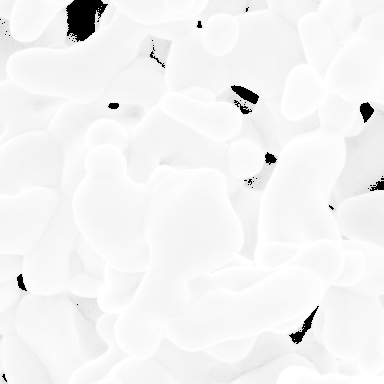

Supplement: S2 File — (ZIP) [file pone.0306385.s002.zip › S2/train/15_1-11.tif_3.png]

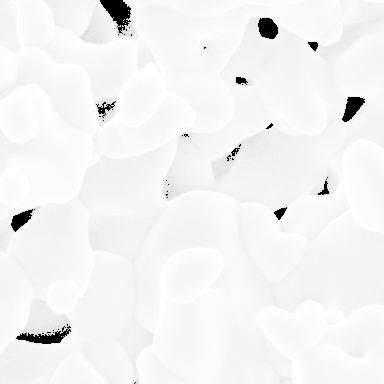

Supplement: S2 File — (ZIP) [file pone.0306385.s002.zip › S2/train/15_1-11.tif_5.png]

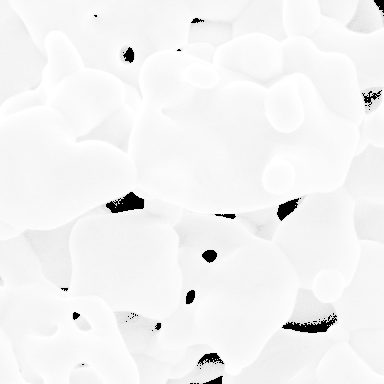

Supplement: S2 File — (ZIP) [file pone.0306385.s002.zip › S2/train/15_1-11.tif_7.png]

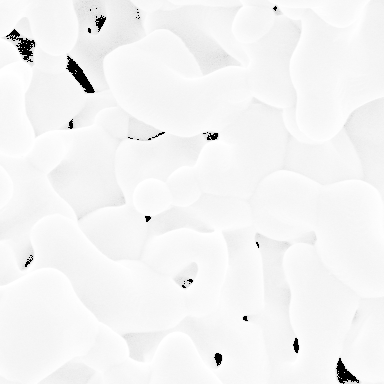

Supplement: S2 File — (ZIP) [file pone.0306385.s002.zip › S2/train/15_1-11.tif_8.png]

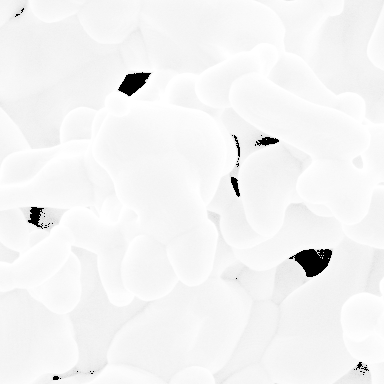

Supplement: S2 File — (ZIP) [file pone.0306385.s002.zip › S2/train/15_1-11.tif_9.png]

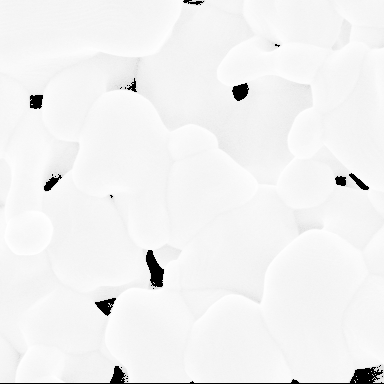

Supplement: S2 File — (ZIP) [file pone.0306385.s002.zip › S2/train/16_2-01.tif_1.png]

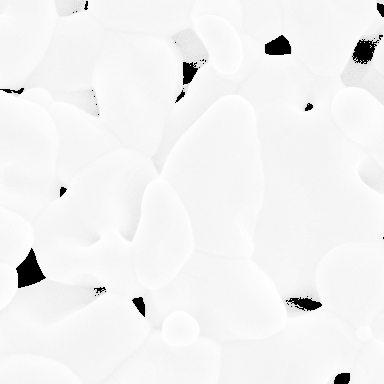

Supplement: S2 File — (ZIP) [file pone.0306385.s002.zip › S2/train/16_2-01.tif_10.png]

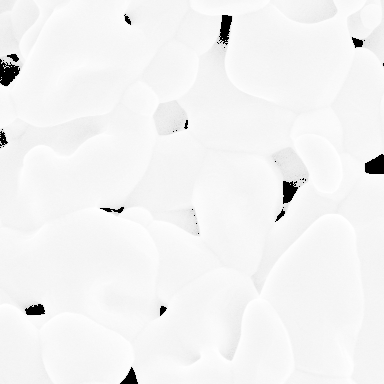

Supplement: S2 File — (ZIP) [file pone.0306385.s002.zip › S2/train/16_2-01.tif_3.png]

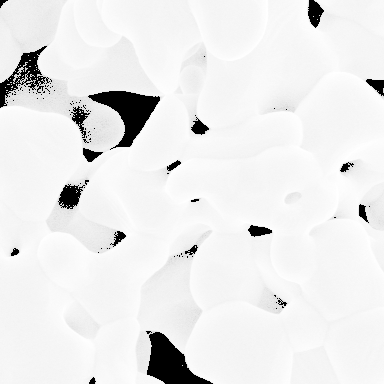

Supplement: S2 File — (ZIP) [file pone.0306385.s002.zip › S2/train/16_2-01.tif_4.png]

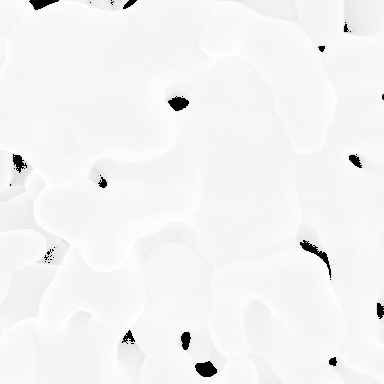

Supplement: S2 File — (ZIP) [file pone.0306385.s002.zip › S2/train/16_2-01.tif_5.png]

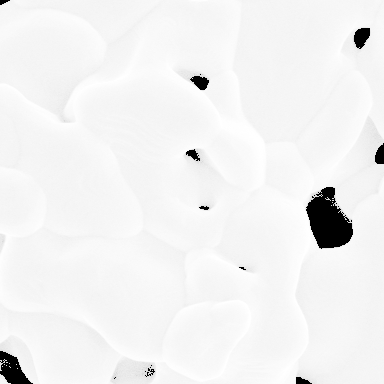

Supplement: S2 File — (ZIP) [file pone.0306385.s002.zip › S2/train/16_2-01.tif_6.png]

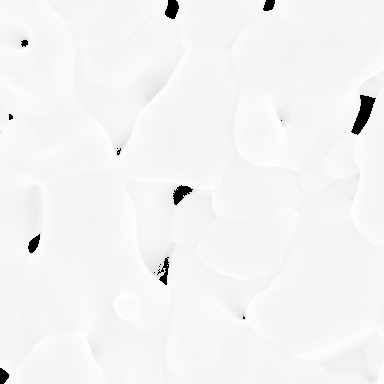

Supplement: S2 File — (ZIP) [file pone.0306385.s002.zip › S2/train/16_2-01.tif_8.png]

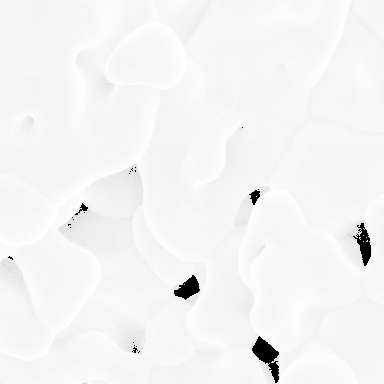

Supplement: S2 File — (ZIP) [file pone.0306385.s002.zip › S2/train/16_2_s-01.tif_10.png]

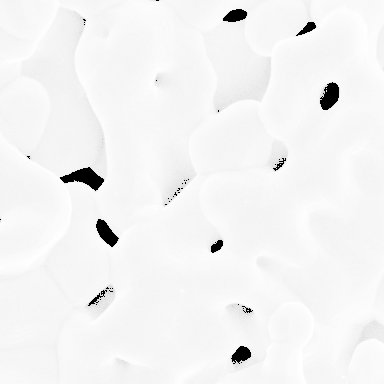

Supplement: S2 File — (ZIP) [file pone.0306385.s002.zip › S2/train/16_2_s-01.tif_2.png]

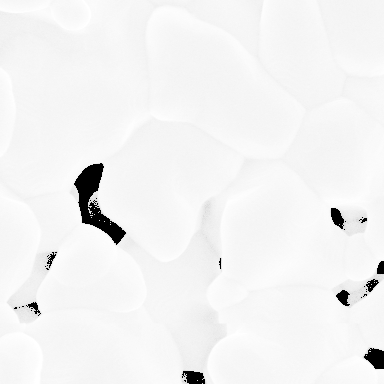

Supplement: S2 File — (ZIP) [file pone.0306385.s002.zip › S2/train/16_2_s-01.tif_6.png]

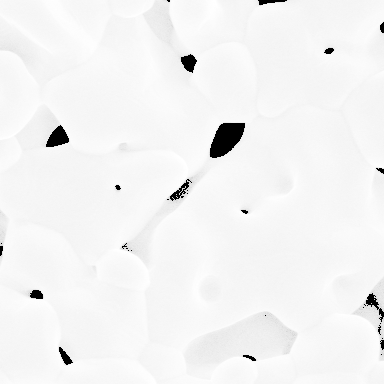

Supplement: S2 File — (ZIP) [file pone.0306385.s002.zip › S2/train/16_2_s-01.tif_7.png]

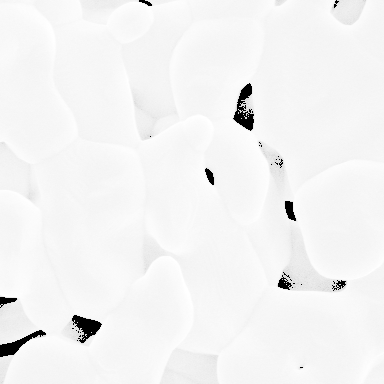

Supplement: S2 File — (ZIP) [file pone.0306385.s002.zip › S2/train/16_2_s-01.tif_8.png]

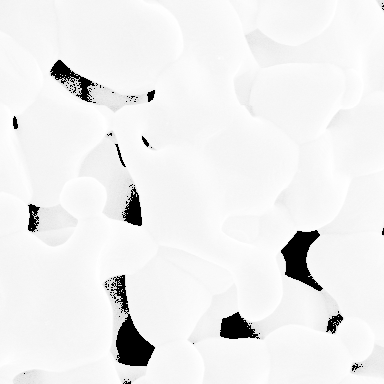

Supplement: S2 File — (ZIP) [file pone.0306385.s002.zip › S2/train/16_2_s-01.tif_9.png]

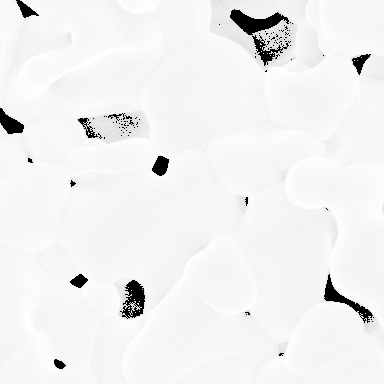

Supplement: S2 File — (ZIP) [file pone.0306385.s002.zip › S2/train/16_2_s-02.tif_2.png]

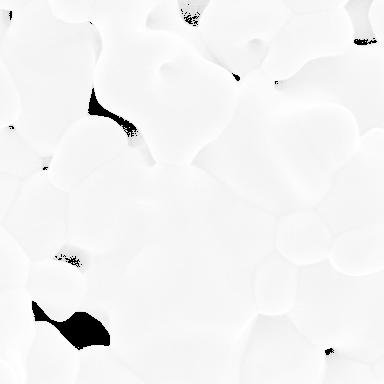

Supplement: S2 File — (ZIP) [file pone.0306385.s002.zip › S2/train/16_2_s-02.tif_3.png]

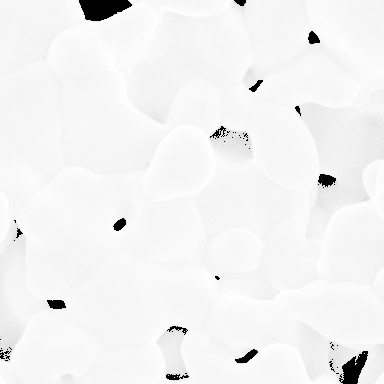

Supplement: S2 File — (ZIP) [file pone.0306385.s002.zip › S2/train/16_2_s-02.tif_4.png]

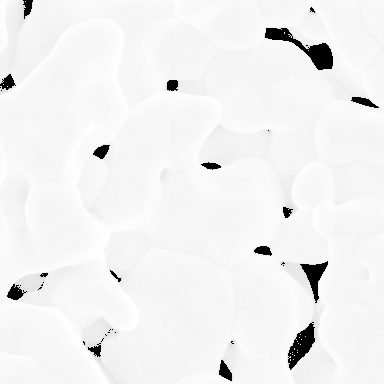

Supplement: S2 File — (ZIP) [file pone.0306385.s002.zip › S2/train/16_2_s-02.tif_5.png]

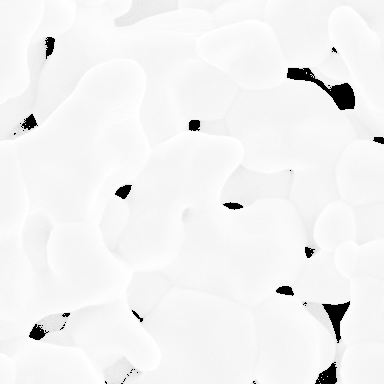

Supplement: S2 File — (ZIP) [file pone.0306385.s002.zip › S2/train/16_2_s-02.tif_6.png]

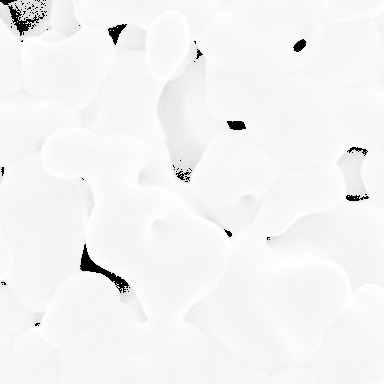

Supplement: S2 File — (ZIP) [file pone.0306385.s002.zip › S2/train/16_2_s-02.tif_7.png]

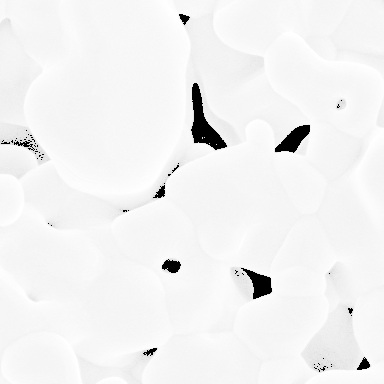

Supplement: S2 File — (ZIP) [file pone.0306385.s002.zip › S2/train/16_2_s-02.tif_9.png]

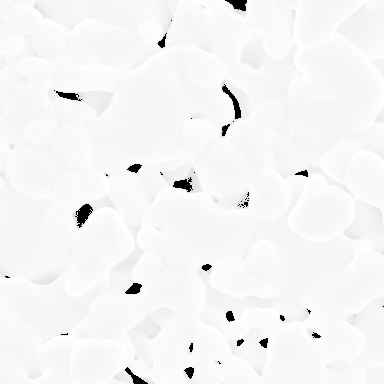

Supplement: S2 File — (ZIP) [file pone.0306385.s002.zip › S2/train/16_2_s-03.tif_1.png]

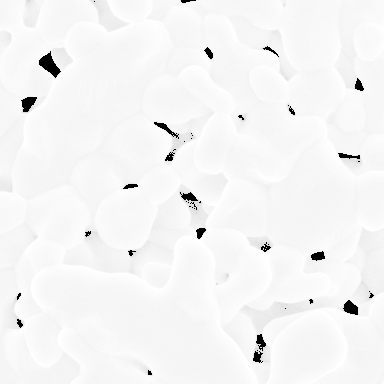

Supplement: S2 File — (ZIP) [file pone.0306385.s002.zip › S2/train/16_2_s-03.tif_10.png]

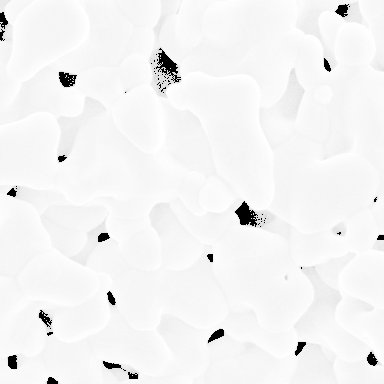

Supplement: S2 File — (ZIP) [file pone.0306385.s002.zip › S2/train/16_2_s-03.tif_4.png]

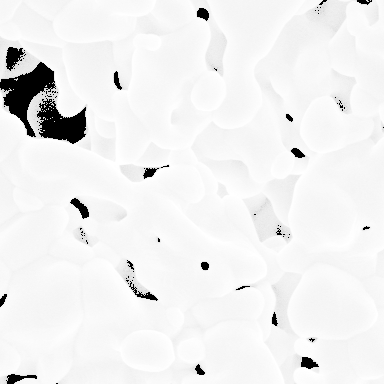

Supplement: S2 File — (ZIP) [file pone.0306385.s002.zip › S2/train/16_2_s-03.tif_5.png]

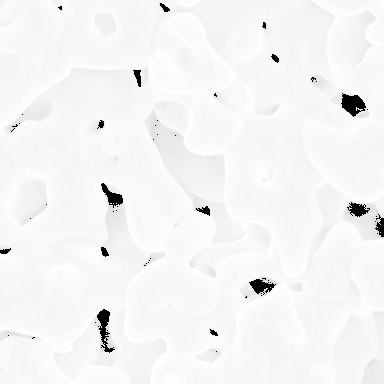

Supplement: S2 File — (ZIP) [file pone.0306385.s002.zip › S2/train/16_2_s-03.tif_6.png]

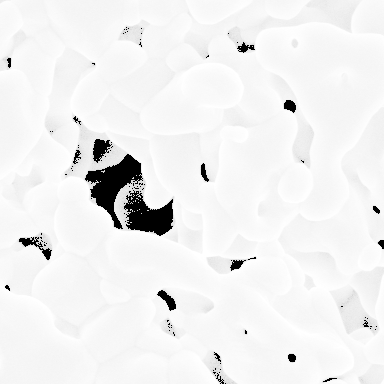

Supplement: S2 File — (ZIP) [file pone.0306385.s002.zip › S2/train/16_2_s-03.tif_8.png]

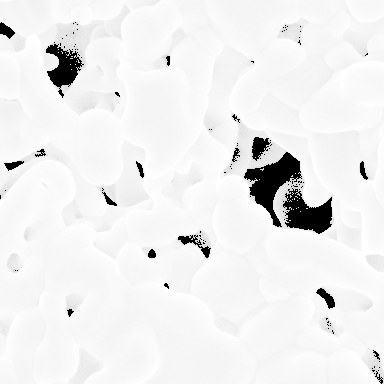

Supplement: S2 File — (ZIP) [file pone.0306385.s002.zip › S2/train/16_2_s-03.tif_9.png]

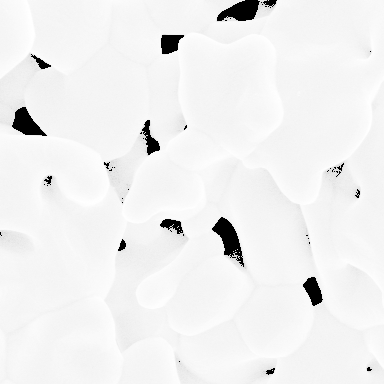

Supplement: S2 File — (ZIP) [file pone.0306385.s002.zip › S2/train/16_2_x-01.tif_1.png]

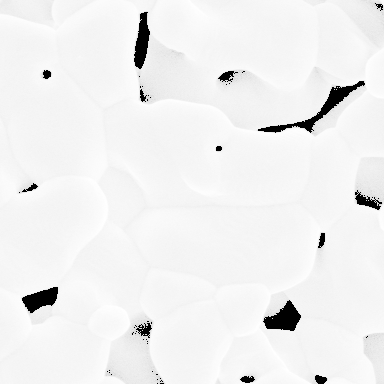

Supplement: S2 File — (ZIP) [file pone.0306385.s002.zip › S2/train/16_2_x-01.tif_2.png]

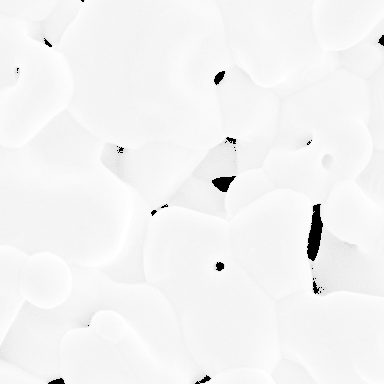

Supplement: S2 File — (ZIP) [file pone.0306385.s002.zip › S2/train/16_2_x-01.tif_4.png]

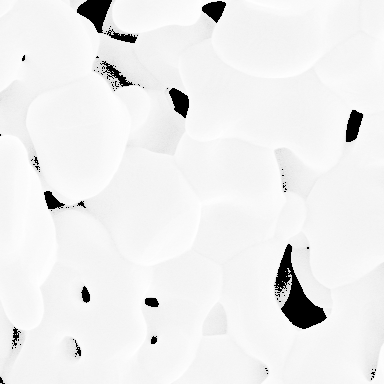

Supplement: S2 File — (ZIP) [file pone.0306385.s002.zip › S2/train/16_2_x-01.tif_5.png]

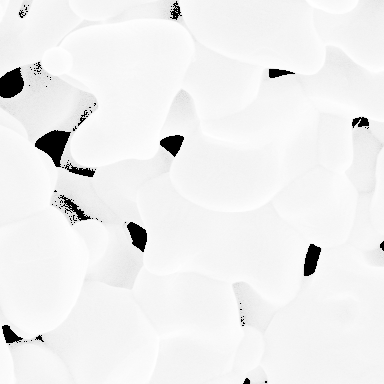

Supplement: S2 File — (ZIP) [file pone.0306385.s002.zip › S2/train/16_2_x-01.tif_6.png]

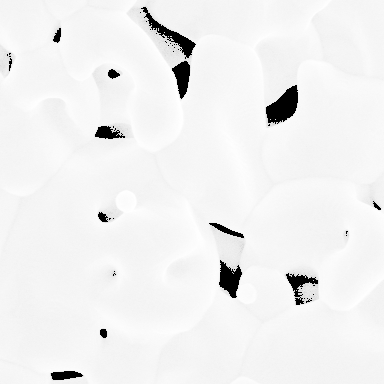

Supplement: S2 File — (ZIP) [file pone.0306385.s002.zip › S2/train/16_2_x-01.tif_7.png]

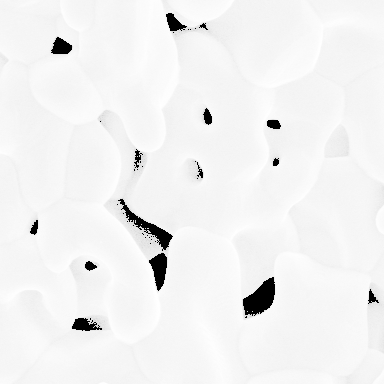

Supplement: S2 File — (ZIP) [file pone.0306385.s002.zip › S2/train/16_2_x-01.tif_8.png]

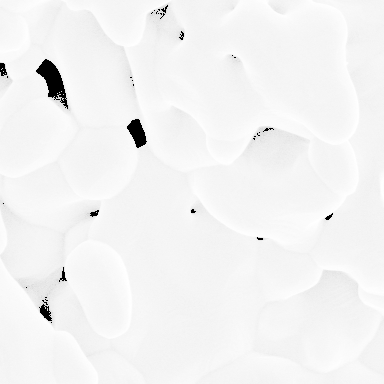

Supplement: S2 File — (ZIP) [file pone.0306385.s002.zip › S2/train/16_2_x-01.tif_9.png]

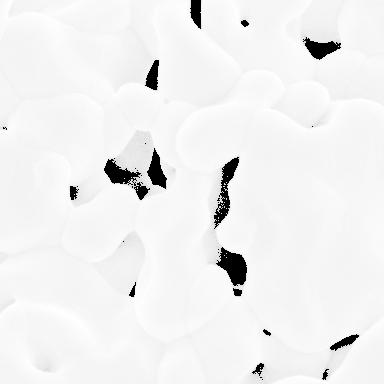

Supplement: S2 File — (ZIP) [file pone.0306385.s002.zip › S2/train/16_2_x-02.tif_10.png]

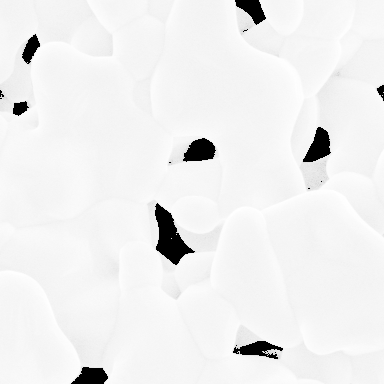

Supplement: S2 File — (ZIP) [file pone.0306385.s002.zip › S2/train/16_2_x-02.tif_3.png]

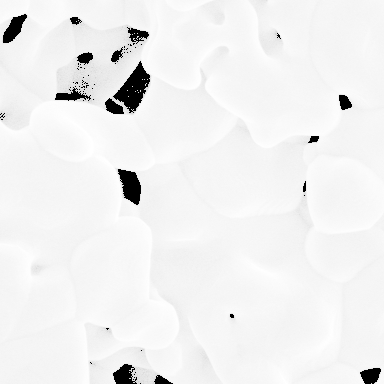

Supplement: S2 File — (ZIP) [file pone.0306385.s002.zip › S2/train/16_2_x-02.tif_5.png]
