# Supplementary figures and images for: Deep-learning-based pyramid-transformer for localized porosity analysis of hot-press sintered ceramic paste (part 3 of 3)
Source: PLoS One. 2024 Sep 4;19(9):e0306385. doi: 10.1371/journal.pone.0306385 (PMC11373816; doi:10.1371/journal.pone.0306385)

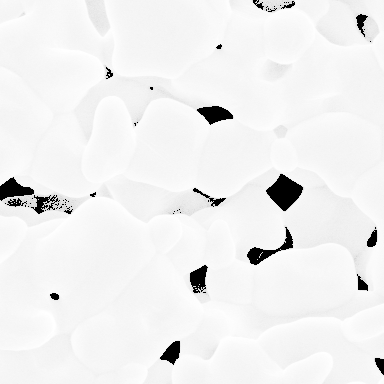

Supplement: S2 File — (ZIP) [file pone.0306385.s002.zip › S2/val/16_2_x-03.tif_3.png]

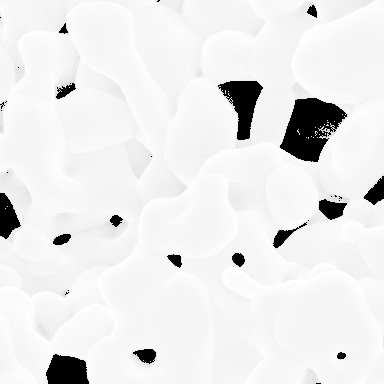

Supplement: S2 File — (ZIP) [file pone.0306385.s002.zip › S2/val/16_2_x-03.tif_6.png]

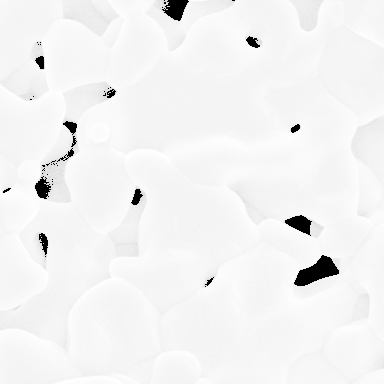

Supplement: S2 File — (ZIP) [file pone.0306385.s002.zip › S2/val/16_2_x-03.tif_9.png]

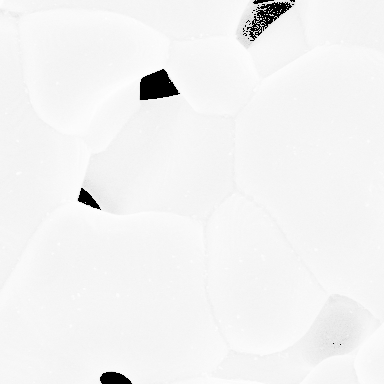

Supplement: S2 File — (ZIP) [file pone.0306385.s002.zip › S2/val/17_3-01.tif_1.png]

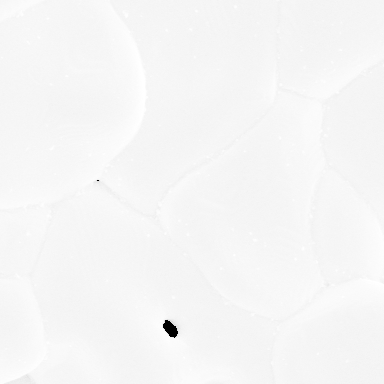

Supplement: S2 File — (ZIP) [file pone.0306385.s002.zip › S2/val/17_3-01.tif_10.png]

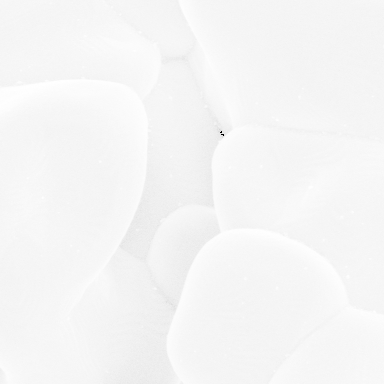

Supplement: S2 File — (ZIP) [file pone.0306385.s002.zip › S2/val/17_3-01.tif_3.png]

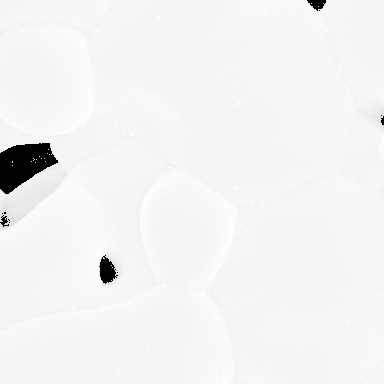

Supplement: S2 File — (ZIP) [file pone.0306385.s002.zip › S2/val/17_3-01.tif_5.png]

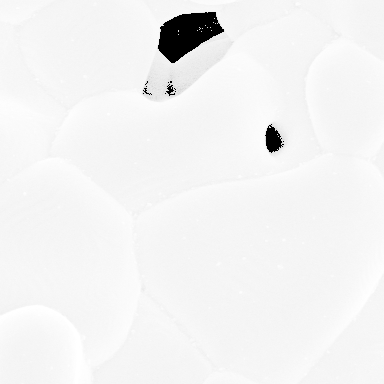

Supplement: S2 File — (ZIP) [file pone.0306385.s002.zip › S2/val/17_3-01.tif_9.png]

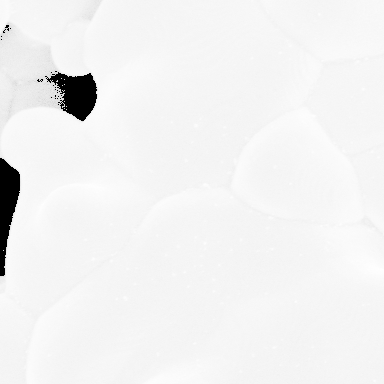

Supplement: S2 File — (ZIP) [file pone.0306385.s002.zip › S2/val/17_3_s-01.tif_4.png]

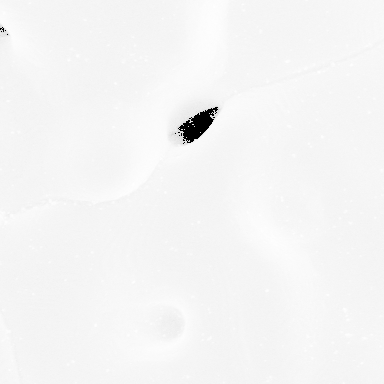

Supplement: S2 File — (ZIP) [file pone.0306385.s002.zip › S2/val/17_3_s-01.tif_6.png]

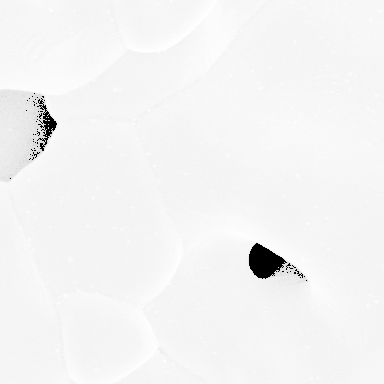

Supplement: S2 File — (ZIP) [file pone.0306385.s002.zip › S2/val/17_3_s-01.tif_8.png]

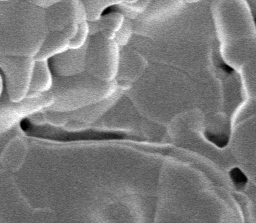

Supplement: S3 File — (ZIP) [file pone.0306385.s003.zip › S3/2_1.tif]

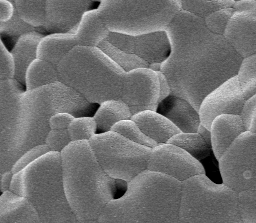

Supplement: S3 File — (ZIP) [file pone.0306385.s003.zip › S3/2_10.tif]

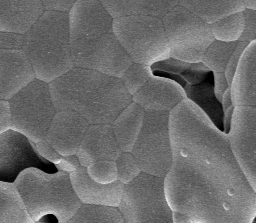

Supplement: S3 File — (ZIP) [file pone.0306385.s003.zip › S3/2_11.tif]

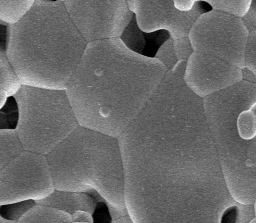

Supplement: S3 File — (ZIP) [file pone.0306385.s003.zip › S3/2_12.tif]

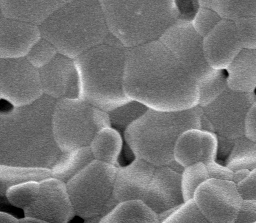

Supplement: S3 File — (ZIP) [file pone.0306385.s003.zip › S3/2_13.tif]

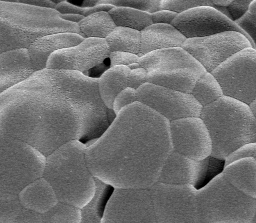

Supplement: S3 File — (ZIP) [file pone.0306385.s003.zip › S3/2_14.tif]

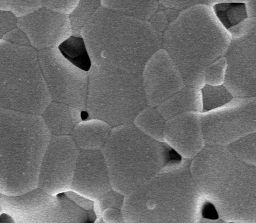

Supplement: S3 File — (ZIP) [file pone.0306385.s003.zip › S3/2_15.tif]

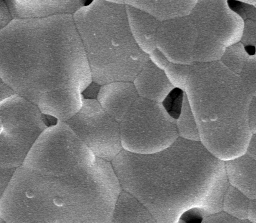

Supplement: S3 File — (ZIP) [file pone.0306385.s003.zip › S3/2_16.tif]

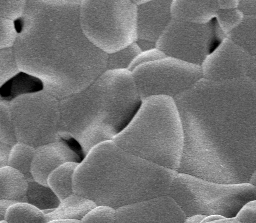

Supplement: S3 File — (ZIP) [file pone.0306385.s003.zip › S3/2_17.tif]

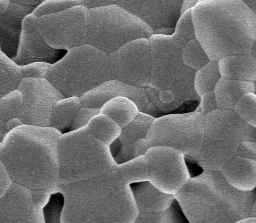

Supplement: S3 File — (ZIP) [file pone.0306385.s003.zip › S3/2_18.tif]

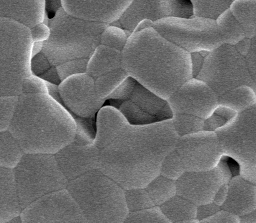

Supplement: S3 File — (ZIP) [file pone.0306385.s003.zip › S3/2_19.tif]

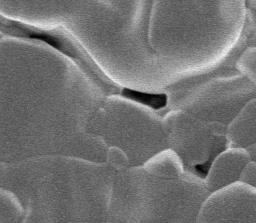

Supplement: S3 File — (ZIP) [file pone.0306385.s003.zip › S3/2_2.tif]

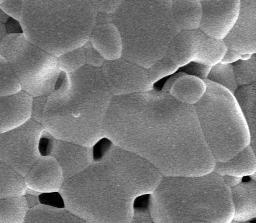

Supplement: S3 File — (ZIP) [file pone.0306385.s003.zip › S3/2_20.tif]

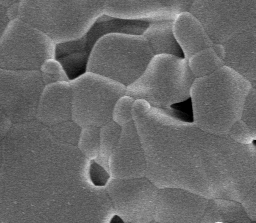

Supplement: S3 File — (ZIP) [file pone.0306385.s003.zip › S3/2_3.tif]

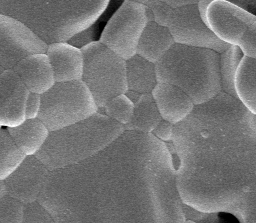

Supplement: S3 File — (ZIP) [file pone.0306385.s003.zip › S3/2_4.tif]

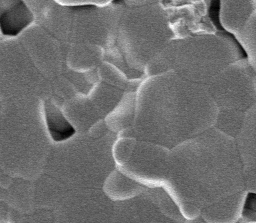

Supplement: S3 File — (ZIP) [file pone.0306385.s003.zip › S3/2_5.tif]

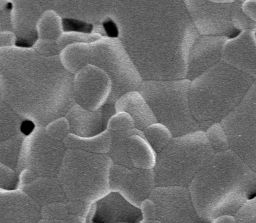

Supplement: S3 File — (ZIP) [file pone.0306385.s003.zip › S3/2_6.tif]

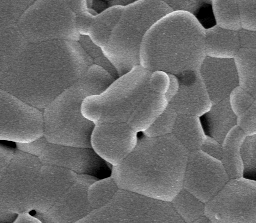

Supplement: S3 File — (ZIP) [file pone.0306385.s003.zip › S3/2_7.tif]

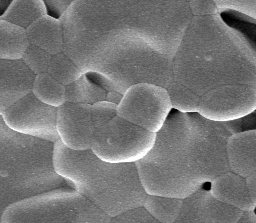

Supplement: S3 File — (ZIP) [file pone.0306385.s003.zip › S3/2_8.tif]

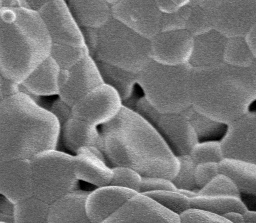

Supplement: S3 File — (ZIP) [file pone.0306385.s003.zip › S3/2_9.tif]

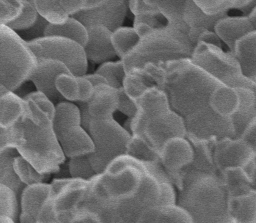

Supplement: S3 File — (ZIP) [file pone.0306385.s003.zip › S3/30_1.tif]

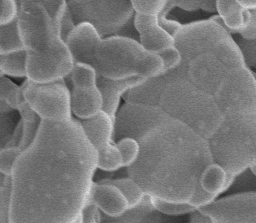

Supplement: S3 File — (ZIP) [file pone.0306385.s003.zip › S3/30_10.tif]

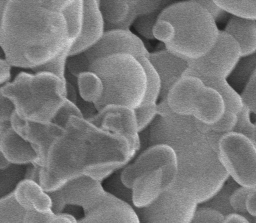

Supplement: S3 File — (ZIP) [file pone.0306385.s003.zip › S3/30_11.tif]

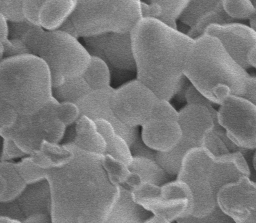

Supplement: S3 File — (ZIP) [file pone.0306385.s003.zip › S3/30_12.tif]

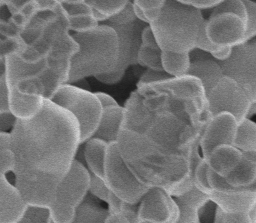

Supplement: S3 File — (ZIP) [file pone.0306385.s003.zip › S3/30_13.tif]

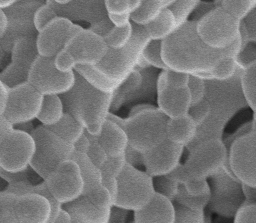

Supplement: S3 File — (ZIP) [file pone.0306385.s003.zip › S3/30_14.tif]

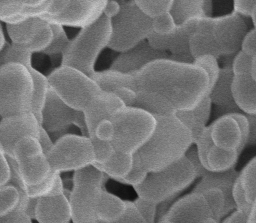

Supplement: S3 File — (ZIP) [file pone.0306385.s003.zip › S3/30_15.tif]

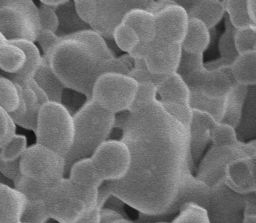

Supplement: S3 File — (ZIP) [file pone.0306385.s003.zip › S3/30_16.tif]

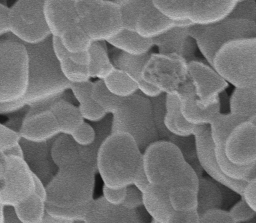

Supplement: S3 File — (ZIP) [file pone.0306385.s003.zip › S3/30_17.tif]

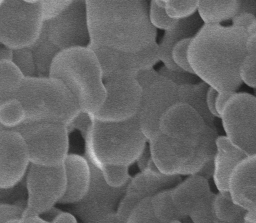

Supplement: S3 File — (ZIP) [file pone.0306385.s003.zip › S3/30_18.tif]

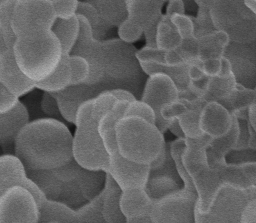

Supplement: S3 File — (ZIP) [file pone.0306385.s003.zip › S3/30_19.tif]

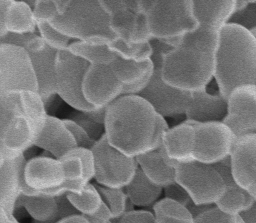

Supplement: S3 File — (ZIP) [file pone.0306385.s003.zip › S3/30_2.tif]

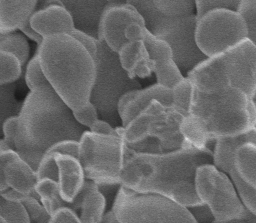

Supplement: S3 File — (ZIP) [file pone.0306385.s003.zip › S3/30_20.tif]

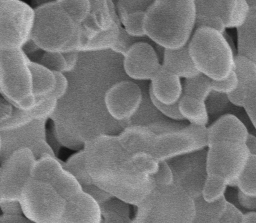

Supplement: S3 File — (ZIP) [file pone.0306385.s003.zip › S3/30_3.tif]

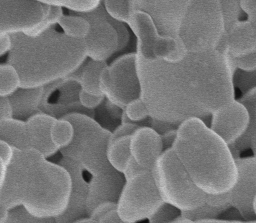

Supplement: S3 File — (ZIP) [file pone.0306385.s003.zip › S3/30_4.tif]

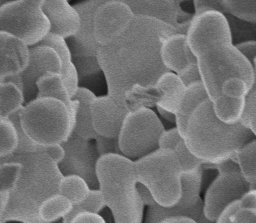

Supplement: S3 File — (ZIP) [file pone.0306385.s003.zip › S3/30_5.tif]

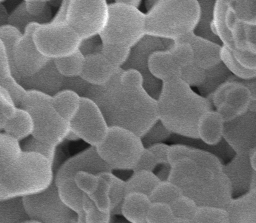

Supplement: S3 File — (ZIP) [file pone.0306385.s003.zip › S3/30_6.tif]

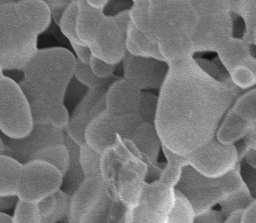

Supplement: S3 File — (ZIP) [file pone.0306385.s003.zip › S3/30_7.tif]

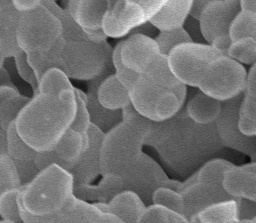

Supplement: S3 File — (ZIP) [file pone.0306385.s003.zip › S3/30_8.tif]

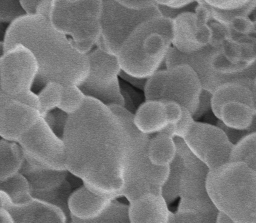

Supplement: S3 File — (ZIP) [file pone.0306385.s003.zip › S3/30_9.tif]
