# Supplementary figures and images for: Deep-learning-based pyramid-transformer for localized porosity analysis of hot-press sintered ceramic paste (part 2 of 3)
Source: PLoS One. 2024 Sep 4;19(9):e0306385. doi: 10.1371/journal.pone.0306385 (PMC11373816; doi:10.1371/journal.pone.0306385)

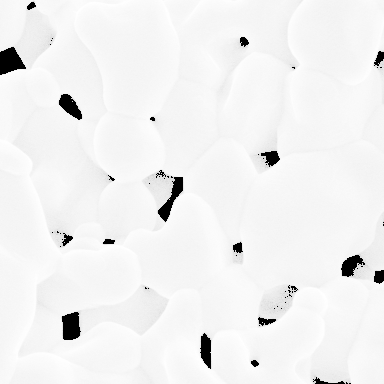

Supplement: S2 File — (ZIP) [file pone.0306385.s002.zip › S2/train/16_2_x-02.tif_7.png]

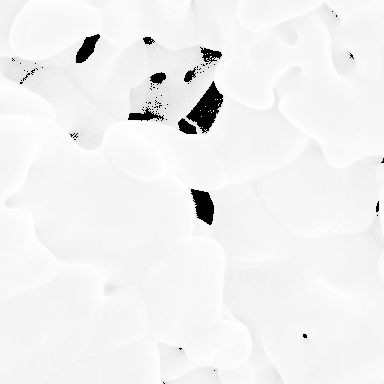

Supplement: S2 File — (ZIP) [file pone.0306385.s002.zip › S2/train/16_2_x-02.tif_8.png]

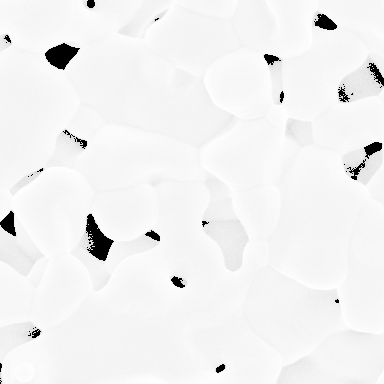

Supplement: S2 File — (ZIP) [file pone.0306385.s002.zip › S2/train/16_2_x-03.tif_10.png]

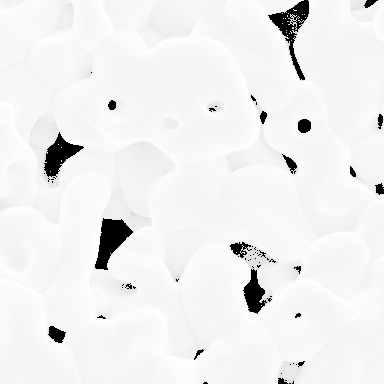

Supplement: S2 File — (ZIP) [file pone.0306385.s002.zip › S2/train/16_2_x-03.tif_2.png]

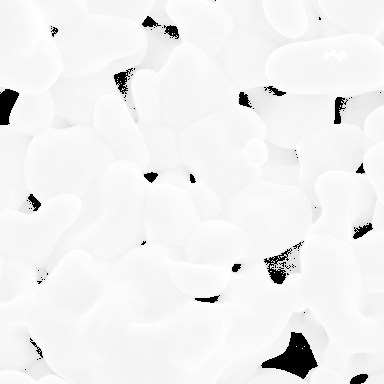

Supplement: S2 File — (ZIP) [file pone.0306385.s002.zip › S2/train/16_2_x-03.tif_4.png]

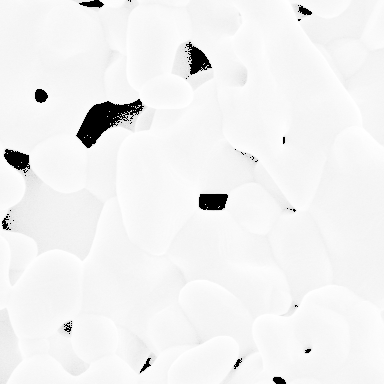

Supplement: S2 File — (ZIP) [file pone.0306385.s002.zip › S2/train/16_2_x-03.tif_5.png]

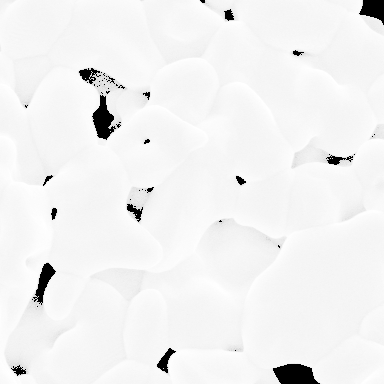

Supplement: S2 File — (ZIP) [file pone.0306385.s002.zip › S2/train/16_2_x-03.tif_7.png]

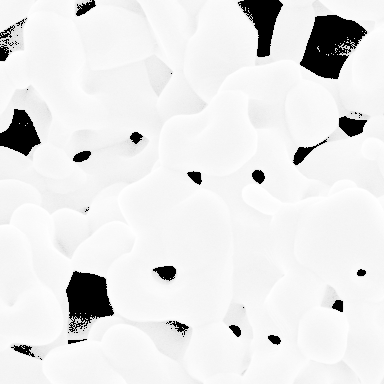

Supplement: S2 File — (ZIP) [file pone.0306385.s002.zip › S2/train/16_2_x-03.tif_8.png]

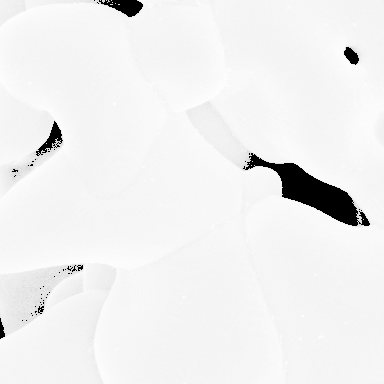

Supplement: S2 File — (ZIP) [file pone.0306385.s002.zip › S2/train/17_3-01.tif_2.png]

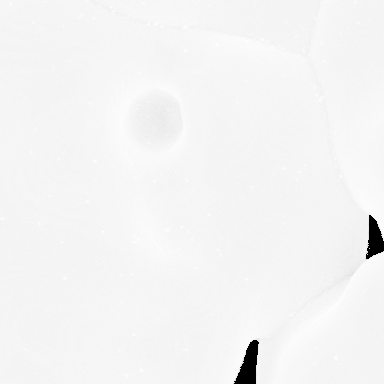

Supplement: S2 File — (ZIP) [file pone.0306385.s002.zip › S2/train/17_3-01.tif_4.png]

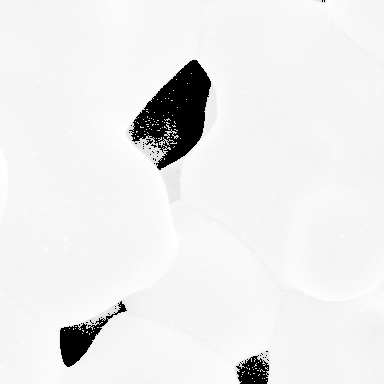

Supplement: S2 File — (ZIP) [file pone.0306385.s002.zip › S2/train/17_3-01.tif_6.png]

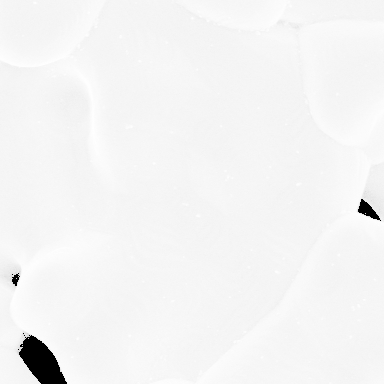

Supplement: S2 File — (ZIP) [file pone.0306385.s002.zip › S2/train/17_3-01.tif_7.png]

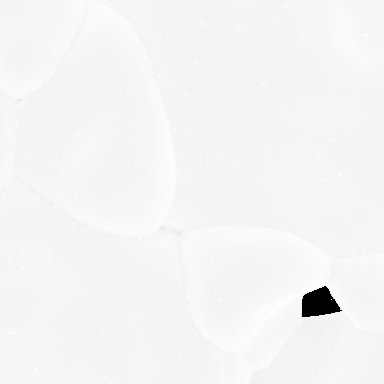

Supplement: S2 File — (ZIP) [file pone.0306385.s002.zip › S2/train/17_3-01.tif_8.png]

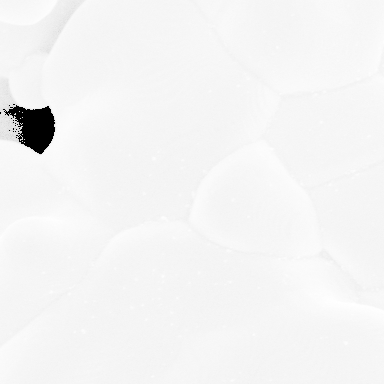

Supplement: S2 File — (ZIP) [file pone.0306385.s002.zip › S2/train/17_3_s-01.tif_1.png]

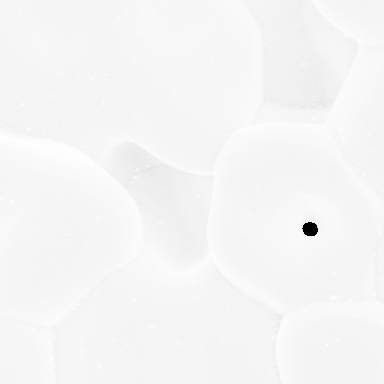

Supplement: S2 File — (ZIP) [file pone.0306385.s002.zip › S2/train/17_3_s-01.tif_10.png]

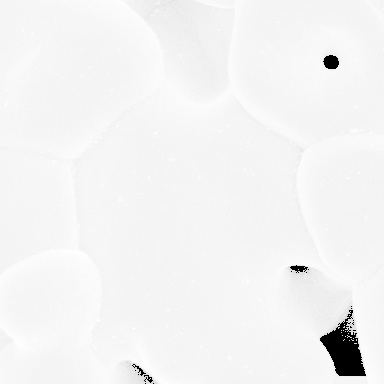

Supplement: S2 File — (ZIP) [file pone.0306385.s002.zip › S2/train/17_3_s-01.tif_2.png]

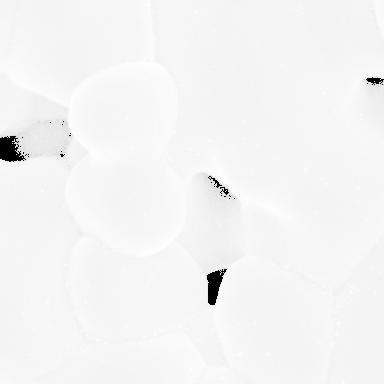

Supplement: S2 File — (ZIP) [file pone.0306385.s002.zip › S2/train/17_3_s-01.tif_3.png]

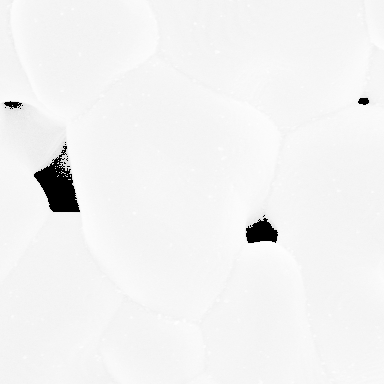

Supplement: S2 File — (ZIP) [file pone.0306385.s002.zip › S2/train/17_3_s-01.tif_5.png]

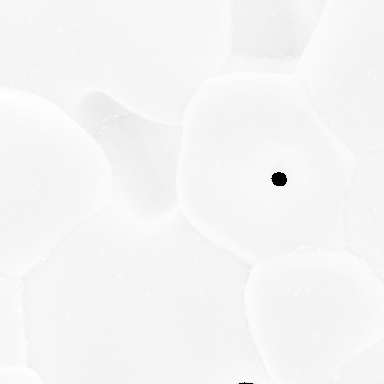

Supplement: S2 File — (ZIP) [file pone.0306385.s002.zip › S2/train/17_3_s-01.tif_7.png]

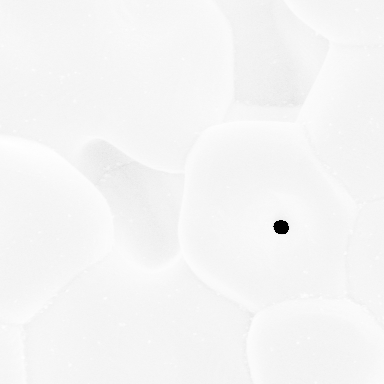

Supplement: S2 File — (ZIP) [file pone.0306385.s002.zip › S2/train/17_3_s-01.tif_9.png]

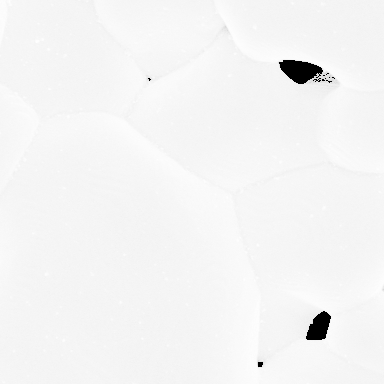

Supplement: S2 File — (ZIP) [file pone.0306385.s002.zip › S2/train/17_3_s-02.tif_10.png]

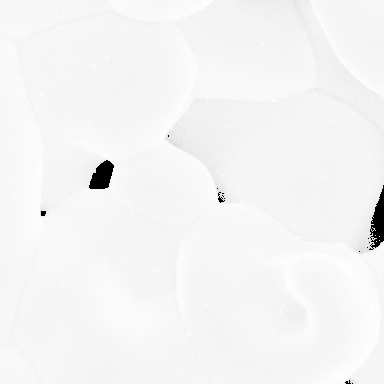

Supplement: S2 File — (ZIP) [file pone.0306385.s002.zip › S2/train/17_3_s-02.tif_2.png]

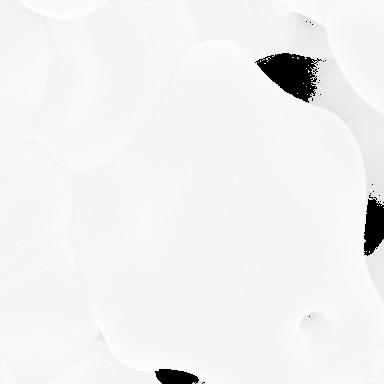

Supplement: S2 File — (ZIP) [file pone.0306385.s002.zip › S2/train/17_3_s-02.tif_4.png]

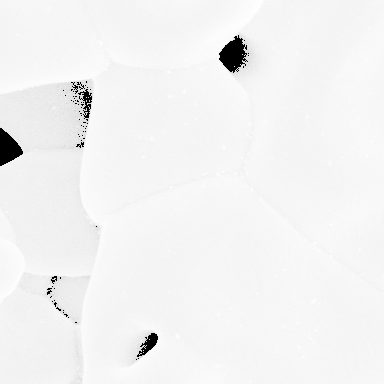

Supplement: S2 File — (ZIP) [file pone.0306385.s002.zip › S2/train/17_3_s-02.tif_5.png]

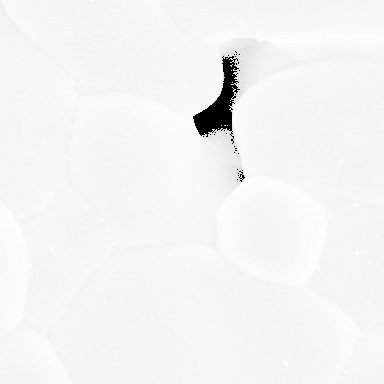

Supplement: S2 File — (ZIP) [file pone.0306385.s002.zip › S2/train/17_3_s-02.tif_6.png]

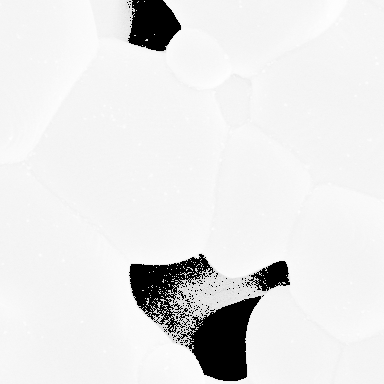

Supplement: S2 File — (ZIP) [file pone.0306385.s002.zip › S2/train/17_3_s-02.tif_7.png]

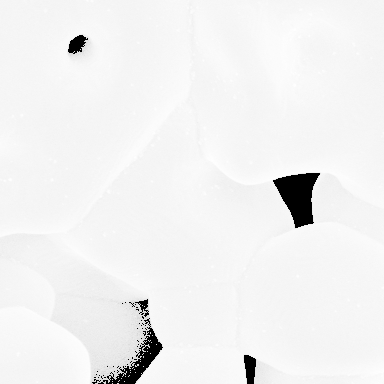

Supplement: S2 File — (ZIP) [file pone.0306385.s002.zip › S2/train/17_3_s-02.tif_8.png]

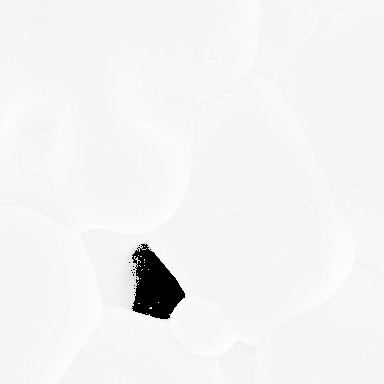

Supplement: S2 File — (ZIP) [file pone.0306385.s002.zip › S2/train/17_3_s-02.tif_9.png]

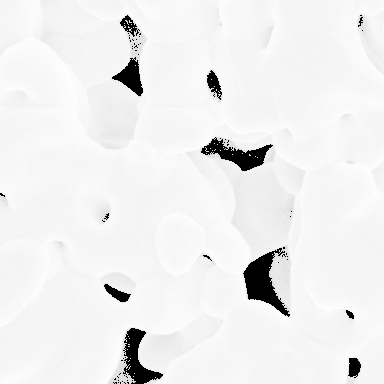

Supplement: S2 File — (ZIP) [file pone.0306385.s002.zip › S2/train/17_3_s-03.tif_1.png]

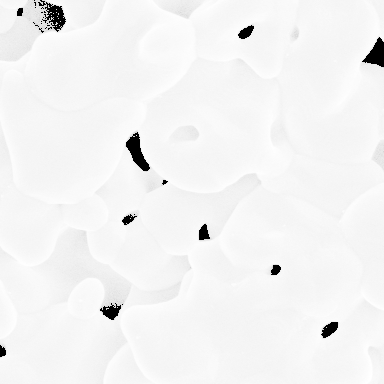

Supplement: S2 File — (ZIP) [file pone.0306385.s002.zip › S2/train/17_3_s-03.tif_10.png]

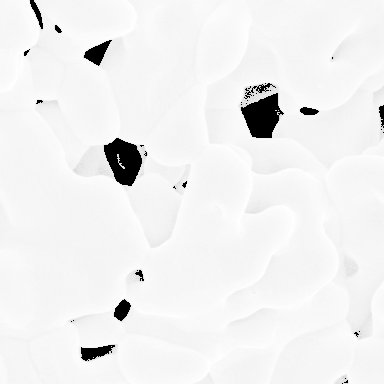

Supplement: S2 File — (ZIP) [file pone.0306385.s002.zip › S2/train/17_3_s-03.tif_2.png]

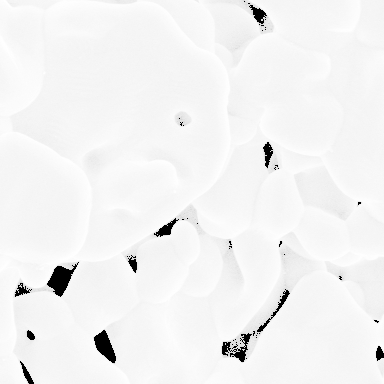

Supplement: S2 File — (ZIP) [file pone.0306385.s002.zip › S2/train/17_3_s-03.tif_4.png]

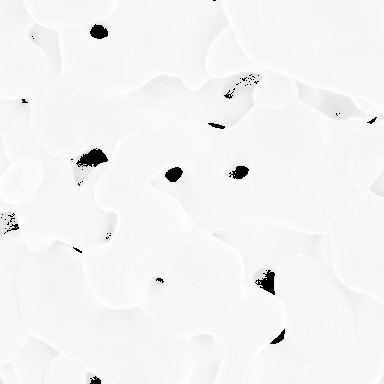

Supplement: S2 File — (ZIP) [file pone.0306385.s002.zip › S2/train/17_3_s-03.tif_6.png]

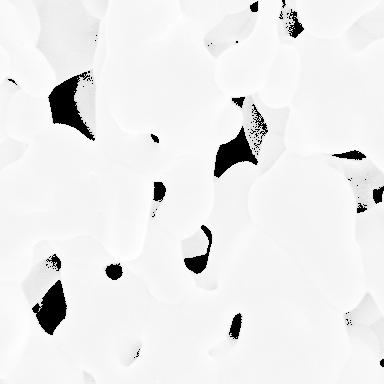

Supplement: S2 File — (ZIP) [file pone.0306385.s002.zip › S2/train/17_3_s-03.tif_7.png]

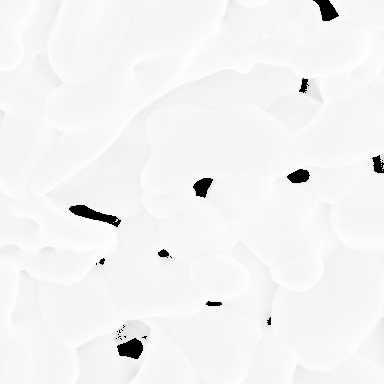

Supplement: S2 File — (ZIP) [file pone.0306385.s002.zip › S2/train/17_3_s-03.tif_8.png]

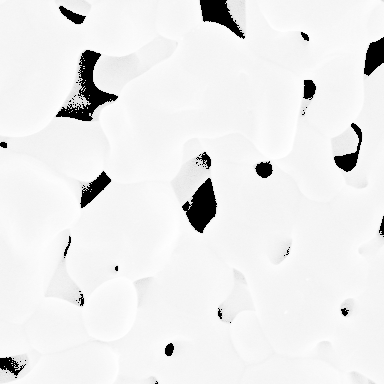

Supplement: S2 File — (ZIP) [file pone.0306385.s002.zip › S2/train/17_3_s-03.tif_9.png]

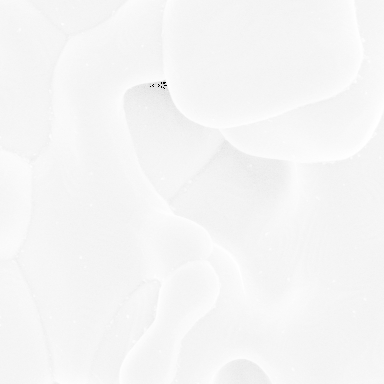

Supplement: S2 File — (ZIP) [file pone.0306385.s002.zip › S2/train/17_3_x-01.tif_10.png]

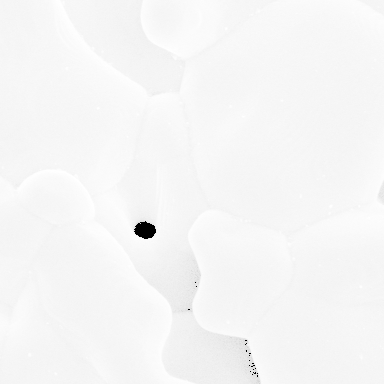

Supplement: S2 File — (ZIP) [file pone.0306385.s002.zip › S2/train/17_3_x-01.tif_2.png]

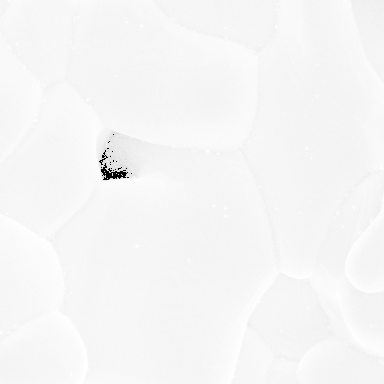

Supplement: S2 File — (ZIP) [file pone.0306385.s002.zip › S2/train/17_3_x-01.tif_4.png]

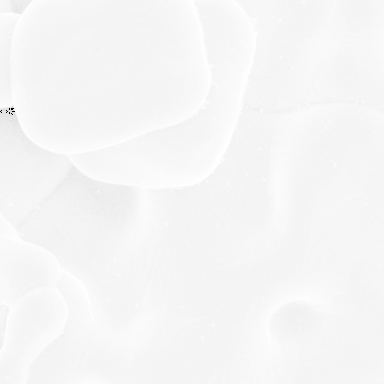

Supplement: S2 File — (ZIP) [file pone.0306385.s002.zip › S2/train/17_3_x-01.tif_5.png]

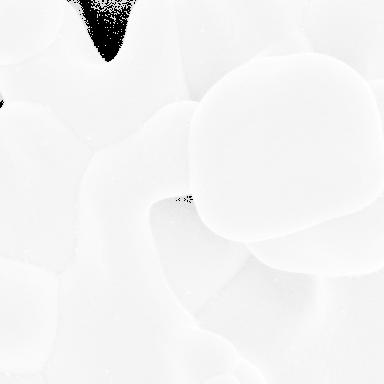

Supplement: S2 File — (ZIP) [file pone.0306385.s002.zip › S2/train/17_3_x-01.tif_6.png]

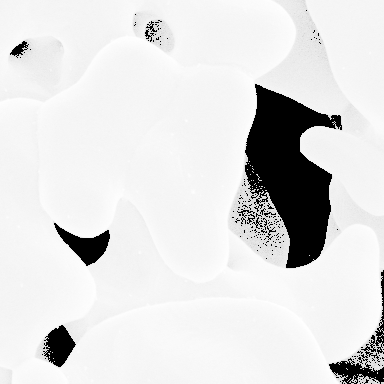

Supplement: S2 File — (ZIP) [file pone.0306385.s002.zip › S2/train/17_3_x-01.tif_7.png]

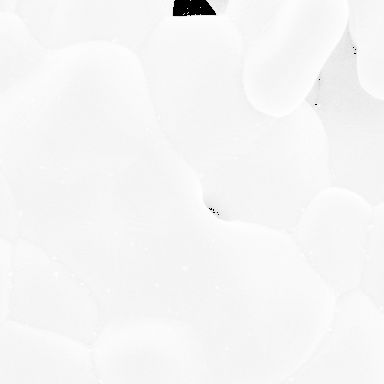

Supplement: S2 File — (ZIP) [file pone.0306385.s002.zip › S2/train/17_3_x-01.tif_8.png]

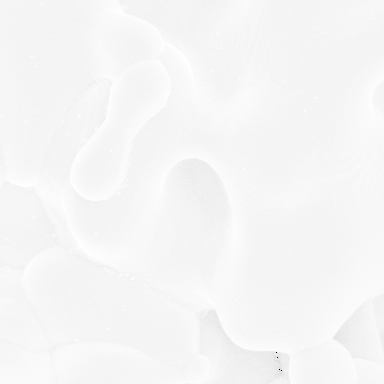

Supplement: S2 File — (ZIP) [file pone.0306385.s002.zip › S2/train/17_3_x-01.tif_9.png]

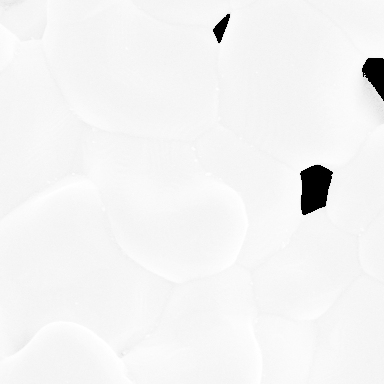

Supplement: S2 File — (ZIP) [file pone.0306385.s002.zip › S2/train/17_3_x-02.tif_1.png]

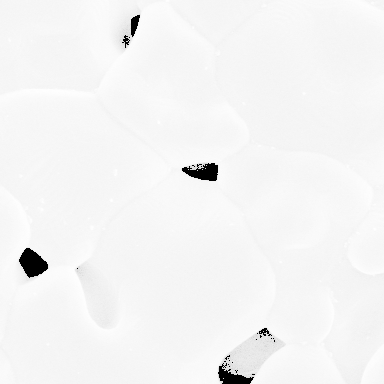

Supplement: S2 File — (ZIP) [file pone.0306385.s002.zip › S2/train/17_3_x-02.tif_10.png]

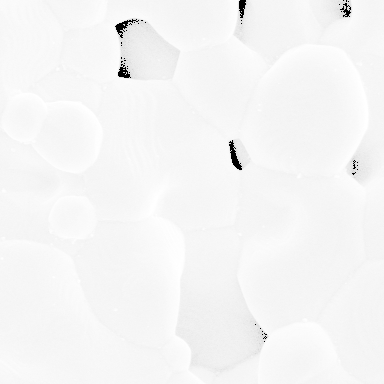

Supplement: S2 File — (ZIP) [file pone.0306385.s002.zip › S2/train/17_3_x-02.tif_2.png]

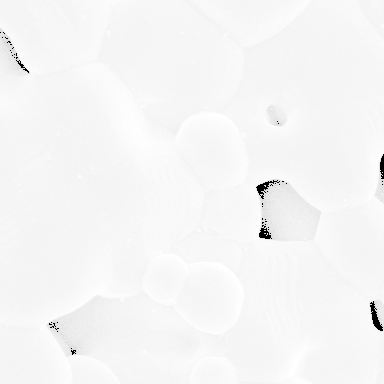

Supplement: S2 File — (ZIP) [file pone.0306385.s002.zip › S2/train/17_3_x-02.tif_3.png]

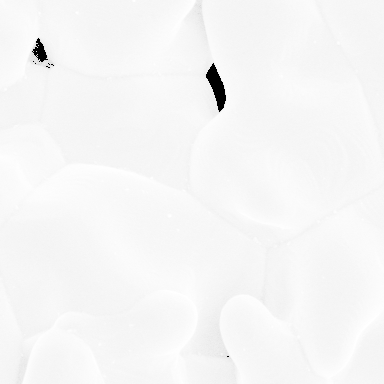

Supplement: S2 File — (ZIP) [file pone.0306385.s002.zip › S2/train/17_3_x-02.tif_5.png]

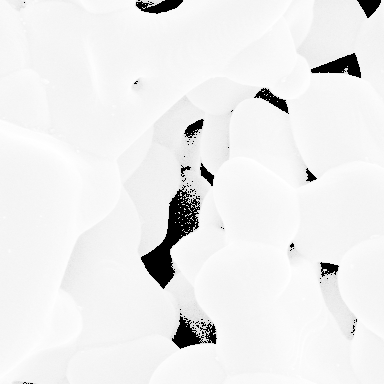

Supplement: S2 File — (ZIP) [file pone.0306385.s002.zip › S2/train/17_3_x-02.tif_6.png]

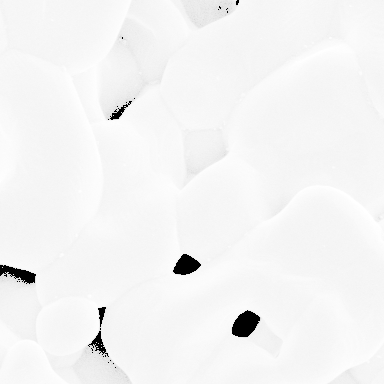

Supplement: S2 File — (ZIP) [file pone.0306385.s002.zip › S2/train/17_3_x-02.tif_7.png]

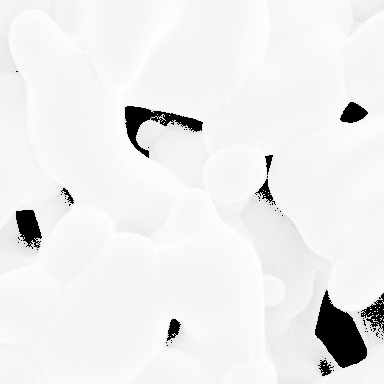

Supplement: S2 File — (ZIP) [file pone.0306385.s002.zip › S2/train/17_3_x-02.tif_8.png]

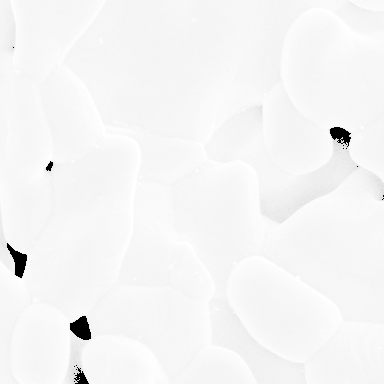

Supplement: S2 File — (ZIP) [file pone.0306385.s002.zip › S2/train/17_3_x-03.tif_10.png]

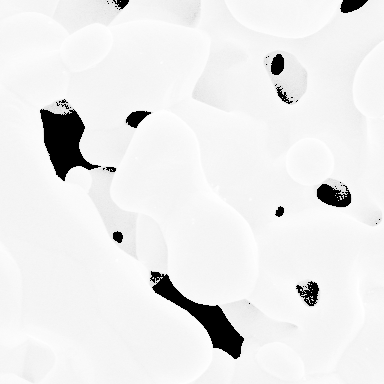

Supplement: S2 File — (ZIP) [file pone.0306385.s002.zip › S2/train/17_3_x-03.tif_2.png]

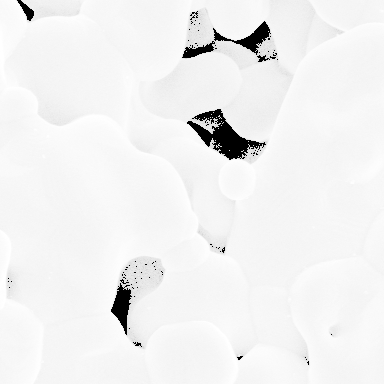

Supplement: S2 File — (ZIP) [file pone.0306385.s002.zip › S2/train/17_3_x-03.tif_3.png]

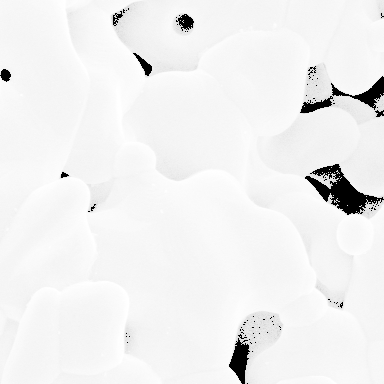

Supplement: S2 File — (ZIP) [file pone.0306385.s002.zip › S2/train/17_3_x-03.tif_4.png]

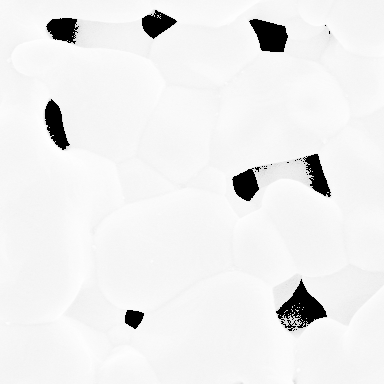

Supplement: S2 File — (ZIP) [file pone.0306385.s002.zip › S2/train/17_3_x-03.tif_6.png]

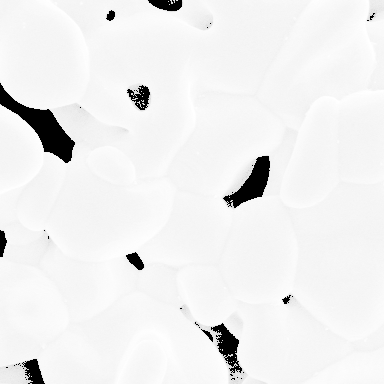

Supplement: S2 File — (ZIP) [file pone.0306385.s002.zip › S2/train/17_3_x-03.tif_7.png]

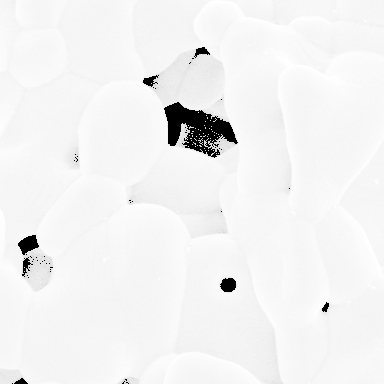

Supplement: S2 File — (ZIP) [file pone.0306385.s002.zip › S2/train/17_3_x-03.tif_9.png]

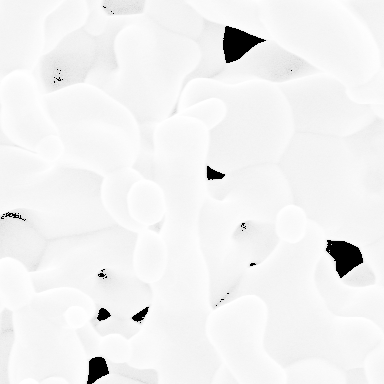

Supplement: S2 File — (ZIP) [file pone.0306385.s002.zip › S2/val/15_1-03.tif_1.png]

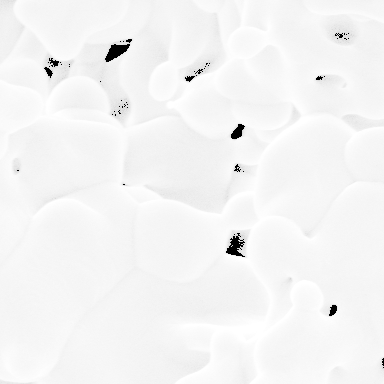

Supplement: S2 File — (ZIP) [file pone.0306385.s002.zip › S2/val/15_1-03.tif_3.png]

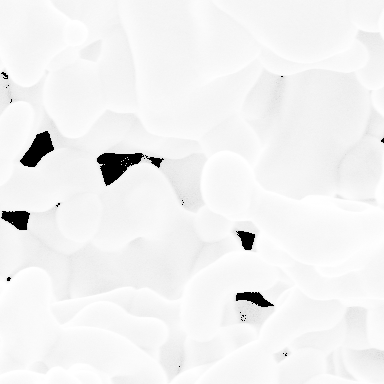

Supplement: S2 File — (ZIP) [file pone.0306385.s002.zip › S2/val/15_1-06.tif_1.png]

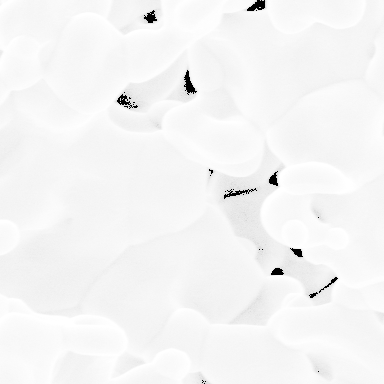

Supplement: S2 File — (ZIP) [file pone.0306385.s002.zip › S2/val/15_1-06.tif_5.png]

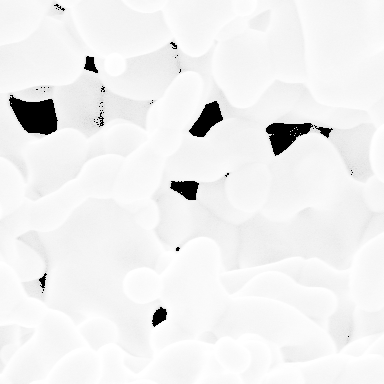

Supplement: S2 File — (ZIP) [file pone.0306385.s002.zip › S2/val/15_1-06.tif_6.png]

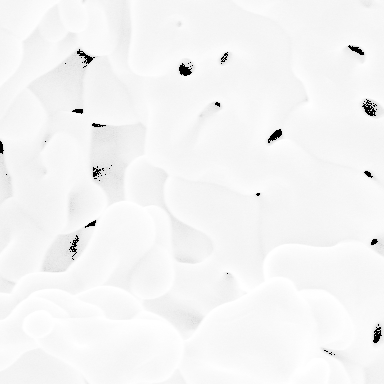

Supplement: S2 File — (ZIP) [file pone.0306385.s002.zip › S2/val/15_1-06.tif_8.png]

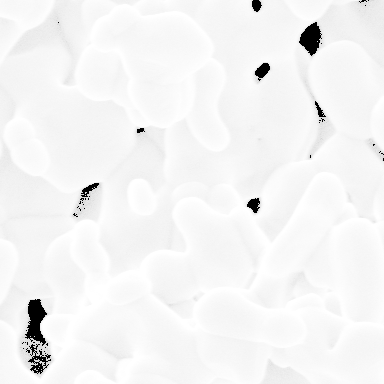

Supplement: S2 File — (ZIP) [file pone.0306385.s002.zip › S2/val/15_1-07.tif_3.png]

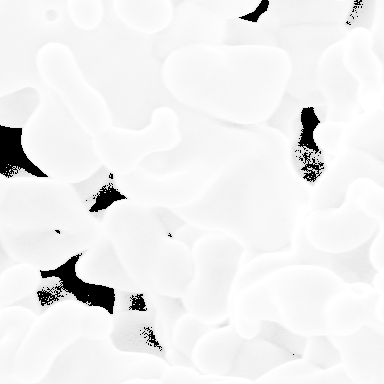

Supplement: S2 File — (ZIP) [file pone.0306385.s002.zip › S2/val/15_1-07.tif_4.png]

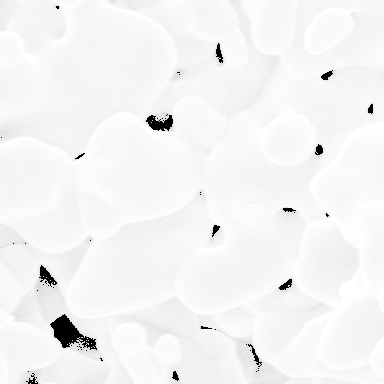

Supplement: S2 File — (ZIP) [file pone.0306385.s002.zip › S2/val/15_1-07.tif_5.png]

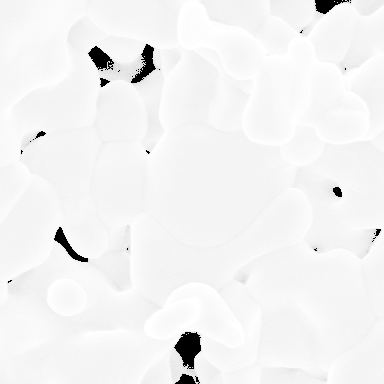

Supplement: S2 File — (ZIP) [file pone.0306385.s002.zip › S2/val/15_1-08.tif_5.png]

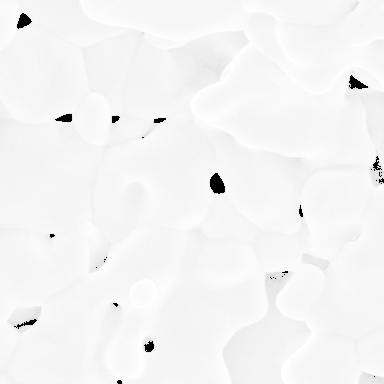

Supplement: S2 File — (ZIP) [file pone.0306385.s002.zip › S2/val/15_1-08.tif_6.png]

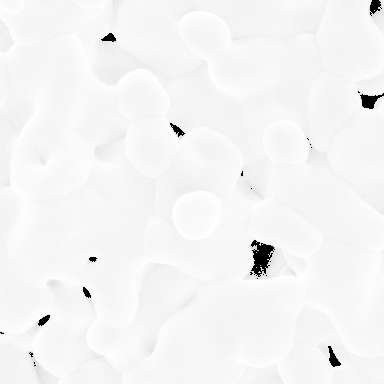

Supplement: S2 File — (ZIP) [file pone.0306385.s002.zip › S2/val/15_1-08.tif_7.png]

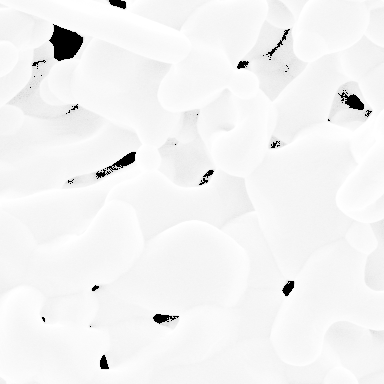

Supplement: S2 File — (ZIP) [file pone.0306385.s002.zip › S2/val/15_1-09.tif_8.png]

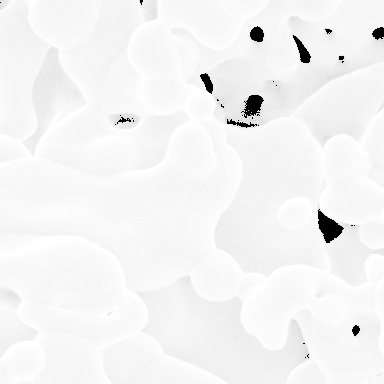

Supplement: S2 File — (ZIP) [file pone.0306385.s002.zip › S2/val/15_1-10.tif_2.png]

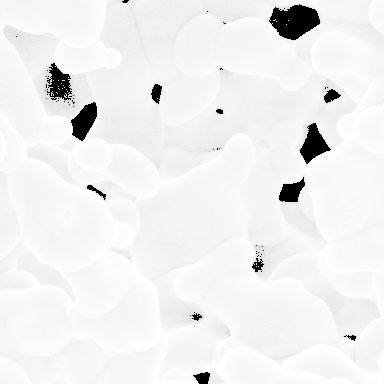

Supplement: S2 File — (ZIP) [file pone.0306385.s002.zip › S2/val/15_1-10.tif_5.png]

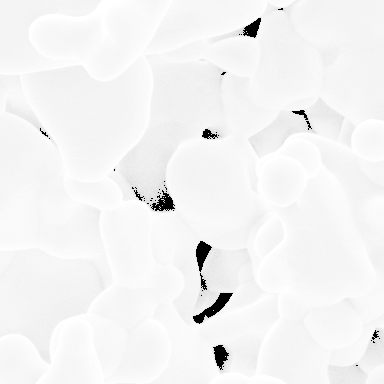

Supplement: S2 File — (ZIP) [file pone.0306385.s002.zip › S2/val/15_1-10.tif_6.png]

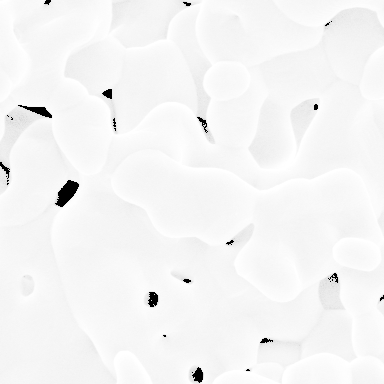

Supplement: S2 File — (ZIP) [file pone.0306385.s002.zip › S2/val/15_1-11.tif_10.png]

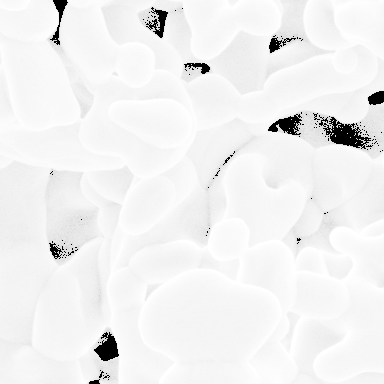

Supplement: S2 File — (ZIP) [file pone.0306385.s002.zip › S2/val/15_1-11.tif_2.png]

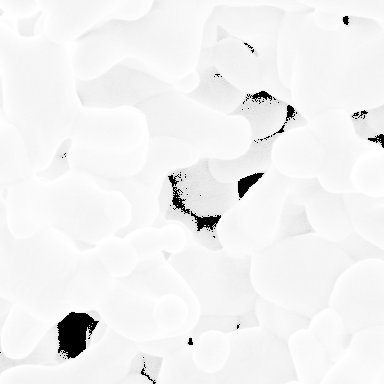

Supplement: S2 File — (ZIP) [file pone.0306385.s002.zip › S2/val/15_1-11.tif_4.png]

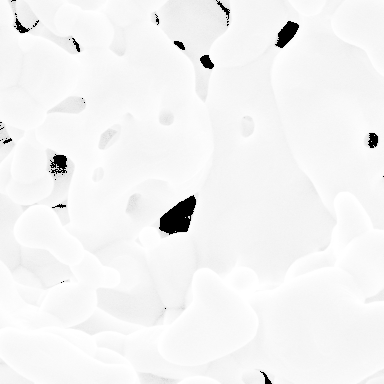

Supplement: S2 File — (ZIP) [file pone.0306385.s002.zip › S2/val/15_1-11.tif_6.png]

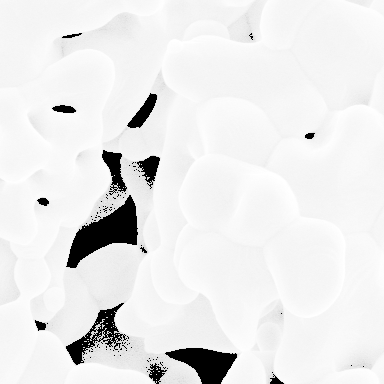

Supplement: S2 File — (ZIP) [file pone.0306385.s002.zip › S2/val/16_2-01.tif_2.png]

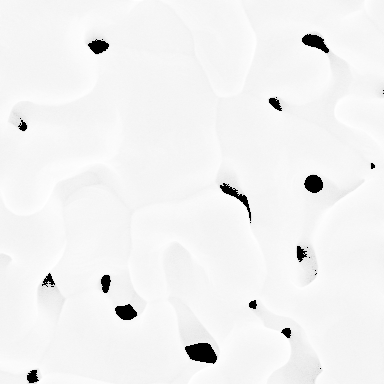

Supplement: S2 File — (ZIP) [file pone.0306385.s002.zip › S2/val/16_2-01.tif_7.png]

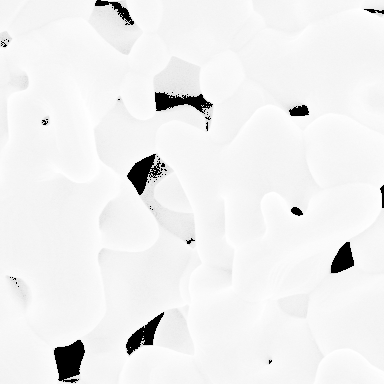

Supplement: S2 File — (ZIP) [file pone.0306385.s002.zip › S2/val/16_2-01.tif_9.png]

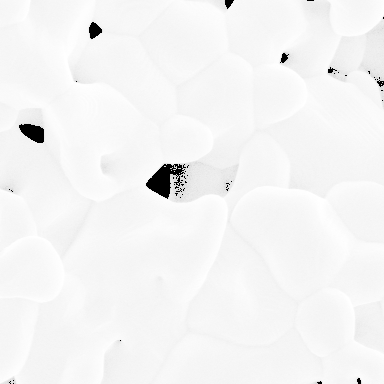

Supplement: S2 File — (ZIP) [file pone.0306385.s002.zip › S2/val/16_2_s-01.tif_1.png]

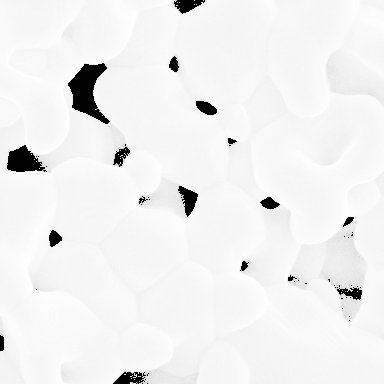

Supplement: S2 File — (ZIP) [file pone.0306385.s002.zip › S2/val/16_2_s-01.tif_3.png]

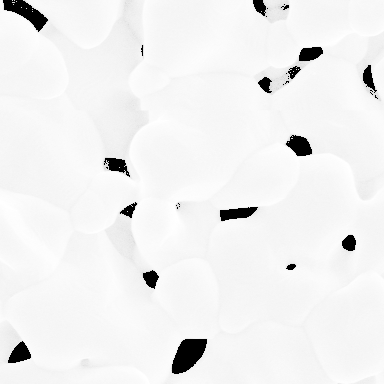

Supplement: S2 File — (ZIP) [file pone.0306385.s002.zip › S2/val/16_2_s-01.tif_4.png]

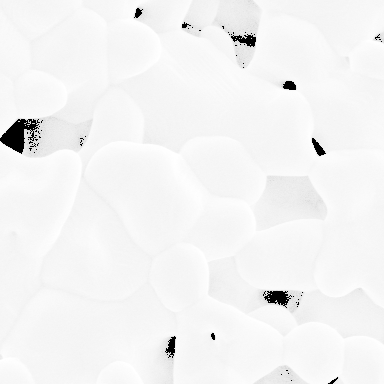

Supplement: S2 File — (ZIP) [file pone.0306385.s002.zip › S2/val/16_2_s-01.tif_5.png]

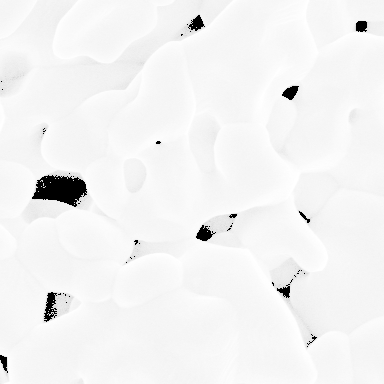

Supplement: S2 File — (ZIP) [file pone.0306385.s002.zip › S2/val/16_2_s-02.tif_1.png]

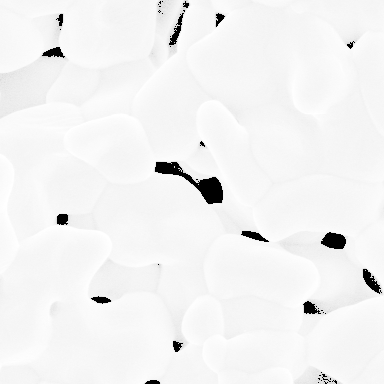

Supplement: S2 File — (ZIP) [file pone.0306385.s002.zip › S2/val/16_2_s-02.tif_10.png]

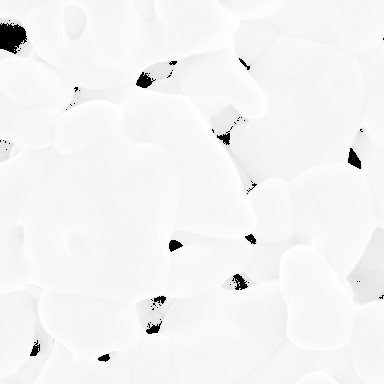

Supplement: S2 File — (ZIP) [file pone.0306385.s002.zip › S2/val/16_2_s-02.tif_8.png]

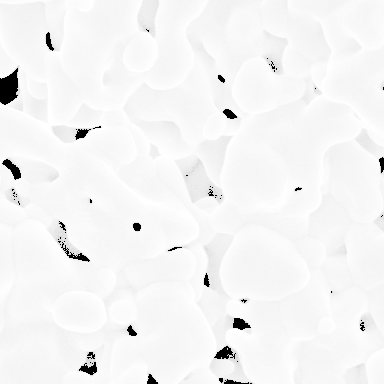

Supplement: S2 File — (ZIP) [file pone.0306385.s002.zip › S2/val/16_2_s-03.tif_2.png]

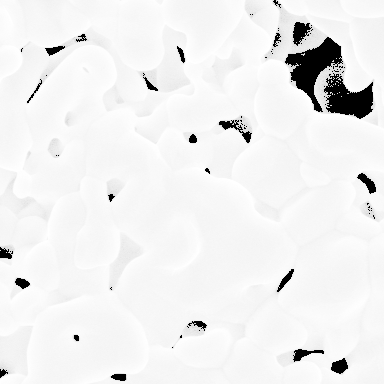

Supplement: S2 File — (ZIP) [file pone.0306385.s002.zip › S2/val/16_2_s-03.tif_3.png]

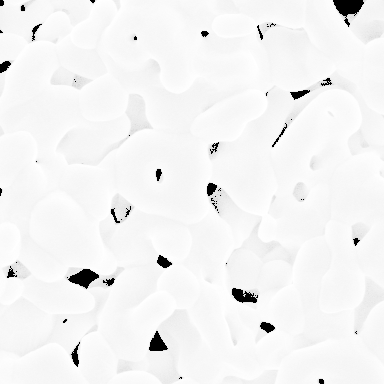

Supplement: S2 File — (ZIP) [file pone.0306385.s002.zip › S2/val/16_2_s-03.tif_7.png]

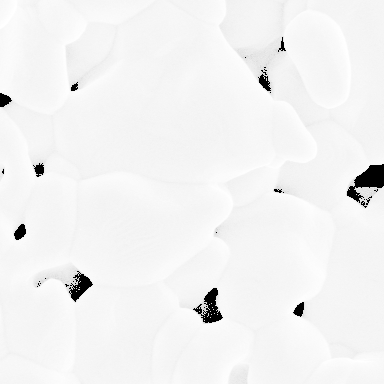

Supplement: S2 File — (ZIP) [file pone.0306385.s002.zip › S2/val/16_2_x-01.tif_10.png]

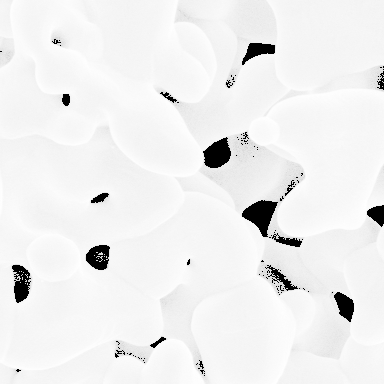

Supplement: S2 File — (ZIP) [file pone.0306385.s002.zip › S2/val/16_2_x-01.tif_3.png]

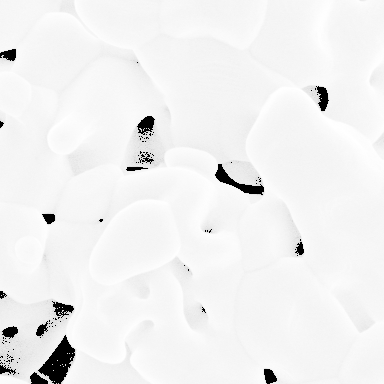

Supplement: S2 File — (ZIP) [file pone.0306385.s002.zip › S2/val/16_2_x-02.tif_1.png]

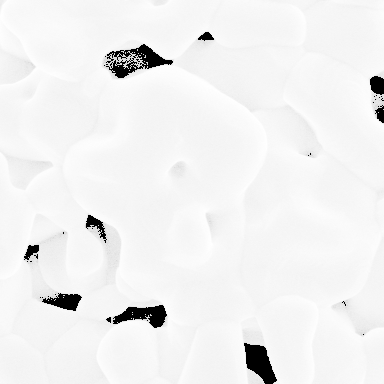

Supplement: S2 File — (ZIP) [file pone.0306385.s002.zip › S2/val/16_2_x-02.tif_2.png]

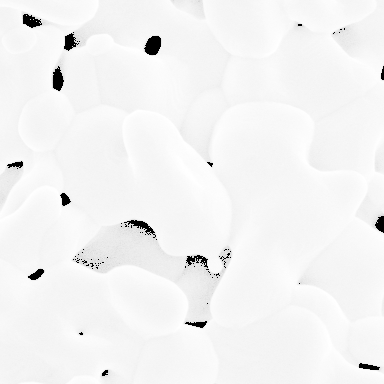

Supplement: S2 File — (ZIP) [file pone.0306385.s002.zip › S2/val/16_2_x-02.tif_4.png]

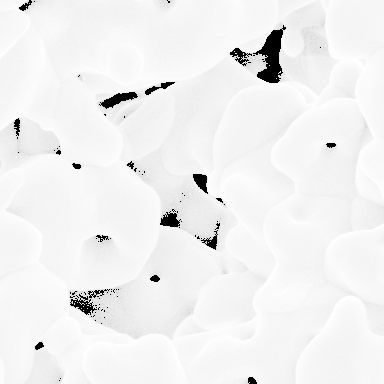

Supplement: S2 File — (ZIP) [file pone.0306385.s002.zip › S2/val/16_2_x-02.tif_6.png]

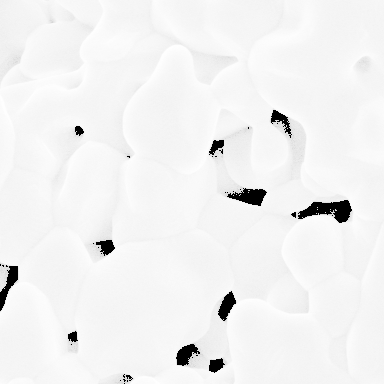

Supplement: S2 File — (ZIP) [file pone.0306385.s002.zip › S2/val/16_2_x-02.tif_9.png]

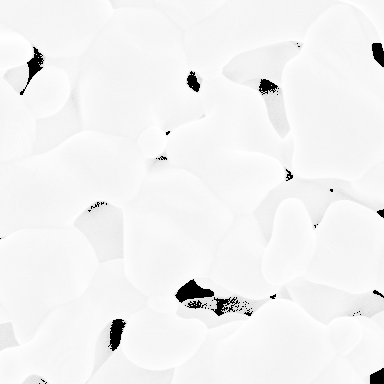

Supplement: S2 File — (ZIP) [file pone.0306385.s002.zip › S2/val/16_2_x-03.tif_1.png]
